# SPINK13 acts as a tumor suppressor in hepatocellular carcinoma by inhibiting Akt phosphorylation

**DOI:** 10.1038/s41419-024-07214-3

**Published:** 2024-11-13

**Authors:** Yongzhi Lun, Jie Sun, Ling Wei, Ben Liu, Zhixue Li, Wen Dong, Wenqi Zhao

**Affiliations:** 1 Key Laboratory of Screening and Control of Infectious Diseases, Fujian Provincial University, Quanzhou Medical College, Quanzhou, 362011 Fujian China; 2 Department of Laboratory Medicine, Putian University, Putian, 351100 Fujian China; 3 Beijing Centre for Physical and Chemical Analysis, 100089 Beijing, China; 4 College of Chemistry, Fuzhou University, Fuzhou, 350108 Fujian China

**Keywords:** Apoptosis, Mechanisms of disease

## Abstract

The PI3K/Akt pathway is overexpressed in nearly 50% of hepatocellular carcinomas and inhibits apoptosis by promoting the expression of antiapoptotic genes. Serine protease inhibitors have been shown to induce apoptosis in hepatoma cells by downregulating SPINK13 in the PI3K/Akt pathway. In this study, SPINK13 was expressed in lentiviral vectors. Changes in signaling pathway adapter proteins, apoptosis regulatory proteins, cell cycle regulatory proteins, and the biological behavior of hepatocellular carcinoma were observed in cell and nude mouse xenograft models. The underlying mechanism of endogenous SPINK13-induced apoptosis in hepatocellular carcinoma cells was explored via transcriptomics. As a result, endogenous SPINK13 might inhibit the activity of Furin protease, downregulate the Notch1/Hes1 pathway in a binding manner, activate the direct effector PTEN, inhibit Akt phosphorylation, inactivate the downstream PI3K/Akt pathway, and ultimately lead to mitochondrial apoptosis and cell cycle arrest in hepatoma cells. Therefore, the Notch1/Hes1/PTEN pathway may act upstream of SPINK13 to downregulate the PI3K/Akt signaling pathway. Our study helps elucidate the underlying mechanism of SPINK13 in anti-hepatocellular carcinoma and lays a theoretical foundation for the development of novel therapeutic serine protease inhibitors.

## Introduction

Serine protease inhibitors (serpins) are a class of active regulators involved in many physiological processes in the human body, including protein folding, blood coagulation, the inflammatory response, cell migration, fibrinolysis, complement activation, extracellular matrix reconstruction, hormone trafficking, and apoptosis. To date, only a few secretory serpins have been shown to indirectly inhibit matrix metalloproteinase (MMP) activity by directly inhibiting the serine protease activity of urokinase-type plasminogen activator (uPA) secreted by tumor cells, thereby controlling the migration and invasion of tumor cells [1, 2], such as pigment epithelium-derived factors (PEDFs), plasminogen activator inhibitor 1 (PAI-1), protease Nexin-1 (PN-1), and α1-antichymotrypsin (α1-ACT); thus, it is necessary to discover and introduce new inhibitory serpins with better therapeutic effects as tumor-targeted drugs [3].

Kazal-type serine protease inhibitor 13 (SPINK13) was first screened and discovered by our group from hepatocellular carcinoma (HCC) HepG2 cells in 2005 and named Hespintor, and the sequence was submitted to GenBank (DQ438947) in March 2006 and officially named SPINK13 in June 2018 [4]. In previous work, the expression level of SPINK13 in HCC tissues was confirmed to be significantly lower than that in non-HCC tissues and significantly lower than that in tissue samples of advanced hepatocellular carcinoma in large HCC tissues [5]. Further studies have shown that SPINK13 can significantly inhibit the invasion and migration of hepatoma cells in vitro and the subcutaneous growth of hepatoma cells in nude mouse xenograft tumors by directly inhibiting uPA activity and indirectly inhibiting the activation of MMPs by interacting with uPA in the extracellular space, and the inhibitory effect is significantly dose-dependent [4–7]. In addition, SPINK13 can induce apoptosis and activate the expression of the pro-apoptotic factors Bax and Caspase-3 while inhibiting the expression of the antiapoptotic factor Bcl-2, which means that the mitochondrial apoptosis pathway is highly likely to involve an exogenous SPINK13 pro-apoptotic mechanism [5, 7, 8]. α2-Macroglobulin (α2MR)/low-density lipoprotein receptor-associated protein 1 (LRP1) is thought to be the primary receptor for the clearance of uPA • uPA (uPA receptor (uPAR)) [9]. Since the extracellular domain of LRP1 binds to integrins, the latter can form focal adhesions (FAPs) by linking to its ligands [10], which are mostly components of the extracellular matrix (ECM). When uPA binds to uPAR alone, it promotes ECM hydrolysis on the one hand and FAP formation on the other, which in turn activates multiple intracellular signaling pathways, such as the Ras/MAPK, PI3K/Akt, and JAK/STAT pathways, and promotes cell proliferation, differentiation, adhesion, migration, and invasion [11, 12]. Through integrated analysis of the tumor tissue transcriptome, Western blotting, and cell cycle detection and verification, exogenous SPINK13 regulatory pathways were found to be enriched mainly in the phosphatidylinositol 3-kinase (PI3K)/serine-threonine protein kinase (Akt) pathway and the cell cycle pathway, which is in line with theoretical expectations. As mentioned earlier, the Serpins • uPA • uPAR complex eventually binds to the receptor α2MR/LRP1, forms endocytic vesicles through clathrin- and caveolin-mediated endocytosis, and then transports them to early endosomes to split into two parts, namely, Serpins • uPA and uPAR, which are transported by late endosomes to lysosomes for degradation and recycled to the cell surface for recycling [13, 14]. Owing to the lack of research on the metabolic outcomes and biological functions of Serpins after entry, their intracellular target proteins or target proteases and their subsequent mechanisms of action need to be studied.

This study demonstrated that the overexpression of endogenous SPINK13 was able to induce mitochondrial apoptosis and cell cycle arrest in HCC cells while inactivating the PI3K/Akt pathway. Nevertheless, unlike exogenous SPINK13, endogenous SPINK13 inhibited only Akt phosphorylation, which means that although both endogenous and exogenous SPINK13 downregulates the PI3K/Akt pathway, it causes mitochondrial dysfunction. Nevertheless, the mechanism of action of endogenous SPINK13 appears to differ from that of exogenous SPINK13. Considering that the PI3K/Akt pathway does not operate independently, it often interacts with other pathways to form a complex regulatory system and exerts biological effects. Therefore, this study aimed to preliminarily reveal the mechanism of action of the PI3K/Akt pathway by identifying endogenous SPINK13 targets and their associated upstream crosstalk pathways on the basis of confirmation of the PI3K/Akt pathway.

## Materials and methods

### Cell lines

The human hepatoma cell line HepG2, the highly invasive human hepatoma cell line MHCC97-H, and the human embryonic kidney cell line 293T were cultured in high-glucose Dulbecco’s modified Eagle’s medium (Gibco #11965118) supplemented with 1% penicillin-streptomycin (Gibco #15140148) and 10% (v/v) fetal bovine serum (Gibco #15140148), respectively. The cells were maintained at 37 °C under routine culture conditions with 5% CO_2_ and passaged once every 2 days.

### Construction of lentivirus plasmids

**Fig. 1:**
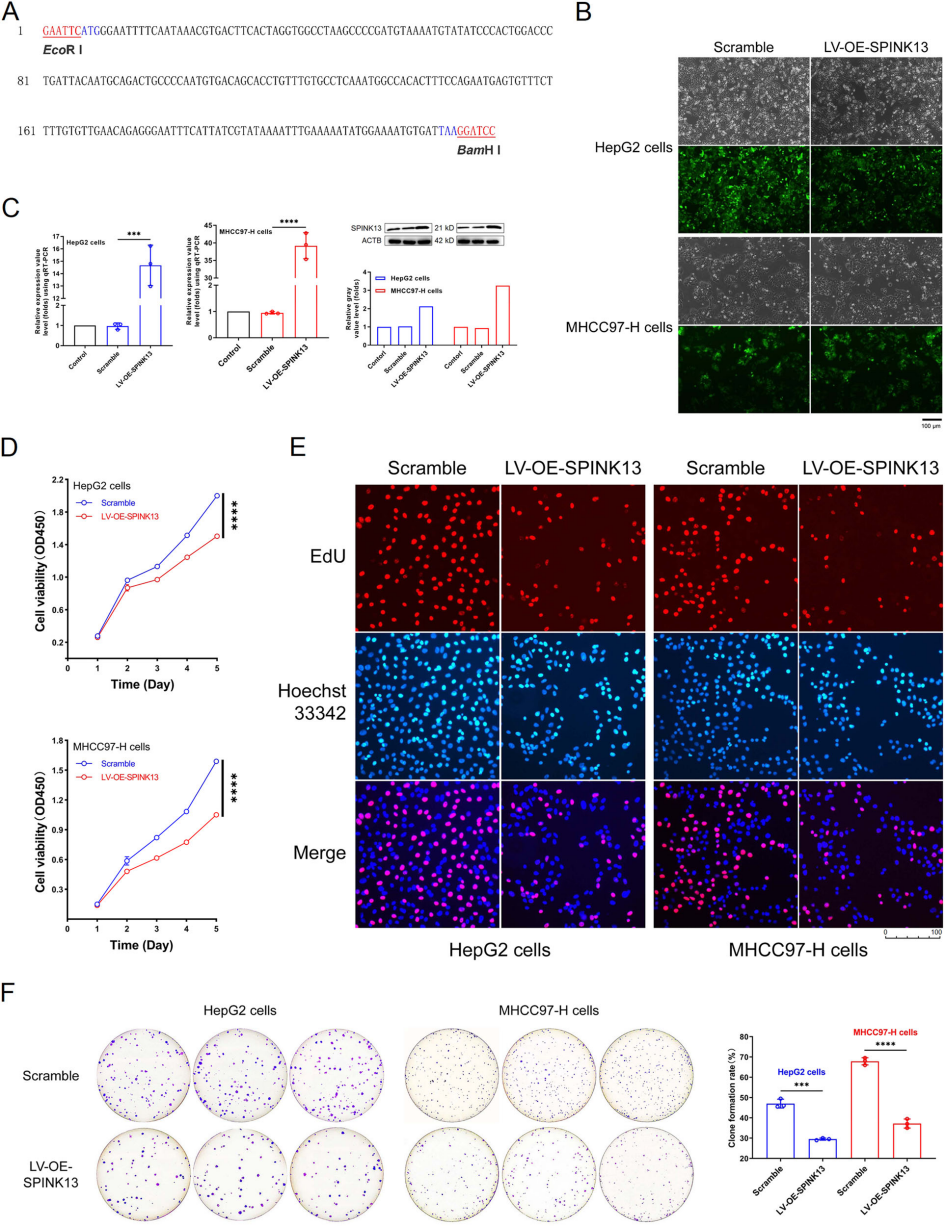
Verifying the expression of SPINK13 in stably transfected cell lines. **A** SPINK13 cDNA (with the signal peptide removed) sequence. The 5’ end was added to the EcoRI site, and the 3’ end was added to the BamHI site. **B** Establishment of stable SPINK13 gene-transfected cell lines. The recombinant lentivirus and the no-load lentivirus infected the target cells, and most of the target cells presented strong green fluorescence after three rounds of puromycin screening. Scale bars = 100 μm. **C** SPINK13 expression efficiency in stably transfected cell lines. Verification of the relative expression level of the SPINK13 gene via qRT-PCR. ***p < 0.001 and ****p < 0.0001 for cells stably transfected with recombinant lentivirus vs. cells transfected with empty lentivirus, *n* = 3. Verification of the relative expression level of SPINK13 via Western blotting. **D** SPINK13 inhibited the proliferative ability of stably transfected cell lines via overexpression. Compared with those of the scramble group, the cell viability and proliferation of the LV-OE-SPINK13 group were significantly inhibited. ns (not significant), *p < 0.05 and ****p < 0.0001, *n* = 3. **E** SPINK13 inhibition of DNA synthesis by SPINK13 overexpression in stably transfected cell lines. EdU assays were performed to detect the viability and proliferation of HepG2 and MHCC97-H cells transfected with a lentivirus encoding SPINK13. Scale bars = 100 μm. **F** SPINK13-mediated inhibition of the clonogenic ability of stably transfected cell lines by overexpression. Compared with the scramble group, the ability to colonize the LV-OE-SPINK13 group was significantly inhibited. *p* < 0.001 and ****p < 0.0001, *n* = 3.

The SPINK13 cDNA (with the signal peptide removed) sequence was synthesized by Sangon Biotech (Shanghai, China) Co., Ltd., with the 5’ end plus the EcoR I (Takara #1040S) site (GAATTC) and the 3’ end plus the BamH I (Takara #1010S) site (GGATCC) (Fig. 1A) and cloned and inserted into the pLVX-mCMV-ZsGreen1-Puro plasmid. The ligation product was subsequently transformed into *Escherichia coli* DH5α competent cells. The bacterial mixture was evenly spread on LB plates (Beyotime #ST158) containing 50 μg/ml ampicillin (Beyotime #ST007). After being cultured overnight, positive clones were picked for culture, and the Plasmid Mini Preparation Kit (Beyotime #D0003) was used to extract the plasmids. The enzyme digestion products were identified and sent for correct sequencing.

### Lentivirus preparation

A Plasmid Mini Preparation Kit with Magnetic Beads (Beyotime #D0075S) was used to extract the endotoxin-free pLVX-mCMV-ZsGreen1-Puro-SPINK13 recombinant plasmid, the lentiviral shuttle plasmid psPAX2 and the auxiliary packaging original vector plasmid pMD2G. When the confluency rate of the 293T cells reached 50–70%, 10 ml of fresh high-glucose Dulbecco’s modified Eagle’s medium supplemented with 5% (v/v) fetal bovine serum was added, and the mixture was incubated for 1–2 h. psPAX2 (8.34 μg), pMD2. G 2.52 μg, pLVX-mCMV-ZsGreen1-Puro-SPINK13 8.14 μg, Opti-MEM medium (Gibco #31985070) up to 500 μl, 15 ml centrifuge tube (No. 1 tube) and Lipo6000 Transfection Reagent (Beyotime #C0526) 57 μl, 443 μl of Opti-MEM culture medium, placed in a 15 ml centrifuge tube (No. 2 tube) and mixed thoroughly. The above transfection reagents were allowed to stand for 5 min at room temperature, and the No. 2 tube reagent was added to the No. 1 tube, mixed thoroughly, and allowed to stand at room temperature for 15 min, after which a total of 293T cells were transfected. After 24 h of transfection, the medium was changed to a complete medium. The cell supernatant enriched with lentiviral particles was collected for a duration of 48 h, followed by collection of the cell supernatant again after adding a fresh complete medium for an additional 24 h. A high-titer lentiviral concentrate was obtained by efficiently concentrating the viral supernatant collected twice via an Amicon Ultra15 centrifugal ultrafiltration tube 100 kDa (Millipore #UFC901024). In addition, the pLVX-mCMV-ZsGreen1-Puro no-load lentivirus was prepared as a control.

### Lentiviral titer assay

The lentiviral titers were determined via a well-by-well dilution method. 293T cells were seeded into 96-well plates, the cell density was controlled at 3 × 10^4^ cells/well, and the cells were incubated at 37 °C and 5% CO_2_ for 24 h. The lentiviral stock was diluted 8-fold with Dulbecco’s modified Eagle’s medium supplemented with 10% (v/v) fetal bovine serum 10-fold. The culture medium was aspirated, 100 μl of virus mixture was added to each well according to the gradient, and a blank control group was established. The virus mixture was aspirated, 100 μl of culture medium was added to each well, the mixture was incubated for 72 h, the cells were observed under a fluorescence microscope, and the total number of fluorescent cells in the last two fluorescent dilution gradient wells was counted, X and Y were set, the lentivirus titer (TU/ml) was calculated as follows: ((X + Y × 10) × 10^6^)/(2 × X wells’ virus content (μl)), and the average value of the double wells was calculated as the lentivirus stock titer.

### Screening of stable lentiviral-transfected cell lines

The multiplicity of infection (MOI) value (number of virus particles/number of target cells) was determined by a preexperiment, and a lentiviral dose with an MOI of 20 was selected to infect the target cells. The optimal screening concentration of puromycin dihydrochloride (Beyotime #ST551) was 2 μg/ml, which was continuously screened for 3 generations and used for follow-up experiments. SPINK13 overexpression efficiency was detected via qRT-CR and Western blot analysis. For the subsequent intervention experiment with the Akt agonist SC79, lentivirus stably transfected cell lines were collected and seeded into 6-well plates. The cell density was controlled at 5 × 10^5^/ml, 37 °C, 5% CO_2,_ and saturated humidity, and when the cell confluency was 80%, SC79 (Sigma-Aldrich #305834-79-1) at a concentration of 8 μg/ml was added, and the mixture was further cultured for another 24 h.

### qRT-PCR

Total RNA was extracted from tissues or cells according to the instructions of the PureLink Total RNA Kit (Thermo Fisher Scientific #12183018A). GAPDH was used as an internal reference gene. The relative expression of target mRNAs was detected via qRT-PCR and the 2^−ΔΔCt^ method. With total RNA as a template, reverse transcription was carried out according to the instructions of the Hifair II 1st Strand cDNA Synthesis Kit (Yeasen #11119ES60), and the obtained product was cDNA, which was diluted 10 times and used for subsequent qPCR. The reaction system was as follows: Hieff UNICON Universal Blue qPCR SYBR Green Master Mix (Yeasen #11184ES03) 5.0 μl, cDNA 2.0 μl, forward primer (10 μM) 0.5 μl, reverse primer (10 μM) 0.5 μl, and DNase-free dH_2_O up to 10.0 μl. The reaction conditions were as follows: 95 °C for 2 min, 95 °C for 10 s, and 60 °C for 30 s (40 cycles). The primers were synthesized by Sangon Biotech (Shanghai, China) Co., Ltd., and the sequences are shown in Table S1.

### Western blot

For cultured cells, cell lysis buffer for Western and IP (Beyotime #P0013) was added, and after adequate lysis, the cells were collected in an ice bath for 30 min, followed by centrifugation at 14,000 × *g* for 3 min to retain the supernatant. The tumor tissue was cut into fine pieces, and 1 ml of RIPA lysis buffer (Beyotime #P0013B) with protease and phosphatase inhibitor cocktail for mammalian cell and tissue extracts (Beyotime #P1051) per 100 mg of tissue was added. The mixture was homogenized 20 times with a glass homogenizer, the homogenate was collected, the mixture was centrifuged at 12,000 rpm and 4 °C for 5 min, and the supernatant was retained.

The protein content of the supernatant was quantified via a BCA protein assay kit (Beyotime #P0012S) with BSA as the standard. A volume of 20 μl of the supernatant was combined with 25 μl of 5×SDS loading buffer and 5 μl of β-mercaptoethanol, followed by boiling for 5 min. The samples were loaded after instantaneous high-speed centrifugation, SDS‒PAGE, 60 V constant pressure electrophoresis, and 60 V constant pressure transfer on ice for 100 min. The PVDF membrane (Beyotime #FFP24) was blocked at room temperature for 2 h, the primary antibody (monoclonal antibody, 1:1000 dilution) was incubated at room temperature for 60 min, followed by overnight incubation at 4 °C, and the secondary antibody (HRP-sheep anti-rabbit IgG, dilution 1:1000) was incubated at room temperature for 60 min. The PVDF membrane was developed with a chemiluminescence kit, and the bands were quantified with the image analysis software ImageJ. The relative expression of the target protein was calculated as the gray value of the target protein band/the gray value of the housekeeping protein band. In the present study, rabbit anti-human PI3K (#ab109006), p-PI3K (phosphorylation site Tyr458 and Tyr199) (#ab278545), Akt (#ab179463), p-Akt (phosphorylation site Ser473) (#ab81283), Bax (#ab32503), Bcl-2 (#ab182858), cleaved caspase-3 (Asp175) (#ab32042), cleaved caspase-9 (Asp353) (#ab2324), P-Rb (phosphorylation site Ser807) (#ab184796), CyclinD1 (#ab16663), Cyclin E1 (#ab133266), CDK2 (#ab101682), CDK4 (#ab199728), CDK6 (#ab124821), cleaved Notch1 (Val1744) (#ab52627), Hes1 (#ab108937), PTEN (#ab267787), E-cadherin (#ab40772), N-cadherin (#ab76011), vimentin (#ab92547), β-actin (ACTB) (#ab213262) monoclonal antibodies (primary antibodies) and goat anti-rabbit IgG-HRP (#ab6721) (second antibodies) were purchased from Abcam. In addition, rabbit anti-human SPINK13 (#PA5-57668), SuperSignal West Pico PLUS Chemiluminescent substrate (#34579), and PageRuler Prestained Protein Ladder (#26617) were purchased from Thermo Fisher Scientific.

### CCK8 assay

Logarithmic growth phase cultured cells were seeded in 96-well cell culture plates at a density of 1000 cells/100 μl per well, with 3 compound wells and blank wells in each group. After incubation, 10 μl of Cell Counting Kit-8 solution (Beyotime #C0037) was added to each well, and the absorbance value at 450 nm was determined with a microplate reader. Cell viability was calculated as follows: (OD experimental group-OD blank group)/(OD control group-OD blank group) × 100%.

### Colony formation experiments

Logarithmic growth phase cultured cells were suspended in a complete culture medium containing 10% fetal bovine serum for later use. Each group of cells was seeded in 6-well plates, 200 cells per well were dispersed evenly, and each group of cells was set up with 3 double wells and cultured at 37 °C, 5% CO_2_, and saturated humidity for 1–2 weeks. When there were visible clones in the Petri dish, the culture was terminated, the supernatant was aspirated carefully and soaked in PBS twice, 4% paraformaldehyde was added to fix for 20 min, crystal violet staining was performed for 20 min, the staining solution was slowly washed off with water, and photographs were taken. The colony formation rate was calculated as follows: (number of clones/number of seeded cells) × 100%.

### 5-Ethynyl-2’-deoxyuridine (EdU) assay

The instructions for the EdU Cell Proliferation Kit with Alexa Fluor 647 (Beyotime #P0013) were followed. The cells were cultured during the logarithmic growth phase and seeded into 6-well plates. The cells were cultured in the logarithmic growth phase and seeded into 6-well plates at a controlled density of 2 × 10^4^ cells/well. After overnight incubation at 37 °C, each well received an equal volume of prewarmed EdU working solution (final concentration of 10 μM), followed by incubation for 2 h. The culture medium was aspirated, and the samples were fixed at room temperature with 4% paraformaldehyde for 15 min. The fixative solution was aspirated, and the samples were washed twice with PBS buffer containing 3% BSA for 3–5 min each. The wash mixture was aspirated, PBS buffer containing 0.5% Triton X-100 was added, and the mixture was incubated for 15 min at room temperature. The permeabilization solution was aspirated, the mixture was washed twice, 0.5 ml of Click reaction solution (Apollo fluorescent dye) was added to each well, and the mixture was incubated for 30 min at room temperature in the dark. The reaction mixture was aspirated, the mixture was washed twice, Hoechst 33342 solution was added, and the mixture was incubated for 10 min at room temperature in the dark. The Hoechst 33342 solution was aspirated, the samples were washed 2 times, and laser confocal detection was performed. Hoechst 33342 (blue fluorescence) was used for excitation at 346 nm and emission at 460 nm, and EdU 647 (red fluorescence) was used for excitation at 643 nm and emission at 662 nm.

### Apoptosis assays

Following the instructions of the Annexin V-PE/7-AAD Apoptosis Assay Kit (Yeasen #40310ES20), 5 μl of 7-AAD stain solution was added to 50 μl of 1× binding buffer and mixed well. A total of 5 × 10^5^ logarithmic growth phase cultured cells were collected, the above 7-AAD stain solution was added, and the mixture was incubated at room temperature in the dark for 5–15 min. Then, 450 μl of binding buffer and 5 μl of Annexin V-PE were added to the mixture well, and the mixture was allowed to react at room temperature in the dark for 5–15 min. Flow cytometry was completed within 1 h after the reaction ended, with an excitation wavelength of 488 nm and an emission wavelength of 578 nm.

### ROS (reactive oxygen species) assay

The instructions of the ROS test kit were followed (Applygen #C1300-2). The cells were cultured during the logarithmic growth phase and seeded into 6-well plates; the cell density was adjusted to 5 × 10^4^ cells/well, and the plates were incubated overnight at 37 °C. The cell suspensions were collected and washed 2 times with PBS, and the supernatant was aspirated. The cell pellet was resuspended with a diluted dihydroethidium (DHE) fluorescent probe (final concentration of 10 μM) in a serum-free medium, and the cell density was controlled at 2 × 10^7^ cells/ml. The mixture was incubated at 37 °C for 30 min and centrifuged at 1000 × *g* for 5 min. The supernatant was aspirated, washed twice, and detected by flow cytometry, with an excitation wavelength of 480–535 nm and an emission wavelength of 590–610 nm.

### Cell cycle assays

The instructions of the Cell Cycle and Apoptosis Analysis Kit (Beyotime #C1052) were followed. A total of 5 × 10^5^ logarithmic growth phase cultured cells were collected, 1 ml of ice bath prechilled with 70% ethanol was added, and the mixture was fixed at 4 °C for more than 12 h. The cells were pelleted by centrifugation, washed 1 time with approximately 3 ml of ice bath prechilled with PBS, the supernatant was aspirated, the supernatant was aspirated, and the cells were appropriately dispersed to prevent clumping. Then, 0.5 ml of propidium iodide staining solution was added to each tube of the cell samples, the cell pellet was slowly and thoroughly resuspended, and the mixture was stored at 37 °C for 30 min in the dark and then at 4 °C in the dark. Flow cytometry was completed within 24 h after the staining was complete, and an excitation wavelength of 488 nm was used to detect red fluorescence and light scattering.

### Animal experiments

Animal experiments were conducted in accordance with the UK Animals (Scientific Procedures) Act 1986 and related guidelines, and the experimental protocol was approved by the Ethics Committee of Putian University. Sixteen SPF nude mice, 6–8 weeks old, male, weighing 22 ± 2–3 g, were purchased from Ensiweier Biotechnology (Chongqing, China) Co., Ltd., and were randomly divided into two groups, the LV-OE-SPINK13 group and the scramble group, with 8 mice in each group. SPINK13 stable overexpression and normal MHCC97-H cells were prepared as a cell suspension at a concentration of 1 × 10^7^/ml. Each nude mouse was slowly inoculated with 200 μl of cell suspension under the skin of the right axilla and observed for 4 weeks after tumorigenesis. The health status and tumor growth of the nude mice were observed every day. After the nude mice in each group were transplanted, the tumors formed, the length and short diameter of the tumors were measured on the following day, and the tumor volume was calculated as follows: (long diameter × short diameter^2^)/2. Two days after the last administration, 0.3% sodium pentobarbital solution was used for intraperitoneal anesthesia (0.2 ml/piece), and after anesthesia, the skin around the tumor was removed, the tumor mass was removed, the volume was measured, weighed, and the tumor inhibition rate was calculated as follows: ((average tumor volume of the control group-average tumor volume of the experimental group)/average tumor volume of the control group) × 100%, ((average tumor weight of the control group-average tumor weight of the experimental group)/average tumor weight of the control group) × 100%.

### TUNEL assay

Following the instructions of the In Situ Cell Death Detection Kit, POD (Roche #11684795910), the tumor tissue was collected, washed with PBS, fixed with 4% paraformaldehyde, and subjected to dehydration, transparency, paraffin embedding, sectioning, baking, and other steps, after which the tissue sections were deparaffinized and rehydrated. Proteinase K working solution was added, and the mixture was incubated at 37 °C for 15–30 min. The tissue was washed twice in PBS and dried. Fifty microlitres of TUNEL reaction solution (TdT enzyme:label solution = 1:9) was added to each sample, which was subsequently incubated at 37 °C with a wet box cap for 60 min. The sample was subsequently washed 3 times with PBS and dried (only the label solution was added to the control sample), 50 μl of Converter-POD was added, and the sample was incubated at 37 °C with a lid on a wet box for 30 min. DAB was used to stain the sample at room temperature for 10 min, nuclear staining was performed with hematoxylin staining solution, and microscopic examination was carried out with a neutral resin mount. Under the microscope, brownish-yellow particles appeared in the apoptotic cells. The color was randomly observed, the number of positive cells was counted, and the positive cells were scored via a semiquantitative method (the two scores were multiplied): negative, 0 points; positive, 1–4 points; and strongly positive, >4 points. The proportion of positive cells (score of 1) was as follows: <10%, 0 points; 10–50%, 1 point; 51–75%, 2 points; and >75%, 3 points. Color (score of 2) was scored as follows: no color, 0 points; light yellow, 1 point; yellow, 2 points; and brown, 3 points.

### RNA-Seq analysis

Three tumor samples from the nude mice were randomly selected from the LV-OE-SPINK13 group and the scramble group. These samples were immediately placed in liquid nitrogen and then quickly transferred to −80 °C for preservation. RNA extraction and testing, library construction, and computer sequencing of the fresh tissue samples were performed by Western Biotechnology Co., Ltd. (Chongqing, China). Illumina PE150 sequencing was performed following the library inspection. After the original sequencing data yielded were qualitatively evaluated, the reads were compared to the reference genome (Ensembl 75) via Hisat v2.0.5, the transcripts were spliced via StringTie v2.1.1, unqualified spliced transcripts were removed via Cuffmerge v2.2.1, and finally, differential expression significance analysis was performed via Cuffdiff v2.2.1.

### Coexpression analysis of the differentially expressed genes

The screening criteria for the differentially expressed genes were as follows:│log FC│ ≥ 1, *p* < 0.05, adj. *p* < 0.05. The differentially expressed genes (DEGs) identified through this screening process were subjected to enrichment analysis of the Kyoto Encyclopedia of Genes and Genomes (KEGG) pathway and Reactome pathways via the Metascape database (http://Metascape.org). The enrichment standards were Min Overlap ≥3, *p* value cutoff ≤0.01, and default values for the remaining variables. The screened differentially expressed genes were submitted to the STRING online tool (http://string-db.org) to construct a visualized interaction network. The parameters were set to the mean of the network edges: confidence; active interaction sources: text mining, experiments, and databases; minimum required interaction score ≥0.7 (full score: 1); hide disconnected nodes in the network; and select the default value for the remainder.

### Construction of the regulatory network of target genes

The gene set with the interaction relationship was imported into Cytoscape software to construct a visualized network. First, the core genes that formed the stable structure of the network were screened via the MCODE plug-in. The parameters were set as follows: degree cutoff ≥3, K-core ≥4, and default values for the remainder. The topological characteristics of the network and each node were subsequently calculated via a Centiscape plug-in. The genes corresponding to nodes with a degree value ≥ mean + SD were considered hub genes, and the genes corresponding to nodes with a betweenness value ≥ mean + SD were considered bottleneck genes. The main pathway names were subsequently summarized and obtained via annotation by taking the intersection of the three genes as the target gene. The mutual relationship text was built and imported into Cytoscape software to construct the visual regulatory network.

### Molecular docking

The X-ray crystal structure of Furin (4Z2A) was obtained from the protein database (https://www.rcsb.org), and the SPINK13 structure was predicted via the online tool Alphafold 2 (https://alphafold.ebi.ac.uk) [15]. To ensure the accuracy of the docking results, AutoDockTools 1.5.7 was then used to manually perform optimization operations such as dehydration and hydrogenation of the two protein structures [16], and then the docking server GRAMM (https://gramm.compbio.ku.edu) was used for protein-protein docking [17, 18]. The obtained protein-protein complexes were also optimized via AutoDockTools 1.5.7 for manual dehydration and hydrogenation, after which PyMOL was used to predict protein-protein interactions and generate a protein-protein interaction map.

### Construction of eukaryotic expression vectors

The preconstructed pLVX-mCMV-ZsGreen1-Puro-SPINK13 recombinant plasmid was used to obtain the Flag-SPINK13 gene fragment via PCR, and the HA-Furin gene fragment was obtained via the whole gene synthesis method. The Hind III (Takara #1060S) site was added to the 5’ end, the *EcoR* I site was added to the 3’ end, and the pcDNA3.1 plasmid was cloned. The ligation products were subsequently transformed into *Escherichia coli* DH5α competent cells. The bacterial mixture was evenly spread onto LB plates containing 50 μg/ml ampicillin and cultured overnight. Positive clones were picked for culture via Plasmid Mini Preparation Kit extraction of plasmids whose enzyme digestion identification was correct and sent for sequencing.

### Subcellular colocalization assay

A total of 0.5 μg each of pcDNA3.1-Flag-SPINK13 and pcDNA3.1-HA-Furin were cotransfected into MHCC97-H cells cultured in 24-well plate crawlers, incubated overnight at 37 °C, and then replaced with fresh complete medium. The pcDNA3.1-Flag-SPINK13 single transfectants and pcDNA3.1-HA-Furin single transfectants were used as control groups. After 48 h of transfection, the cells were treated with 0.5% Triton X-100 for 20 min at room temperature and immersed in PBS 3 times for 3 min each. Identification was performed with a three-color fluorescent marker, with yellow-green FITC staining for SPINK13, orange-red Cy3 staining for Furin, and blue 4,6-diamidino-2-phenylindole (DAPI) staining for nuclei. The primary antibody was incubated with mouse anti-Flag (Affinity #T0003) antibody and rabbit anti-HA (Affinity #T0050) antibody (1.5 h at room temperature) at a dilution ratio of 1:100. After rinsing with PBS, and the secondary antibodies goat anti-mouse IgG H&L (FITC) (Abcam #) and goat anti-rabbit IgG H&L (Cy3) (Abcam #) were incubated at a dilution ratio of 1:800. After rinsing in PBS, 5 mg/L DAPI (Beyotime #C1006) was added dropwise and incubated at room temperature for 5 min. After rinsing in PBS, the slides were mounted with an antifluorescence quencher (Beyotime #P0128M) and placed under a laser confocal fluorescence microscope for imaging.

### Coimmunoprecipitation

MHCC97-H cells were plated into two cell culture dishes (100 mm). When the confluency of the monolayer reached 80%, pcDNA3.1-Flag-SPINK13 was cotransfected with pcDNA3.1-HA-Furin and pcDNA3.1 into MHCC97-H cells, the cell culture medium was discarded, and the cells were collected after 24 h. One ml of cell lysis buffer for Western blotting. IP was added, and the mixture was lysed in an ice bath for 20 min and centrifuged at 13,000 × *g* for 10 min, after which the protein concentration of the supernatant was determined. The protein concentration of the cell lysate was diluted with IP lysis buffer to 2 mg/ml, 500 μl was added to a centrifuge tube containing anti-Flag antibody-conjugated NHS-activated magnetic beads (Pierce #88826), and the mixture was incubated on a rotator for 2 h at room temperature. Next, 500 μl of IP lysis buffer was added to the tube, which was mixed gently. The beads were collected using a magnetic stand, the supernatant was removed, and the procedure was repeated. Next, 500 μl of ultrapure water was added to the tube, and the process was repeated. Next, 100 μl of elution buffer was added to the tube, which was subsequently incubated on a rotator for 5 min at room temperature. The magnetic beads were collected via a magnetic stand, the supernatant containing the antigen of interest was retained, and SDS‒PAGE protein loading buffer was added and boiled for Western blot detection.

### Construction of bimolecular fluorescent complementary vectors

The recombinant plasmids pcDNA3.1-Flag-SPINK13 and pcDNA3.1-HA-Furin were used to obtain SPINK13 and Furin gene fragments, respectively, via PCR. The pBiFC-VN173-Flag vector and the SPINK13 gene fragment were subjected to EcoR I and Hind III double digestion to obtain the SPINK13 gene fragment and the linearized vector fragment containing specific sticky ends, and the pBiFC-VC155-HA vector and Furin gene fragment were subjected to EcoR I and Sal I double digestion to obtain the Furin gene fragment and the linearized vector fragment containing specific sticky ends, after which the two fragments were ligated. The above two ligation products were transformed into *Escherichia coli* DH5α competent cells. The bacterial mixture was evenly spread onto LB plates containing 50 μg/ml ampicillin and incubated overnight. Positive clones were picked for culture, and the plasmid was extracted via a Plasmid Mini Preparation Kit, identified via enzyme digestion, and subjected to sequencing.

### Bimolecular fluorescence complementation (BiFC) assay

A total of 0.5 μg of each sequence-corrected pBiFC-VN173-Flag-SPINK13 or pBiFC-VC155-HA-Furin was cotransfected into MHCC97-H cells cultured in 24-well plates, incubated at 37 °C overnight, and replaced with fresh complete medium. The pBiFC-VN173-Flag+pBiFC-VC155-HA, pBiFC-VN173-Flag+pBiFC-VC155-HA-Furin, and pBiFC-VN173-Flag-SPINK13+pBiFC-VC155-HA cotransfected cells were used as the control group. After 48 h of transfection, the cells were subjected to laser confocal fluorescence microscopy.

### Furin enzyme activity assay

Furin Enzyme Activity Assay: The Furin-specific short peptide substrate Cbz-Arg-Ser-Lys-Arg-AMC was synthesized by China Peptides (QYAOBIO) Co., Ltd. (Shanghai, China). AMC, when covalently bonded, does not emit fluorescence; however, upon hydrolysis of the short peptide substrate by Furin, AMC is released and can be excited to emit fluorescence. The intensity of the fluorescence emitted from the free AMC correlates with the activity of the Furin enzyme. The control group (MHCC97-H cells), the scramble group, and the LV-OE-SPINK13 group cells were cultured normally, cell lysis buffer for Western and IP was added to each group of cells, and protein samples were prepared according to the instructions. The protein concentration was determined via the BCA method, and the process was repeated in triplicate. Protein samples (30 μg) were added to 200 μl of the system with a final concentration of 200 μM furin-specific substrate and incubated at 37 °C for 1 h in the dark. The fluorescence intensity was detected via a fluorescence microplate reader at an excitation wavelength of 380 nm, and an emission wavelength of 460 nm, and the results were recorded and analyzed.

### Expression of the Notch1/Hes1/PTEN pathway in hepatocellular carcinoma tissues

HCC transcriptome data were downloaded from the TCGA database, and samples from paired adjacent tissues were included in the study. The GEO database was searched to download the human HCC expression profile dataset, and the samples were required to be liver cancer tissue and normal tissue or liver cancer tissue paired with adjacent tissue. The differential expression of Notch1, Hes1, and PTEN in liver cancer and adjacent tissues in each set of data was calculated via Student’s *t* test, and a differential expression box plot was drawn via the R software ggplot2 package.

### Statistical analysis

Statistical analysis was performed with GraphPad Prism 9.3 software (San Diego, CA, USA). For comparisons between two datasets, the Student’s *t* test was used. For the analysis of three or more sets of data, ANOVA was used. All data in the figure are presented as the means and standard deviations, and the differences were statistically significant (*p < 0.05, **p < 0.01, ***p < 0.001 and ****p < 0.0001).

## Results

### The overexpression efficiency of SPINK13 in lentiviruses

The recombinant and empty lentiviral titers reached 2.8 × 10^8^ TU/ml and 2.6 × 10^8^ TU/ml, respectively. The above lentiviruses were then used to infect HepG2 and MHCC97-H cells, and after 3 rounds of puromycin screening, the majority of the target cells presented strong green fluorescence, indicating that the lentiviral particles efficiently infected the target cells (Fig. 1B). The efficiency of SPINK13 expression was confirmed by qRT-PCR and Western blot. The SPINK13 mRNA level in the LV-OE-SPINK13 group (OE-SPINK13 group) was 14.8-fold (HepG2 cells) and 39.21-fold (MHCC97-H cells) greater than that in the scramble group, and the SPINK13 protein level increased 2.13-fold (HepG2 cells) and 3.25-fold (MHCC97-H cells), respectively, whereas the expression level in the scramble group was consistent with that in the control group (Fig. 1C).

Compared with the scramble group, both LV-OE-SPINK13 groups presented a significant decrease in cell number over time, and the rate of apoptosis accelerated daily when SPINK13 was overexpressed (Fig. 1D). EdU, as a thymine nucleoside analog, can be inserted into replicating DNA instead of thymine during DNA synthesis and then form a special and stable triazole ring structure through the conjugation of the acetyl group with Apollo. The conjugation reaction between the acetylenic group and Apollo fluorescent dye results in the formation of a special and stable triazole ring structure, which can efficiently and accurately reflect the replication activity of DNA, thus efficiently detecting cell proliferation. Laser confocal microscopy revealed that the proportion of cells in the LV-OE-SPINK13 group in the DNA synthesis phase (red fluorescence) was significantly lower than that in the scramble group (Fig. 1E). The colony formation rate also reflects the cell proliferation ability. Compared with the scramble group, the LV-OE-SPINK13 group overexpressing SPINK13 presented a significant decrease in colony formation ability (Fig. 1F).

### Effect of SPINK13 overexpression on apoptosis in hepatocellular carcinoma cells

**Fig. 2:**
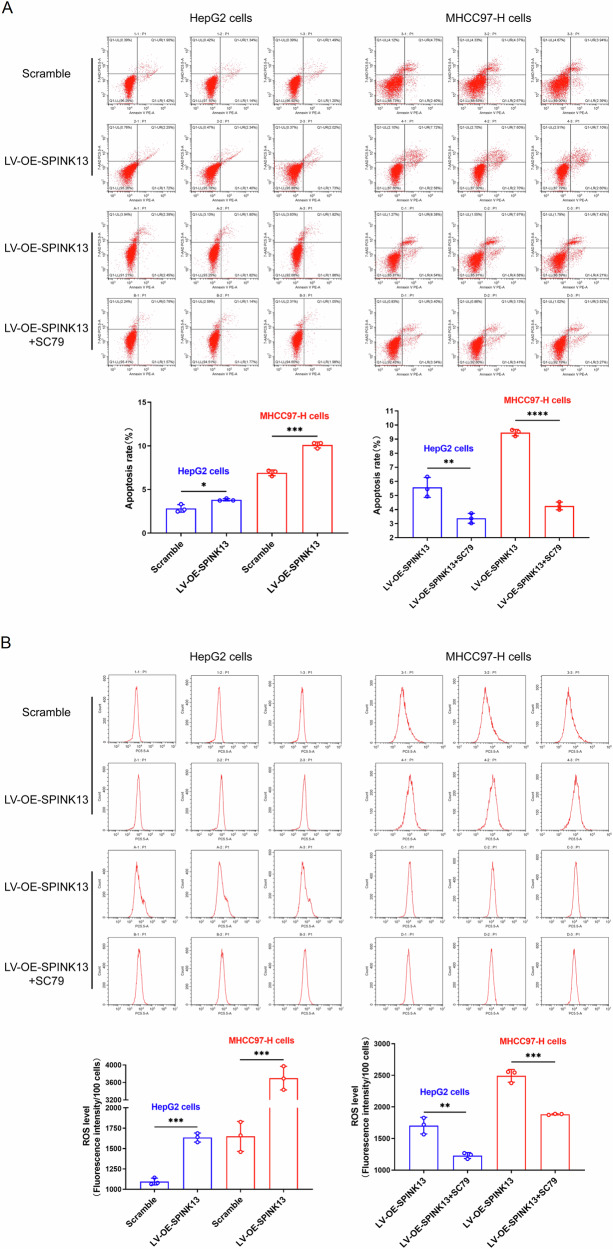
Effects of SPINK13 overexpression on apoptosis and ROS levels. **A** SPINK13 overexpression and its effect on apoptosis in combination with the Akt agonist SC79. Compared with that of the scramble group, the apoptosis rate of the LV-OE-SPINK13 group was significantly greater. *p < 0.05 and ***p < 0.001, *n* = 3. Compared with that of the LV-OE-SPINK13 group, the apoptosis rate of the LV-OE-SPINK13 group treated with 8 μg/ml SC79 was significantly lower. **p < 0.01 and ****p < 0.0001, *n* = 3. **B** SPINK13 overexpression and its effect on cellular ROS levels in combination with the Akt agonist SC79. Compared with that in the scramble group, the ROS level in the LV-OE-SPINK13 group was significantly increased. *p* < 0.001, *n* = 3. Compared with those in the LV-OE-SPINK13 group, the ROS levels in the LV-OE-SPINK13 group treated with 8 μg/ml SC79 were significantly lower, **p < 0.01 and ***p < 0.001, *n* = 3.

**Fig. 3:**
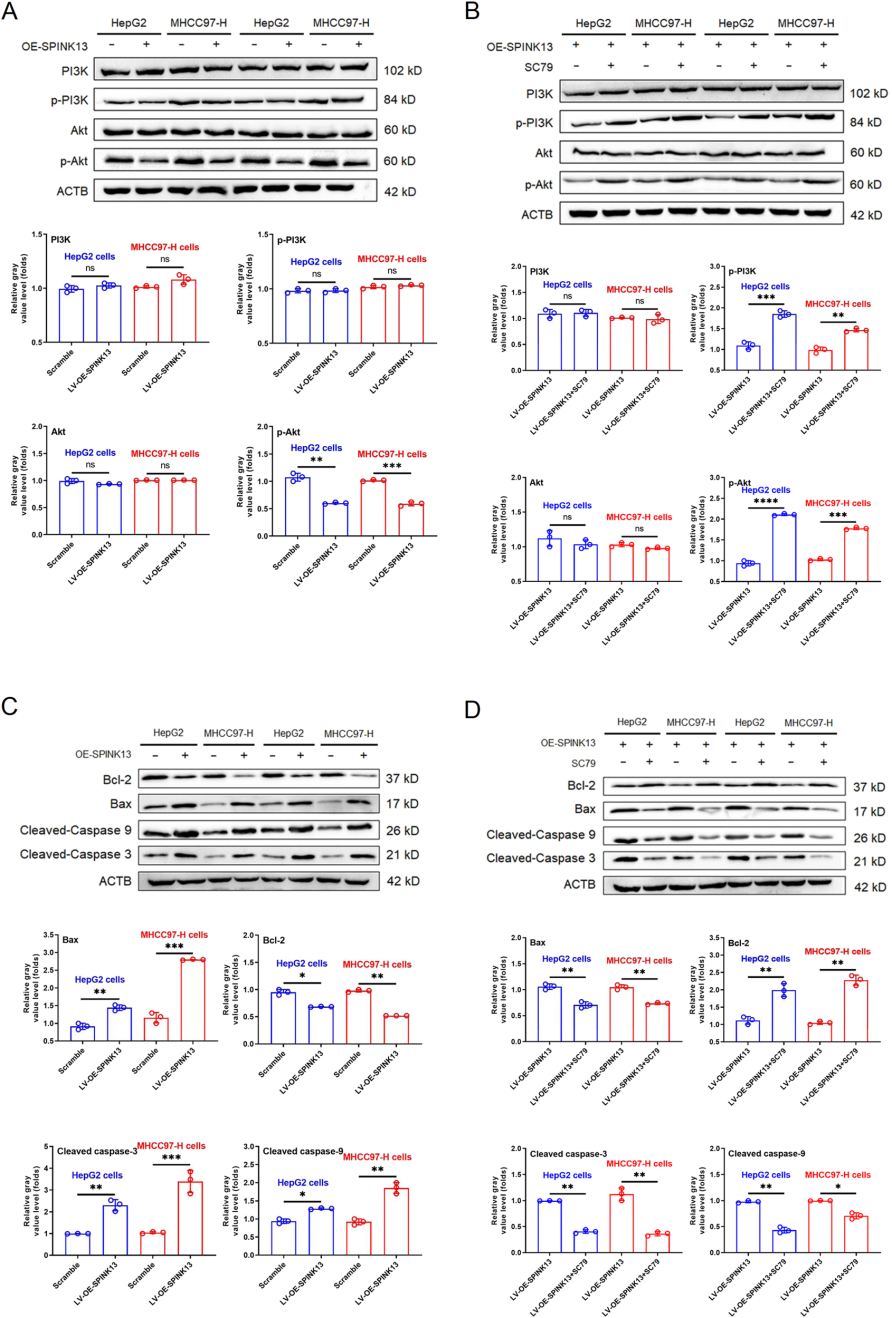
Effects of SPINK13 overexpression on the expression of adapter proteins in the PI3K/Akt pathway and apoptosis regulatory proteins. **A** Effect of SPINK13 overexpression on the expression of adapter proteins in the PI3K/Akt pathway. ns (not significant), **p < 0.01 and ***p < 0.001, the LV-OE-SPINK13 group vs. the scramble group; *n* = 3. **B** Effects of the Akt agonist SC79 on the expression of PI3K/Akt pathway adapter proteins after SPINK13 overexpression. ns (not significant), **p < 0.01, ***p < 0.001 and ****p < 0.0001 for the LV-OE-SPINK13 group with 8 μg/ml SC79 vs. the LV-OE-SPINK13 group, *n* = 3. **C** Effects of SPINK13 overexpression on the expression of apoptosis regulatory proteins. *p < 0.05, **p < 0.01 and ***p < 0.001, the LV-OE-SPINK13 group vs. the scramble group; *n* = 3. **D** Effects of the Akt agonist SC79 on the expression of apoptosis regulatory proteins after SPINK13 overexpression. *p < 0.05 and **p < 0.01, the LV-OE-SPINK13 group with 8 μg/ml SC79 vs. the LV-OE-SPINK13 group, *n* = 3.

Compared with that of the scramble group, the apoptosis rate of the two LV-OE-SPINK13 groups increased by 34.98% (HepG2 cells), and 47.66% (MHCC97-H cells) after SPINK13 was overexpressed, and compared with that of the LV-OE-SPINK13 group, the combination of the Akt agonist SC79 reversed the apoptosis caused by upregulated SPINK13 expression. The apoptosis rate decreased by 31.60% and 45.92%, respectively (Fig. 2A). The level of p-Akt decreased in the LV-OE-SPINK13 group. In contrast, the levels of PI3K, Akt, and p-PI3K remained unchanged (Fig. 3A). Compared with the recombinant lentivirus, the combination of the Akt agonist SC79 reversed the inhibitory effect of SPINK13 on increasing p-Akt levels and increasing p-PI3K levels (Fig. 3B). The upregulation of p-PI3K by p-Akt has rarely been reported. However, there is evidence of feedback loops with PI3K/Akt/mTOR/FOXO1/PI3K [19]. Endogenous apoptotic proteins were detected to clarify the mechanism by which SPINK13 overexpression triggers apoptosis in hepatoma cells. The results revealed that the expression of the pro-apoptotic protein Bax increased, the expression of the antiapoptotic protein Bcl-2 decreased, and the levels of cleaved-caspase 9 and cleaved-caspase-3 in the cytoplasm increased (Fig. 3C). Compared with the LV-OE-SPINK13 group, the combination of the Akt agonist SC79 could also reverse the above levels of apoptosis regulatory proteins (Fig. 3D), suggesting that the PI3K/Akt pathway is inactivated by downregulating p-Akt when SPINK13 is overexpressed and that the mitochondrial apoptosis pathway is activated to induce apoptosis.

ROS can be detected by two fluorescent probes, 2’,7’-dichlorofluorodiacetate (DCFH-DA) and ethidium dihydroide (DHE). However, the excitation and emission of the DCFH-DA fluorescent probes were very similar to those of the GFP probe. Moreover, the stably transfected cell lines emitted green fluorescence from GFP, so the DHE fluorescent probe was selected to detect ROS. The fluorescence intensity reflects the ROS level. The results of flow cytometry revealed that the fluorescence intensity of the LV-OE-SPINK13 group was significantly greater than that of the scramble group (Fig. 2B), indicating a marked increase in ROS levels. Unsurprisingly, the combination of the Akt agonist SC79 reversed the increase in ROS levels compared with those in the LV-OE-SPINK13 group. These data suggest that the mitochondrial apoptotic pathway is one of the pro-apoptotic processes associated with SPINK13 that causes excess ROS.

### Effect of SPINK13 overexpression on the cell cycle of hepatocellular carcinoma cells

**Fig. 4:**
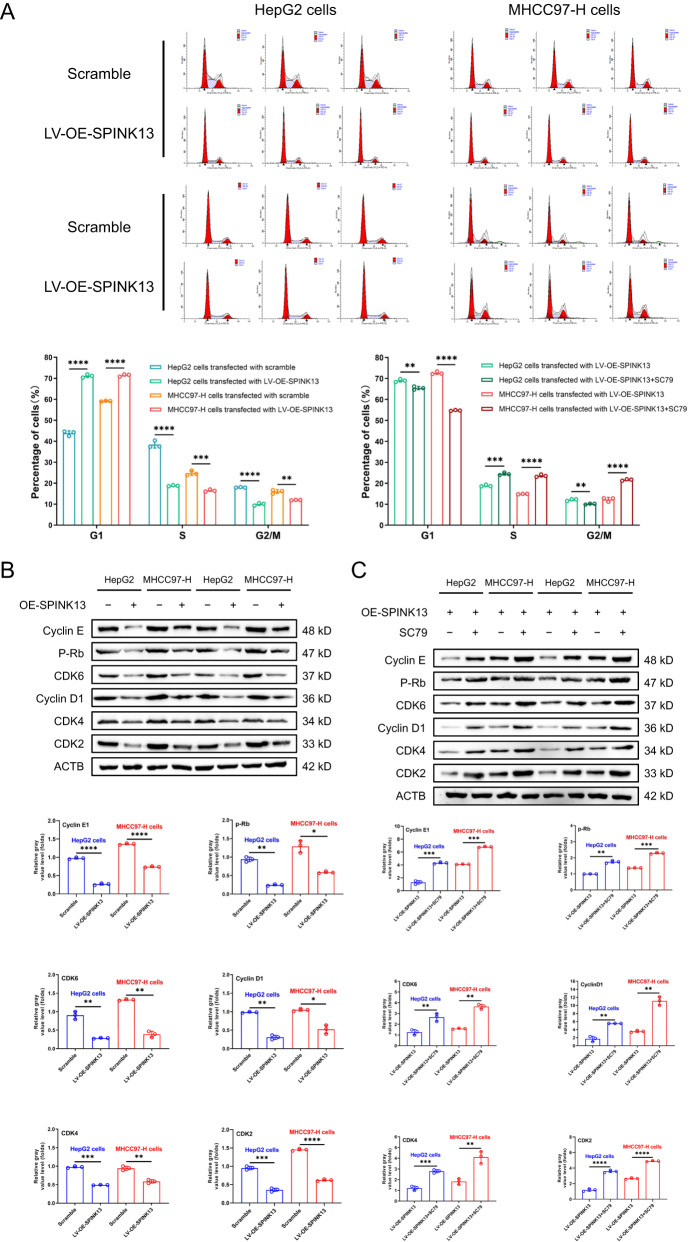
Effects of SPINK13 overexpression on cell cycle arrest. **A** Effects of SPINK13 overexpression alone or in combination with the Akt agonist SC79 on the cell cycle distribution. Compared with that of the scramble group, the cell cycle progression of the LV-OE-SPINK13 group was significantly blocked in the G1/S phase. **p < 0.01, ***p < 0.001 and ****p < 0.0001, *n* = 3. Compared with that in the LV-OE-SPINK13 group, the percentage of cells in the G1/S phase was significantly lower in the LV-OE-SPINK13 group treated with 8 μg/ml SC79. **p < 0.01, ***p < 0.001 and ****p < 0.0001, *n* = 3. **B** Effects of SPINK13 overexpression on the expression of cell cycle regulatory proteins. *p < 0.05, **p < 0.01, ***p < 0.001 and ****p < 0.0001, the LV-OE-SPINK13 group vs. the scramble group; *n* = 3. **C** Effect of the Akt agonist SC79 on cell cycle regulatory protein expression after SPINK13 overexpression. **p < 0.01, ***p < 0.001 and ****p < 0.0001 for the LV-OE-SPINK13 group with 8 μg/ml SC79 vs. the LV-OE-SPINK13 group, *n* = 3.

Compared with that of the scramble group, the proportion of cells in the G1 phase of the two LV-OE-SPINK13 groups tended to increase from 43.18% to 71.13% (HepG2 cells) and from 59.15% to 71.48% (MHCC97-H cells), respectively, when the proportion of both the S and G2/M phases decreased compared with that of the normal cell lines, with statistically significant differences (Fig. 4A). The mechanism of SPINK13 overexpression-induced cell cycle arrest in HCC was analyzed by detecting cell cycle regulatory proteins. Compared with those in the scramble group, the levels of Cyclin D1, Cyclin E1, CDK2, CDK4, CDK6, and p-Rb were significantly lower in the LV-OE-SPINK13 group (Fig. 4B). Coadministration of the Akt agonist SC79 significantly reduced the proportion of cells in the G1 phase and correspondingly increased the proportion of cells in the S phase and G2/M phase in the LV-OE-SPINK13 group (Fig. 4A) but significantly increased the levels of Cyclin D1, Cyclin E1, CDK2, CDK4, CDK6 and p-Rb (Fig. 4C), indicating that overexpression of SPINK13 downregulated the expression or phosphorylation levels of cell cycle regulatory proteins through inactivation of the PI3K/Akt pathway, which in turn induced cell cycle arrest in the G1/S phase.

### Effect of SPINK13 on the tumor formation of hepatocellular carcinoma cells in nude mice in vivo

**Fig. 5:**
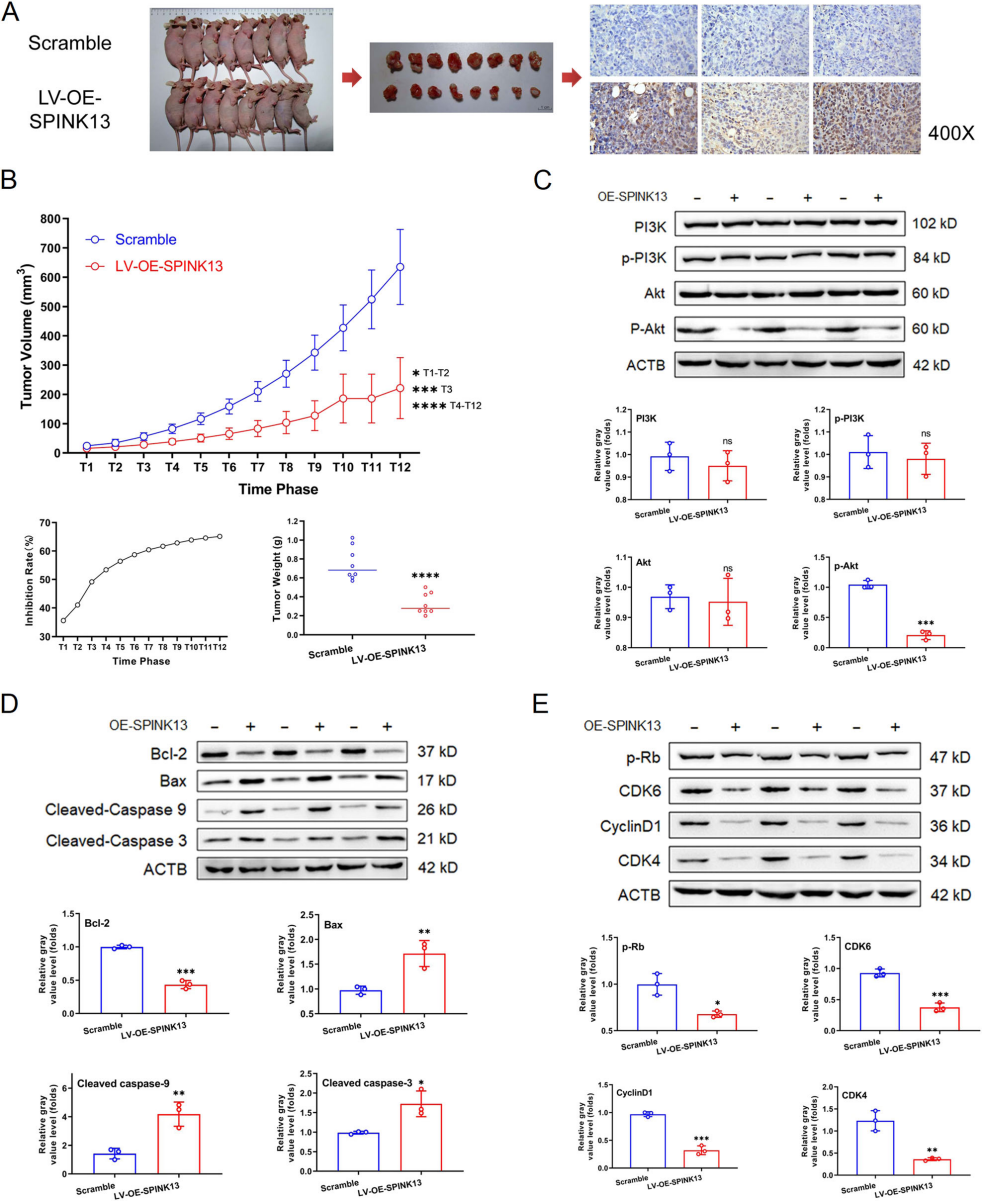
Overexpression of SPINK13 inhibits subcutaneous tumorigenesis in nude mice with hepatocellular carcinoma. MHCC97-H cells stably overexpressing SPINK13 were injected into nude mice to form subcutaneous tumors to establish a xenograft model of human hepatocellular carcinoma. **A** Images of tumors and representative photographs of tumor tissues were obtained via TUNEL staining. **B** Volumes of tumors, inhibition rates from volumes of tumors, and weights of tumors. *p < 0.05, ***p < 0.001 and ****p < 0.0001, *n* = 8. **C** Effect of SPINK13 overexpression on the expression of PI3K/Akt pathway adapter proteins in tumor tissues. ns (not significant), ***p < 0.001 for the LV-OE-SPINK13 group vs. the scramble group, *n* = 3. **D** Effect of SPINK13 overexpression on the expression of apoptosis regulatory proteins in tumor tissues. *p < 0.05, **p < 0.01 and ***p < 0.001, the LV-OE-SPINK13 group vs. the scramble group; *n* = 3. **E** Effect of SPINK13 overexpression on the expression of cell cycle regulatory proteins in tumor tissues. *p < 0.05, **p < 0.01 and ***p < 0.001, the LV-OE-SPINK13 group vs. the scramble group; *n* = 3.

In vitro experiments revealed that SPINK13 induced cell cycle arrest and apoptosis by inactivating the PI3K/Akt pathway intracellularly. To verify the tumor-suppressive effect of SPINK13 in vivo, the effect of SPINK13 overexpression on the subcutaneous tumorigenesis of HCC cells was observed in an MHCC97-H cell xenograft tumor model in nude mice. The results revealed that the rate of subcutaneous tumor formation in the LV-OE-SPINK13 group was significantly lower than that in the scramble group. The difference in tumor volume between the two groups became increasingly significant over time, and the inhibition rate reached 65.16% after 4 weeks. Nude mice were sacrificed after 4 weeks of tumor formation, the tumors were removed and weighed, and the weight of the tumors in the LV-OE-SPINK13 group was significantly lower than that in the scramble group (Fig. 5A, B). TUNEL assay The results revealed that the number of apoptotic tumor cells (brown, dark staining) was significantly greater in the LV-OE-SPINK13 group than in the scramble group, with scores of 3.67 ± 1.70 and 0.07 ± 0.00, respectively, which were significantly different between the two groups (Fig. 5A). In addition, in HCC nude mice with subcutaneously implanted tumors, SPINK13 was similarly able to inhibit the PI3K/Akt pathway by downregulating the level of p-Akt and downregulating the expression or phosphorylation of cell cycle regulatory proteins and activating the mitochondrial apoptotic pathway (Fig. 5C–E).

### Transcriptome sequencing quality assessment and functional enrichment analysis of differentially expressed genes

The quality values of the Q30 in the LV-OE-SPINK13 group and the scramble group reached 94.20% and 93.83%, respectively. The comparison rate of the transplanted tumors with the reference genome in the LV-OE-SPINK13 group was 95.76%, and that of the transplanted tumors with the reference genome in the scramble group was 90.70%. The percentages of multiple mapped reads in the reference sequence were 3.81% and 4.22%, respectively. The sequencing quality is high, and the results are reliable, which meets the requirements of subsequent analysis.

**Fig. 6:**
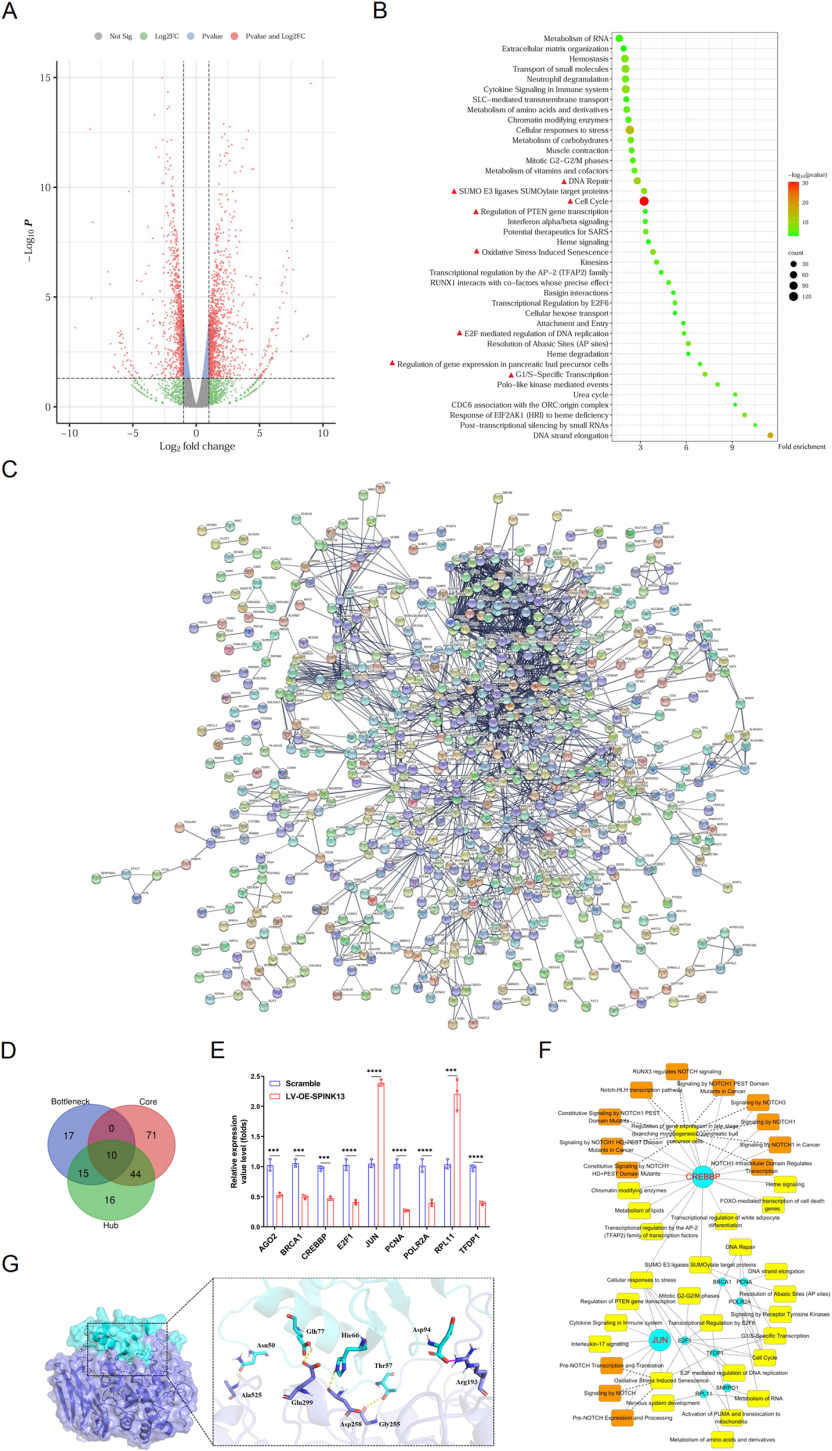
Analysis of the mechanism of action of endogenous SPINK13 via RNA-Seq and molecular docking methods. **A** Significance analysis of differential expression gene sets. A volcano plot was used to visualize genes that were differentially expressed between the LV-OE-SPINK13 group and the scramble group. **B** The results of enrichment analysis of the candidate gene set. The results are shown as images of the top 35 KEGG pathway terms and Reactome pathway terms ranked by *p* value, number of genes, and fold enrichment. **C** Image of the protein-protein interaction network constructed via STRING analysis. **D** Venn analysis results of core genes, hub genes, and bottleneck genes. **E** Verification of the relative expression levels of DEGs related to important pathways (the red triangle in Fig. 5B) via qRT-PCR. **p < 0.01, ***p < 0.001 and ****p < 0.0001, *n* = 3. **F** Key gene-KEGG/Reactome pathway regulatory network constructed via Cytoscape software. The blue ellipses, yellow rectangles, and orange rectangles represent the key genes, KEGG pathways, and Reactome pathways, respectively. **G** SPINK13-Furin molecular docking model. The Furin cartoon model is shown in dark blue, the SPINK13 cartoon model is shown in cyan, and when focusing on the binding region of the two, the binding site is represented by a stick structure of the corresponding color, and the amino acid residue to which it belongs is displayed.

According to the self-defined screening conditions, 1678 DEGs were obtained for improved significance analysis, including 879 upregulated and 799 downregulated genes (Fig. 6A). The candidate gene set involves a wide range of functions. The relevance is reflected in two main functions: cell behavior and transcription regulation (Fig. 6B), including G1/S-specific transcription, oxidative stress-induced senescence, the cell cycle, the regulation of gene expression in pancreatic bud precursor cells, the regulation of PTEN gene transcription, E2F-mediated regulation of DNA replication, the SUMO E3 ligases SUMOylate target proteins, and DNA repair.

### Construction of a key gene-KEGG/Reactome pathway visual regulatory network

The candidate gene set was uploaded to the String online tool and screened according to the set parameter standard (Fig. 6C). After removing duplicate and noninteracting genes, the network was subjected to K-core analysis via the software “MCODE” plug-in, and 125 core genes were screened. The “CentiScape” plug-in of Cytoscape software was used to calculate the topological features of the network and each node. The degree is the total number of edges connected by nodes, and 85 Hub genes were screened. Betweenness is the proportion of the number of all shortest paths passing through this node in the network, and 42 bottleneck genes were screened.

Moreover, those core genes, hub genes, and bottleneck genes, that is, the key genes of SPINK13, are crucial to their network (Tab. S2). Among the key genes, only the JUN gene and the RPL11 gene were upregulated, whereas the other genes were downregulated (Fig. 6D). In the scramble group, the expression levels of 9 genes associated with important pathways were verified, followed by *AGO2*, *CREBBP*, *JUN*, *E2F1*, *PCNA*, *BRCA1*, *RPL11*, *TFDP1* and *POLR2A*. The results showed that the qPCR verification results were consistent with the transcriptome sequencing results (Fig. 6E). The above verified key genes and important pathways obtained by annotation were imported into Cytoscape software to construct a visual regulatory network of key gene-KEGG/Reactome pathways (Fig. 6F).

### Interaction between SPINK13 and Furin

**Fig. 7:**
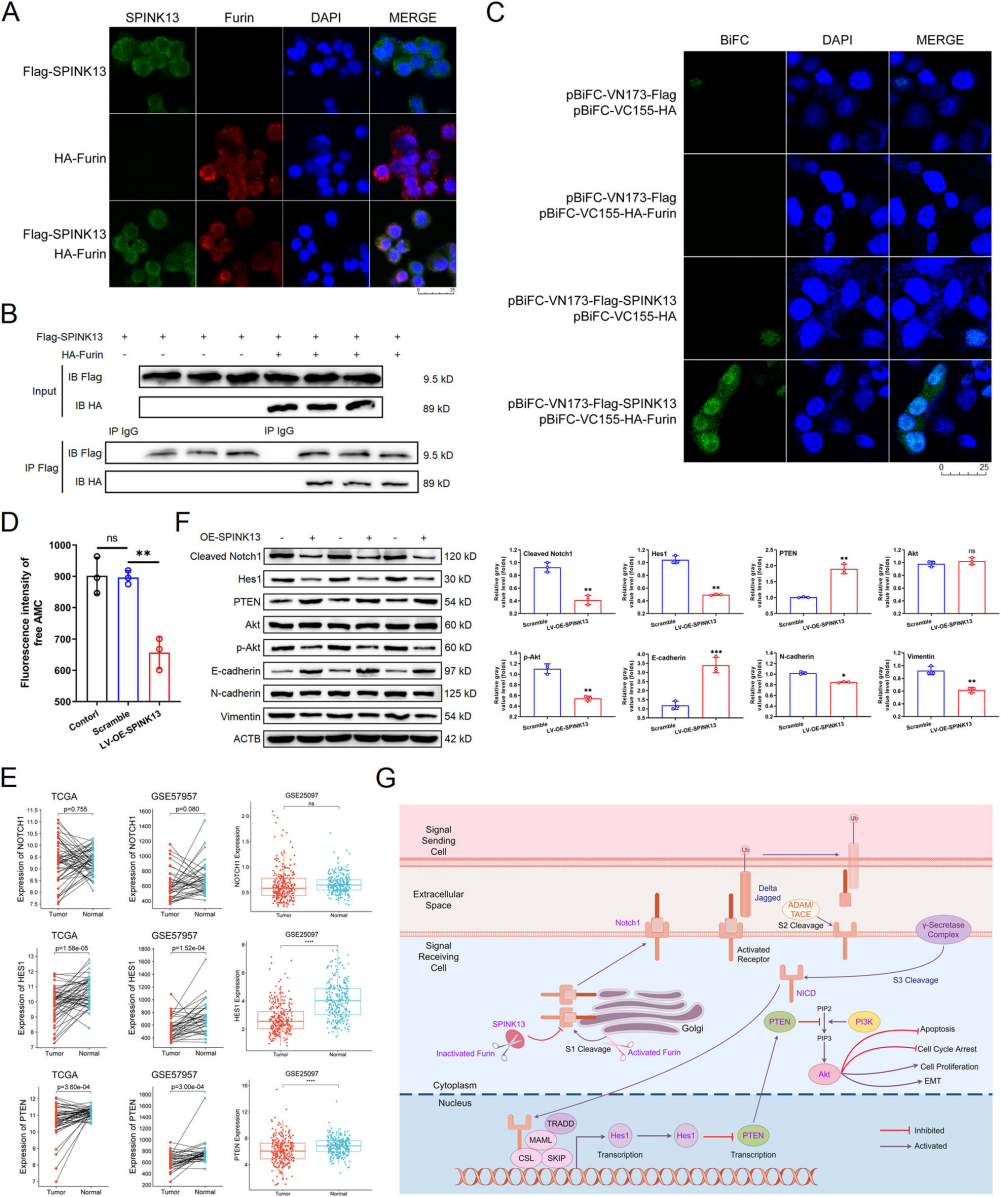
SPINK13 downregulates the Notch1/Hes1 pathway by interacting with Furin. **A** Colocalization of SPINK13 (in green) and Furin (in red) in MHCC97-H cells. The nuclei were stained blue with DAPI. Scale bars = 25 μm. **B** Verification of the interaction between SPINK13 and Furin via a coimmunoprecipitation assay. Flag-SPINK13 and HA-Furin expression was detected by western blot following Flag pull-down from cell lysates. **C** BiFC assays to evaluate interactions between SPINK13 and Furin. In the BiFC system, interacting proteins are shown in green, and the nuclei are stained blue with DAPI. Scale bars = 25 μm. **D** Effect of SPINK13 overexpression on Furin enzyme activity. ns (not significant) represents the scramble group vs. the control group, *n* = 3; **p < 0.01 represents the LV-OE-SPINK13 group vs. the scramble group, *n* = 3. **E** Analysis of the expression of the Notch1/Hes1/PTEN pathway in HCC tissues on the basis of the database. In the TCGA database and GSE57957 datasets, the tumor group and the normal group were liver cancer and adjacent tissues, respectively (*n* = 50 and *n* = 39). In the GSE25097 dataset, the tumor group and the normal group were liver cancer tissues (*n* = 268) and normal tissues (*n* = 243), respectively. ns (not significant) and ****p < 0.0001 for the tumor group vs. the normal group. **F** Effects of SPINK13 overexpression on the expression of Notch1/Hes1 pathway adapter proteins and downstream EMT-related proteins in tumor tissue. ns (not significant), **p < 0.01 and ***p < 0.001, the LV-OE-SPINK13 group vs. the scramble group; *n* = 3. **G** Schematic diagram of the mechanism of action of endogenous SPINK13. Graphical depiction of how endogenous SPINK13 inhibits Furin protease activity by binding triggers the Notch1/Hes1-PTEN/Akt signaling axis to induce mitochondrial apoptosis and cell cycle arrest, and reverses EMT. The schematic was drawn by Figdraw.

The molecular docking simulation results revealed that multiple sets of residues are used to form hydrogen bonds between Furin and SPINK13, such as the hydrogen bond formed by Furin Gly255 and SPINK13 Thr57 (Fig. 6G). Under these interacting forces, the Furin–SPINK13 docking model was calculated to score −427, which is a better performance. Immunofluorescence imaging revealed that SPINK13 proteins with green fluorescence were distributed in the cytoplasm and were able to colocalize with Furin proteins with red tags to emit yellow fluorescence, suggesting that SPINK13 colocalized with Furin subcellularly in the cytoplasm (Fig. 7A). MHCC97-H cells were cotransfected with recombinant plasmids of Flag-tagged SPINK13 and HA-tagged Furin, and the immunocomplexes were immunoprecipitated with Flag antibody-coupled magnetic beads in the lysates of the cells that coexpressed SPINK13 and Furin, and the Flag antibody and HA antibody were utilized to detect their respective counterparts of Flag-SPINK13 and HA-Furin, demonstrating that SPINK13 interacts with Furin (Fig. 7B). To further verify the interaction between SPINK13 and Furin proteins, MHCC97-H cells were cotransfected with a recombinant plasmid expressing an N-terminal green fluorescent protein (SPINK13) fusion protein and a Furin fusion protein expressing a C-terminal green fluorescent protein. As a result, the green fluorescence of the cotransfected cells was distributed in the cytoplasm, while the cells expressing only the SPINK13 fusion protein presented minimal green fluorescence. The cells expressing only the Furin fusion protein barely emitted green fluorescence, thus confirming the interaction between SPINK13 and Furin (Fig. 7C). Further analysis revealed that SPINK13 significantly inhibited Furin enzyme activity. Compared with that in both the normal control group and the scramble group, the Furin enzyme activity in the LV-OE-SPINK13 group decreased by 27.21% and 26.74%, respectively (Fig. 7D).

### Effects of SPINK13 on the expression of Notch1/Hes1 pathway junction proteins and downstream EMT-related proteins in tumor tissues

Furin can act as an upstream molecule to cleave immature Notch1 in the trans-Golgi to form an activated heterodimer, which further cleaves to form the Notch intracellular domain (NICD), thereby regulating the downstream Hes1/PTEN/Akt pathway. To further validate the regulatory role of SPINK13 in the Notch1/Hes1/PTEN pathway, it is essential to examine its expression levels in HCC tissues. We obtained an mRNA expression matrix comprising 50 pairs of HCC tissues and their adjacent normal tissue samples from the TCGA database. The analysis revealed that the expression level of Notch1 in HCC tissues did not significantly differ from that in adjacent tissues; however, the expression levels of both Hes1 and PTEN markedly decreased. Additionally, we downloaded two human HCC expression profile datasets from the GEO database: GSE25097, which includes 268 HCC tissues and 243 normal tissues, and GSE57957, which consists of 39 paired samples of HCC tissues alongside adjacent normal tissues (Fig. 7E). In this study, the western blot results revealed that SPINK13 downregulated Hes1 levels while causing a decrease in cleaved Notch1 levels in tumor tissues and consequently downregulated the activation of PTEN expression, which in turn inhibited Akt phosphorylation (Fig. 7F), which differs from the results of the data analysis. In addition, SPINK13 was able to upregulate the expression of the mesenchymal cell markers N-cadherin and vimentin while significantly downregulating the expression of the epithelial cell marker E-cadherin (Fig. 7F). Combined with its ability to stabilize p-PI3K levels and reduce p-Akt levels, SPINK13 might downregulate the Notch1/Hes1/PTEN/Akt signaling pathway through the inhibition of Furin activity, thereby reversing the epithelial-mesenchymal transition (EMT) state of HCC cells and inducing apoptosis and cell cycle arrest (Fig. 7G).

## Discussion

Liver cancer is the sixth most common cancer in the world and the fourth leading cause of cancer death. Primary liver cancer is represented by intrahepatic cholangiocarcinoma and hepatocellular carcinoma, and HCC accounts for approximately 75–85% of cases. It generally lacks effective early diagnosis and prevention methods and has an insidious onset and rapid progression of the disease [20]. As a highly aggressive tumor, HCC has a very high recurrence and migration rate, and the overall prognosis is poor [21]. The overall prognosis is poor. The treatment options for early- to intermediate-stage HCC are relatively stable and include surgical resection, anhydrous ethanol injection (PEI), radiofrequency ablation (RFA), transarterial chemoembolization (TACE), and liver transplantation. However, these methods are only applicable to a small number of patients, especially those without cirrhosis, and are often associated with complications [22].

Sorafenib, as the first-line treatment for advanced liver cancer, benefits fewer than one-third of patients, with a median survival prolonged by only 3 months, and drug resistance and related complications unavoidably occur after 6 months of drug use [23, 24]. The reason for this is that sorafenib has been shown to be the first-line treatment for liver cancer. The reason is that sorafenib inhibits the proliferation of HCC cells and neovascularization by inactivating the Ras/Raf/MEK/ERK pathway but simultaneously activates the PI3K/Akt pathway and inhibits the expression of the tumor suppressor phosphatidylinositol esterase and tension protein homolog (PTEN). PTEN is the second most frequently mutated and deleted tumor suppressor gene, followed by p53. Its deletion, mutation, or inactivation is prevalent in the development and progression of various tumor types and has been associated with tumor invasion and migration [25]. According to the literature, the expression level of PTEN in HCC tissues is significantly lower than that observed in adjacent tissues and normal liver tissues. This reduction can serve as a risk factor for predicting the prognosis of patients with HCC [26, 27]. The findings from both the clinical cohort studies and this study align with these expectations. PTEN dephosphorylates PIP3 to generate PIP2, thereby inactivating PI3K and antagonizing the effects of Akt, leading to the development of HCC cells with a high degree of resistance. The Akt effect leads to acquired resistance to sorafenib in HCC cells [28]. In fact, in 40–50% of HCC patients, mutation or downregulation of the PTEN protein can be observed, resulting in intracellular PIP3 accumulation and continuous activation of the PI3K/Akt pathway, which promotes the expression of antiapoptotic genes and inhibits apoptosis, resulting in an imbalance between the rates of cell proliferation and apoptosis [29]. The result is an imbalance between the rates of cell proliferation and apoptosis. Considering the rapid increase in the incidence of HCC worldwide and the limited choice of actual therapeutic options, the development of novel targeted PI3K/Akt pathway drugs with low dosages, weak toxicity, and significant therapeutic effects is not only the focus of current clinical practice but also the focus of future therapeutic development.

The inactivated PI3k/Akt pathway plays an important role in endogenous SPINK13-induced mitochondrial apoptosis and cell cycle arrest in HCC cell lines stably overexpressing SPINK13. Numerous studies have shown that the PI3K/Akt pathway is overexpressed in nearly half of HCC and that dysregulated expression of this pathway has a wide range of effects, including effects on cell proliferation, the cell cycle, metabolism, differentiation, autophagy, angiogenesis, and EMT [30]. As one of the major intracellular signaling pathways, the PI3K/Akt pathway is activated in a variety of cancers via the action of receptor tyrosine kinase (RTK). Activated PI3K then phosphorylates phosphatidylinositol-4,5-bisphosphate (PIP2) to produce phosphatidylinositol-3,4,5-trisphosphate (PIP3), which interacts with phosphatidylinositol-3-phosphate-dependent protein kinase 1 (PDK1) and Akt to recruit them to the plasma membrane, where PDK1 phosphorylates Akt Thr308 to activate Akt [31, 32]. Protein kinase B (PKB) is a serine/threonine-specific protein kinase with an essential role in various cellular mechanisms. p-Akt remains in the cytoplasm or translocates to the nucleus to target and stimulate a number of downstream proteins, including the pro-apoptotic factors Bad at the Ser136 and Bax at the Ser184 sites.

On the one hand, the phosphorylation of Bad promotes the binding of Bad to the antiapoptotic protein 14-3-3 so that Bad is localized in the cytoplasm and cannot enter the mitochondria to form heterodimers with Bcl-XL or Bcl-2; on the other hand, it promotes the formation of heterodimers between Bax and Bcl-XL or Bcl-2, prevents the formation of homodimers with Bax, and prevents the restoration of the antiapoptotic function of Bcl-XL or Bcl-2, which ultimately leads to the restoration of the antiapoptotic function of Bax. This antiapoptotic function ultimately leads to mitochondrial apoptosis [33, 34]. In a nude mouse xenograft tumor model of HCC, endogenous SPINK13 had a significant tumor-suppressive effect, and its mechanism of action was consistent with the results of in vitro experiments. Although more drug experimental protocols are needed to confirm this, notably, no significant toxicity from SPINK13 was observed in animals. In addition, ROS generated as a result of mitochondrial apoptosis have two sides to the pathway in terms of promoting tumor cell survival or apoptotic mechanisms, which depend on the level of ROS and the duration of exposure [35]. Low levels of ROS act as mitogens to induce cell proliferation; moderate levels of ROS induce temporary or permanent cell cycle arrest and promote cell differentiation; and high levels of ROS destroy biomolecules, leading to apoptosis [36]. High levels of ROS can destroy biomolecules, leading to apoptosis. Resistance to endogenous ROS elevation is an important feature of tumor cells due to metabolic abnormalities and mitochondrial dysfunction, which limits their ability to cope with the additional increase in ROS and increases their vulnerability to oxidative stress injury [37, 38].

The fate of tumor cells often depends on the ability of certain interactions to amplify and solidify molecular differences between cells and their eventual outcome by regulating cell proliferation, invasion, and apoptosis, among other processes, as well as physiological processes such as angiogenesis [39]. Serpins have a paradoxical manifestation of inhibition and promotion in tumors due to the type and nature of the substrate of action. In addition to the direct interaction of Serpins with upstream and downstream substrates, the interaction between different substrates with different functions and other proteins affects the biological processes of tumor cells [40]. To elucidate the intracellular regulatory mechanism of SPINK13, tumor tissue transcriptome analysis was performed, and the results were experimentally verified. The results revealed that the target genes regulated by SPINK13 were involved mainly in the Notch1 signaling pathway, which has an obvious upstream/downstream relationship with the PI3K/Akt pathway. Notch1, a type I transmembrane receptor protein, is elevated in a variety of tumors and is associated with a poor prognosis [41, 42]. Notch1 is synthesized in the endoplasmic reticulum and transported to the trans-Golgi, where it is cleaved by Furin protein convertase at the Notch1 S1 site (Arg1654-Glu1655 in the extracellular region), generating the Notch extracellular domain (NECD) and transmembrane fragment (NTM). NECD subunits and NTM subunits form a heterodimer, which is then transported to the cell membrane. When the ligand binds to the Notch1 receptor outside the cell membrane, it is cleaved by TNF α-converting enzyme or Kuzbanian enzyme at the S2 site of the heterodimer (Ala1710--Val1711 in the extracellular near-membrane region) to generate N-terminal and C-terminal hydrolysis products. The extracellular N-terminal hydrolysis product is phagocytosed by ligand-expressing cells, whereas the C-terminal hydrolysis product is hydrolyzed by the γ-secretase complex in the transmembrane region. The C-terminal hydrolysis products are hydrolyzed by the γ-secretase complex in the transmembrane region to generate the NICD and translocate into the nucleus to form a transcriptional activation complex, which then mediates the transcription of its downstream target gene, Hes1, which significantly reduces its promoter activity by binding to the PTEN promoter, thus downregulating PTEN transcription and subsequently activating the downstream PTEN/Akt pathway [41].

Elevated levels of Notch1 have been identified in various tumors and are correlated with poor prognostic outcomes. However, the role of Notch1 is paradoxical and varies depending on the specific tissue type [41, 42]. According to the literature, the expression level of Notch1 in HCC tissue is significantly greater than that in adjacent liver tissue and normal liver tissue [43–45]. As Hes1 is a downstream target gene of the Notch1 signaling pathway, its expression is closely associated with Notch1 activation. Notably, Hes1 expression is markedly increased in HCC tissues, whereas Hes1 expression in adjacent tissues is comparatively low, suggesting that Hes1 may play a role in the malignant transformation of HCC [45].

Nevertheless, the relationship between Notch1 expression levels and prognosis in patients with HCC remains contentious, likely owing to limitations in sample sizes or heterogeneity among studies [46]. Clinical cohort studies have indicated that Notch1 expression levels in HCC tissues are closely linked to tumor differentiation grade, metastasis, venous invasion, and tumor node metastasis (TNM) stage, with expression levels decreasing as the pathological grade of HCC increases [47, 48]. Consequently, the expression levels of Notch1 and Hes1 in HCC tissues do not consistently align across all levels, which may account for the discrepancies between the findings of clinical cohort studies and the results of this study. Elevated expression of Notch1 and Hes1 in HCC tissues is associated with malignant characteristics of the tumor, indicating a poor prognosis for patients, whereas reduced expression of PTEN may promote the occurrence and progression of HCC [48]. The PTEN/Akt pathway is then activated downstream. Among the multilevel regulatory factors, the binding of Furin to Notch1 and the generation of mature Notch1 fragments are the most critical regulatory steps in the Notch1 signaling pathway [49]. During apoptosis, inactivation of the Notch1/Hes1 pathway can cause activation and phosphorylation of the downstream substrate PTEN, which inhibits Akt phosphorylation by dephosphorylating PIP3 to PIP2 [50]. PTEN inhibits Akt phosphorylation by dephosphorylating PIP3 to PIP2. In this study, we provide preliminary evidence that endogenous SPINK13 interacts with Furin through subcellular colocalization, immunoprecipitation, bimolecular immunofluorescence complementation, and enzyme activity inhibition assays, thereby inhibiting Furin enzyme activity.

Since Furin belongs to the precursor protein convertase family of the serine protease superfamily, serpins can irreversibly inhibit the activities of different serine proteases to regulate various physiological processes, e.g., serpin B8 and PAI-1, and can bind to and inhibit the activity of Furin [51, 52]. Therefore, SPINK13 may initially prevent or diminish the cleavage of Notch1 by interacting with Furin and inhibiting its activity, subsequently causing downregulation of Hes1 expression, upregulation of PTEN expression, and inactivation of the PI3K/Akt pathway, which mediate the mitochondrial apoptosis and cell cycle arrest in HCC cells triggered by SPINK13, as well as the inhibition of EMT. EMT, as an initial step in the invasive migration of tumor cells, is a biological process in which epithelial cells are transformed into cells with a mesenchymal phenotype [53]. A variety of growth factors, cytokines, and signaling pathways can be involved in the EMT process. Tumor cells exhibit decreased E-cadherin function, upregulated expression of N-cadherin and vimentin, and abnormally high expression of EMT-associated transcription factors, the latter of which downregulate E-cadherin transcription to promote EMT, the PI3K/Akt pathway is a common signaling pathway that induces EMT [54]. Growth factors or cytokines activate the PI3K/Akt pathway to promote EMT by binding to their cognate receptor tyrosine kinases (RTKs), cytokine receptors, or G protein-coupled receptors (GPCRs) [55, 56].

Indeed, the primary objective of this study was to demonstrate that SPINK13 exerts antitumor effects by inhibiting the PI3K/Akt pathway, and on the basis of these findings, we aimed to elucidate these mechanisms by investigating the targets of SPINK13 and their associated upstream crosstalk pathways. However, certain findings presented in this study are questionable. First, more studies are needed to rely on forward experiments to confirm the interaction between SPINK13 and Furin; therefore, it is imperative to design rescue experiments at both the cellular and animal levels for further validation. Additionally, the binding site of the interaction between SPINK13 and Furin, as well as the mechanism by which SPINK13 inhibits the downstream Notch1 pathway, requires clarification. Second, a multitude of downstream substrates of Akt, including FoxO, mTOR, MDM2, and NF-κB, influence the survival, proliferation, and metabolism of tumor cells through their downstream effectors, ultimately determining cell fate [30, 57]. Consequently, elucidating the downstream biological effects and mechanisms induced by SPINK13 in this context is essential.

The Notch1/Hes1/PTEN pathway is the upstream mechanism of action of SPINK13 in the intracellular downregulation of the PI3K/Akt pathway. SPINK13 induces mitochondrial apoptosis and cell cycle arrest by triggering the Notch1/Hes1-PTEN/Akt signaling axis and reversing EMT, thereby inhibiting HCC cell nude mouse xenograft tumor subcutaneous growth. Although these findings form a hierarchical model of endogenous SPINK13-induced apoptosis in HCC cells, the inhibition of Furin activity by SPINK13 is a key event leading to PTEN activation and inactivation of the PI3K/Akt pathway. These findings help to elucidate the mechanism of the anti-HCC action of SPINK13 and, at the same time, lay the theoretical foundation for the development of therapeutic serine protease inhibitors.

## Supplementary information

aj-checklist

Original WB

Supplementary Tables

## Data Availability

The raw RNA-sequencing data (accession number: CRA015274) reported in this paper have been deposited in the National Genomics Data Center of China (https://ngdc.cncb.ac.cn). All datasets analyzed in the study are available from the corresponding authors upon reasonable request.
