# Supplementary material for: SPINK13 acts as a tumor suppressor in hepatocellular carcinoma by inhibiting Akt phosphorylation
Source: Cell Death Dis. 2024 Nov 13;15(11):822. doi: 10.1038/s41419-024-07214-3 (PMC11561306; doi:10.1038/s41419-024-07214-3)

Fig. 1C SPINK13


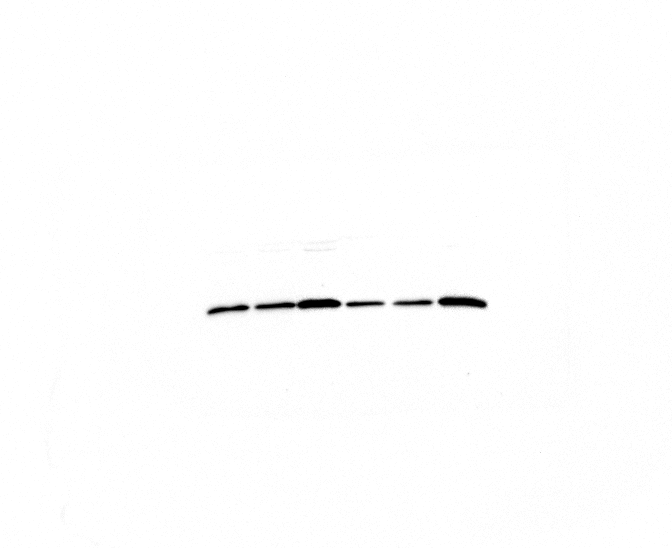


Fig. 1C β-actin


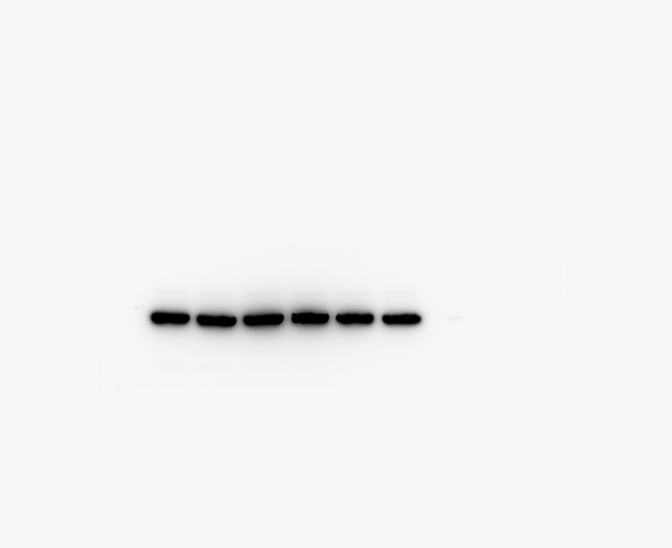


Fig. 3B Akt


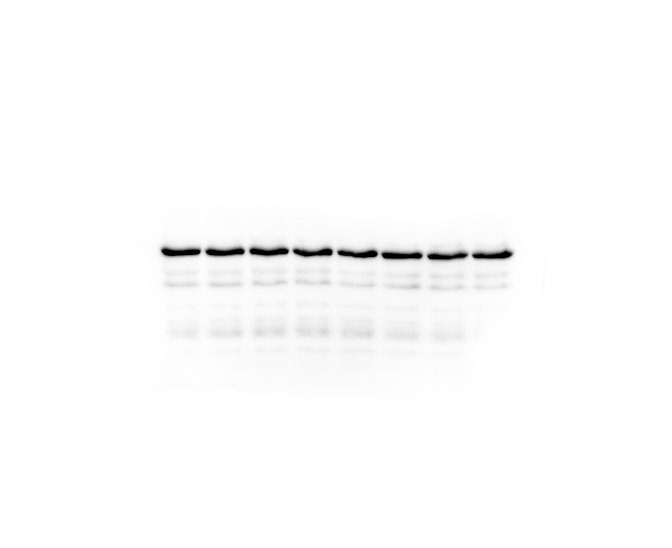


Fig. 3B P-Akt


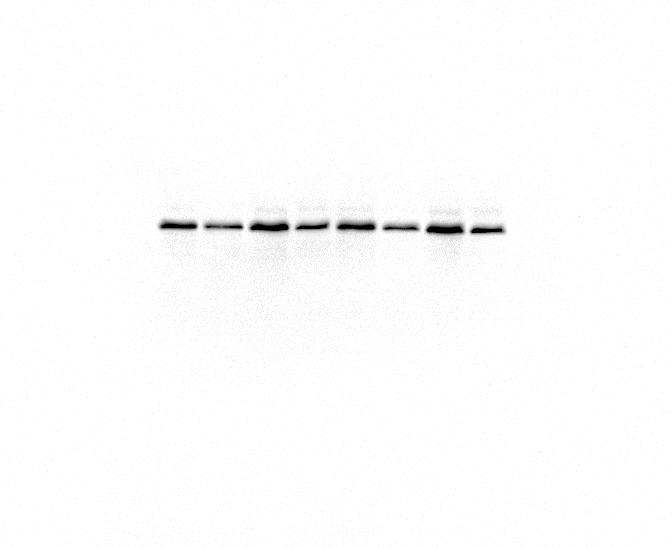


Fig. 3B PI3K


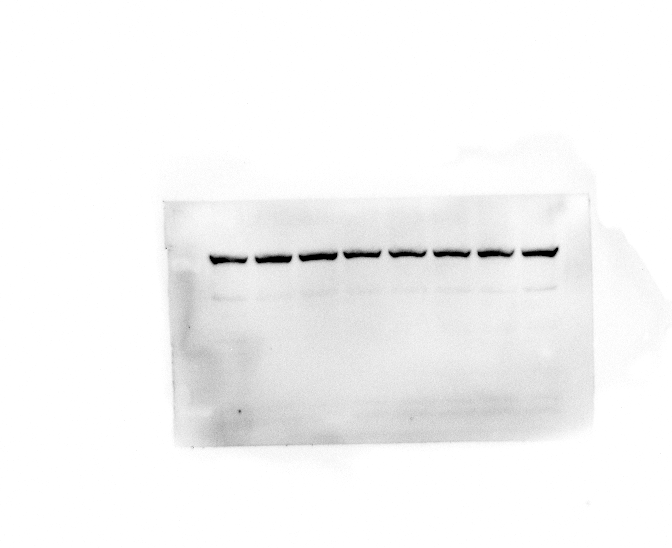


Fig. 3B P-PI3K


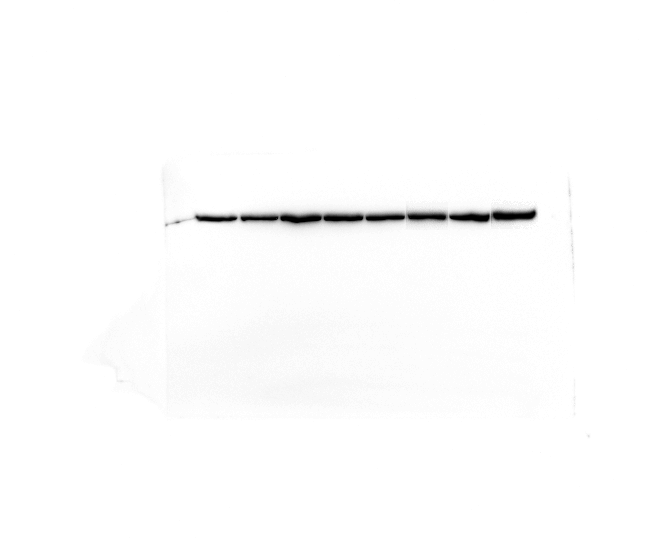


Fig. 3B β-actin


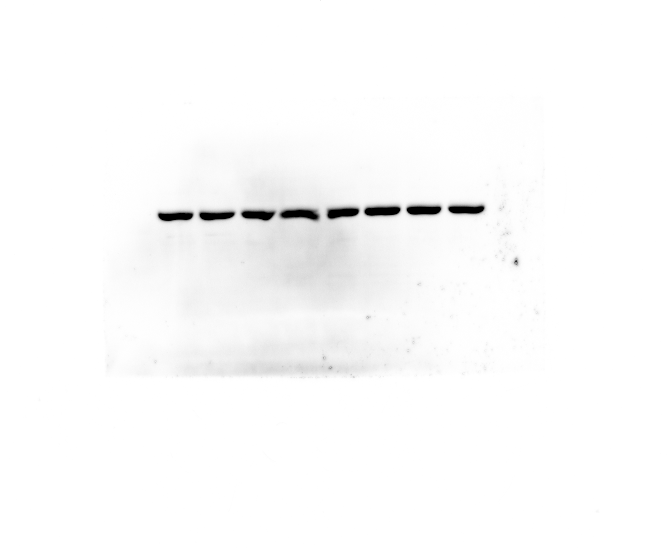


Fig. 3C Akt


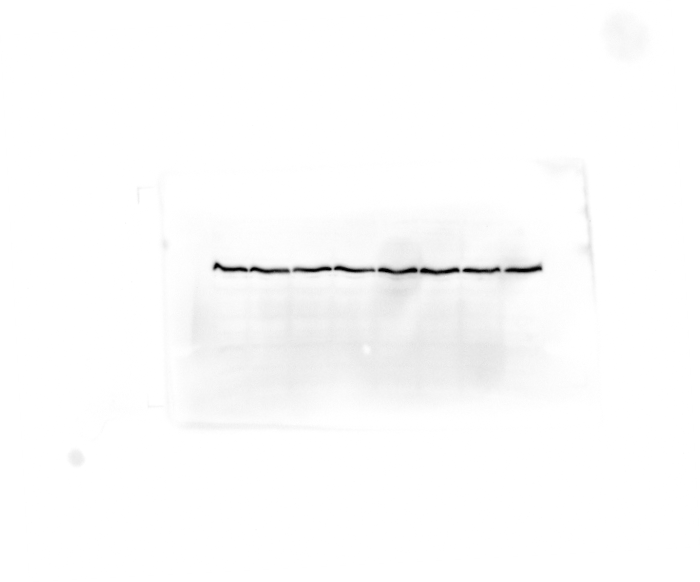


Fig. 3C P-Akt


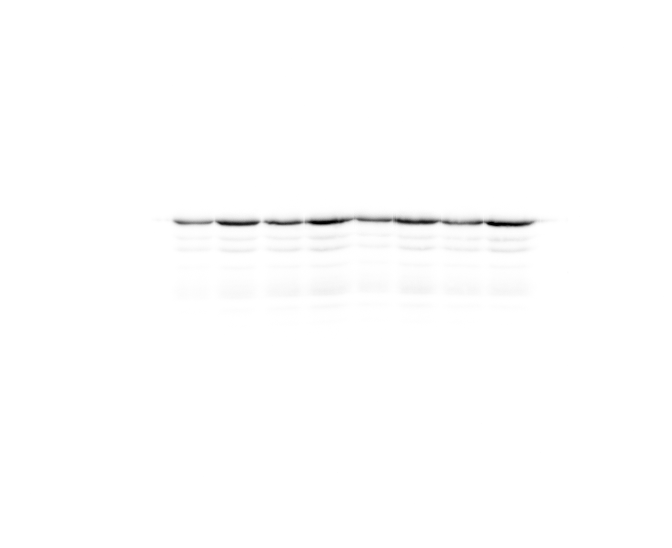


Fig. 3C PI3K


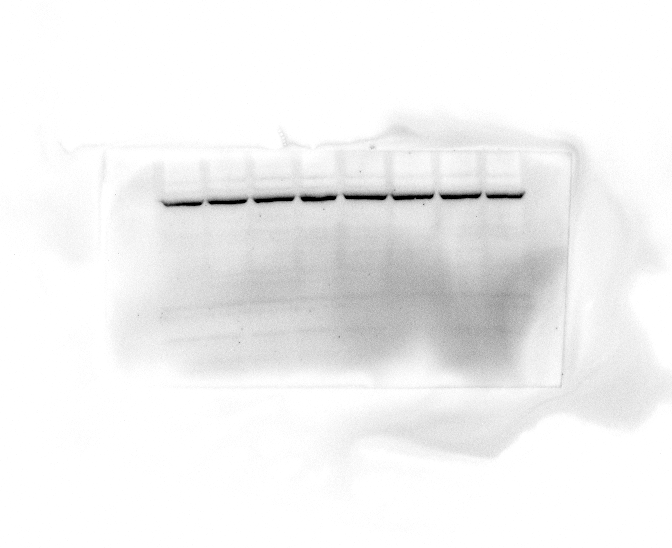


Fig. 3C P-PI3K


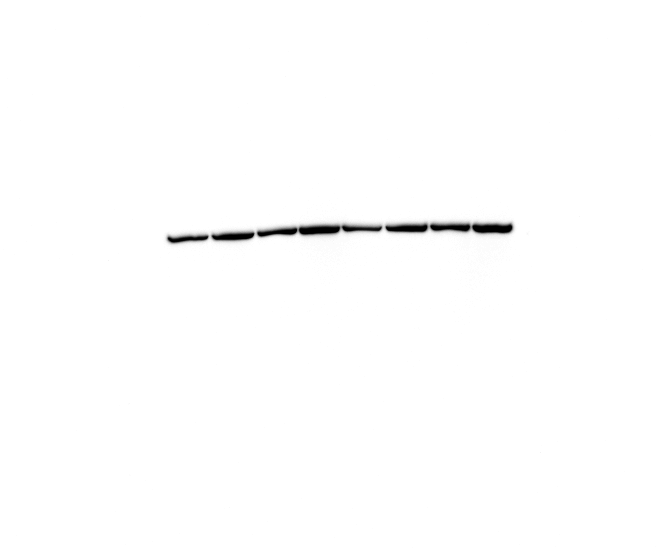


Fig. 3C β-actin


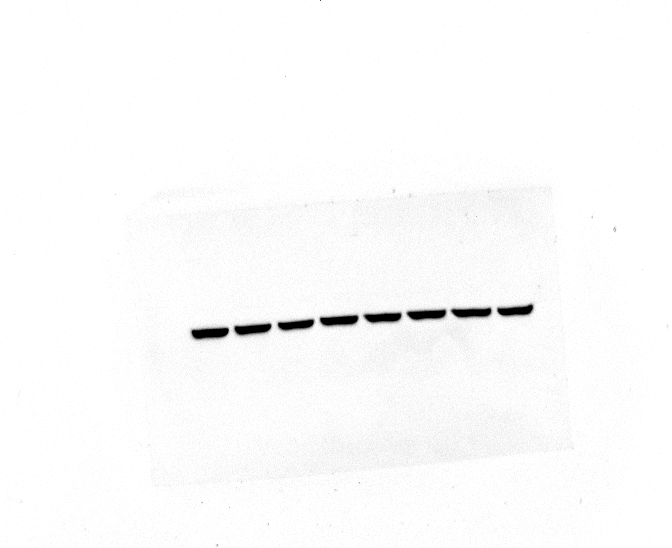


Fig. 3D Bax


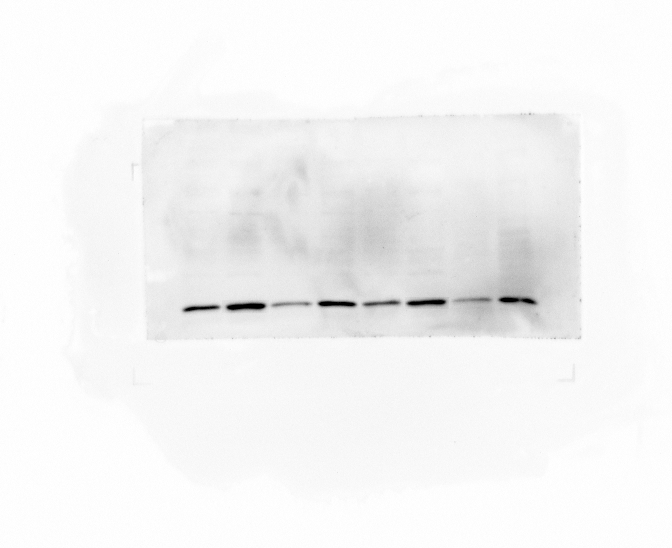


Fig. 3D Bcl-2


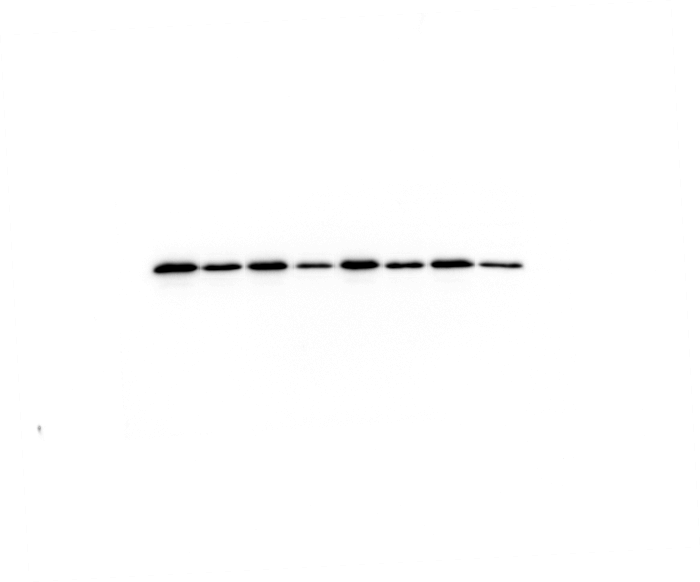


Fig. 3D Cleaved Caspase-3


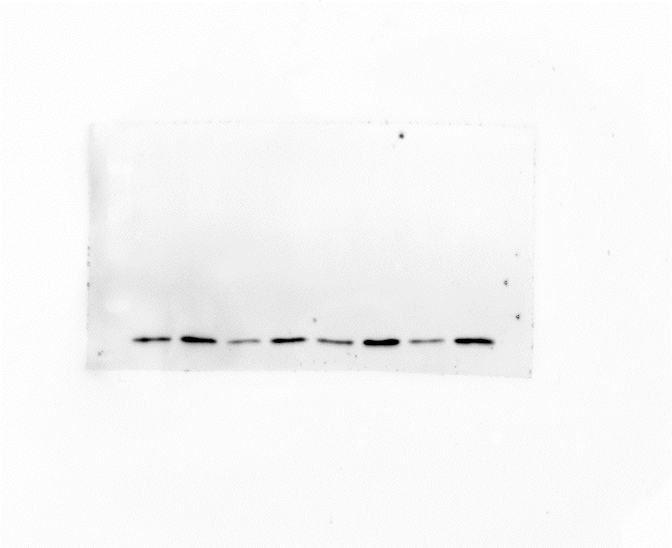


Fig. 3D Cleaved Caspase-9


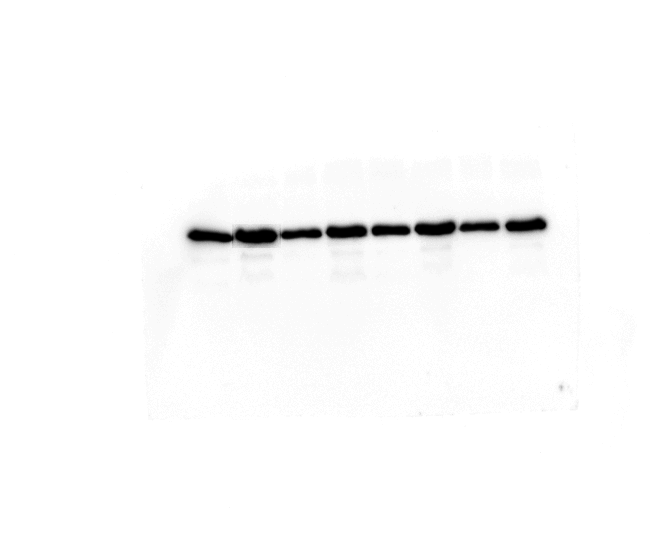


Fig. 3D β-actin


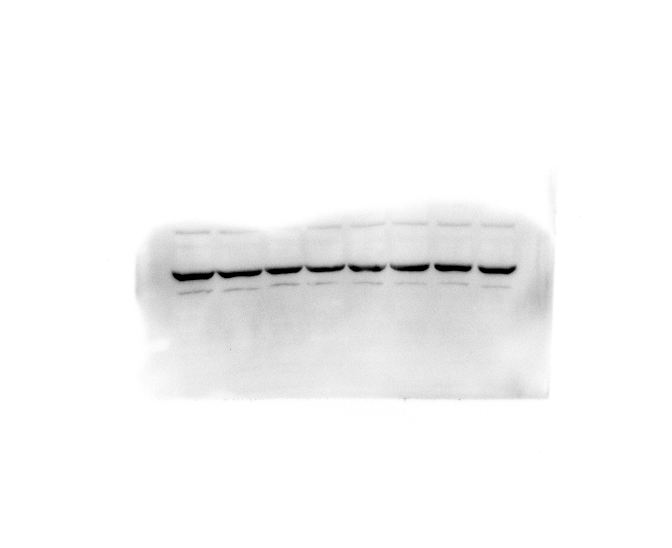


Fig. 3E Bax


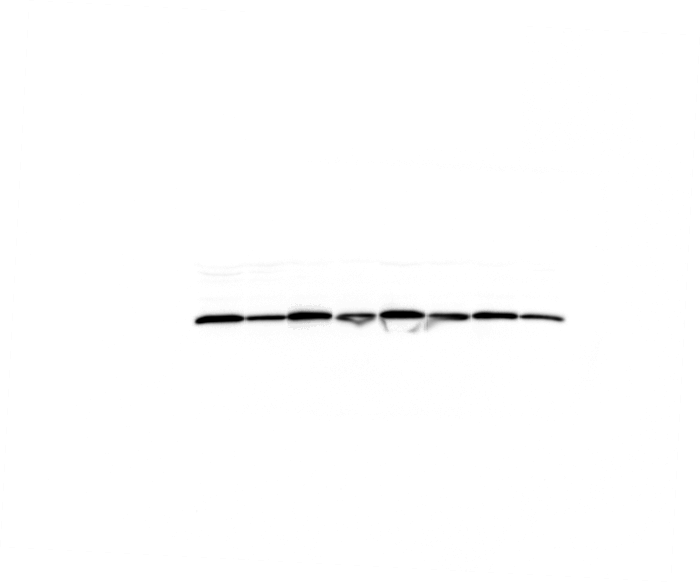


Fig. 3E Bcl-2


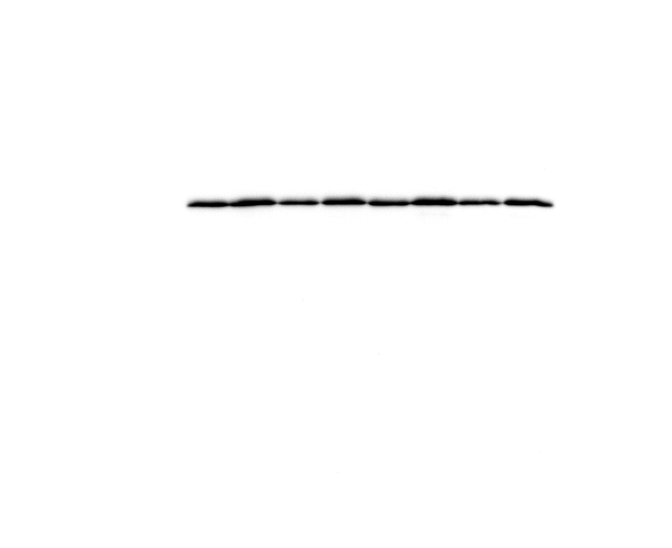


Fig. 3E Cleaved Caspase-3


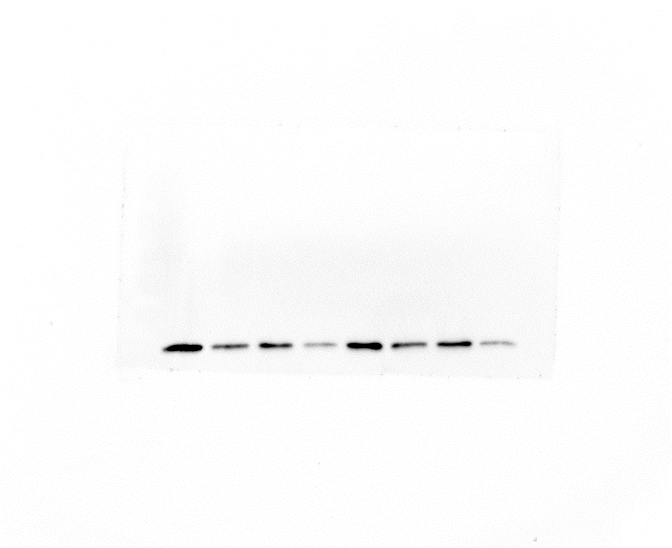


Fig. 3E Cleaved Caspase-9


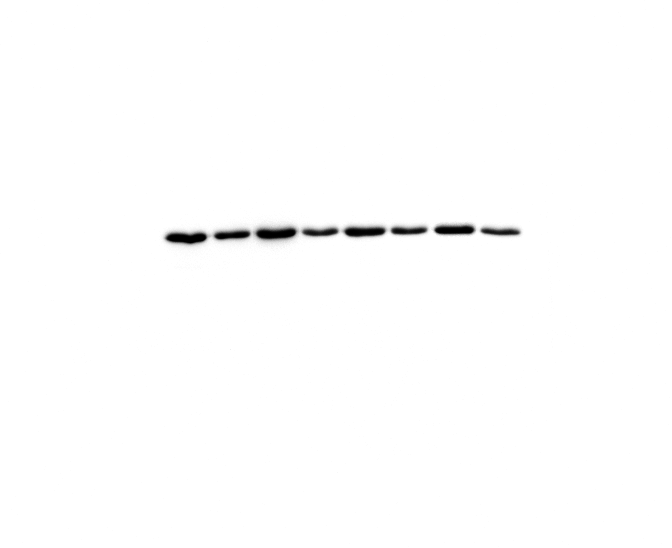


Fig. 3E β-actin


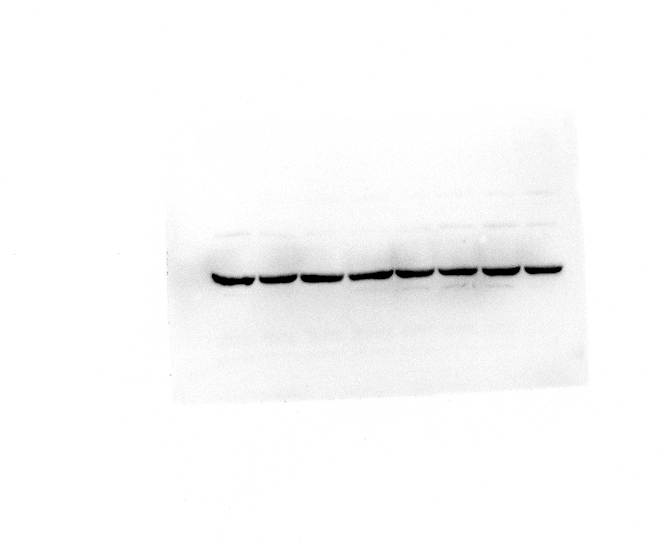


Fig. 4B CDK2


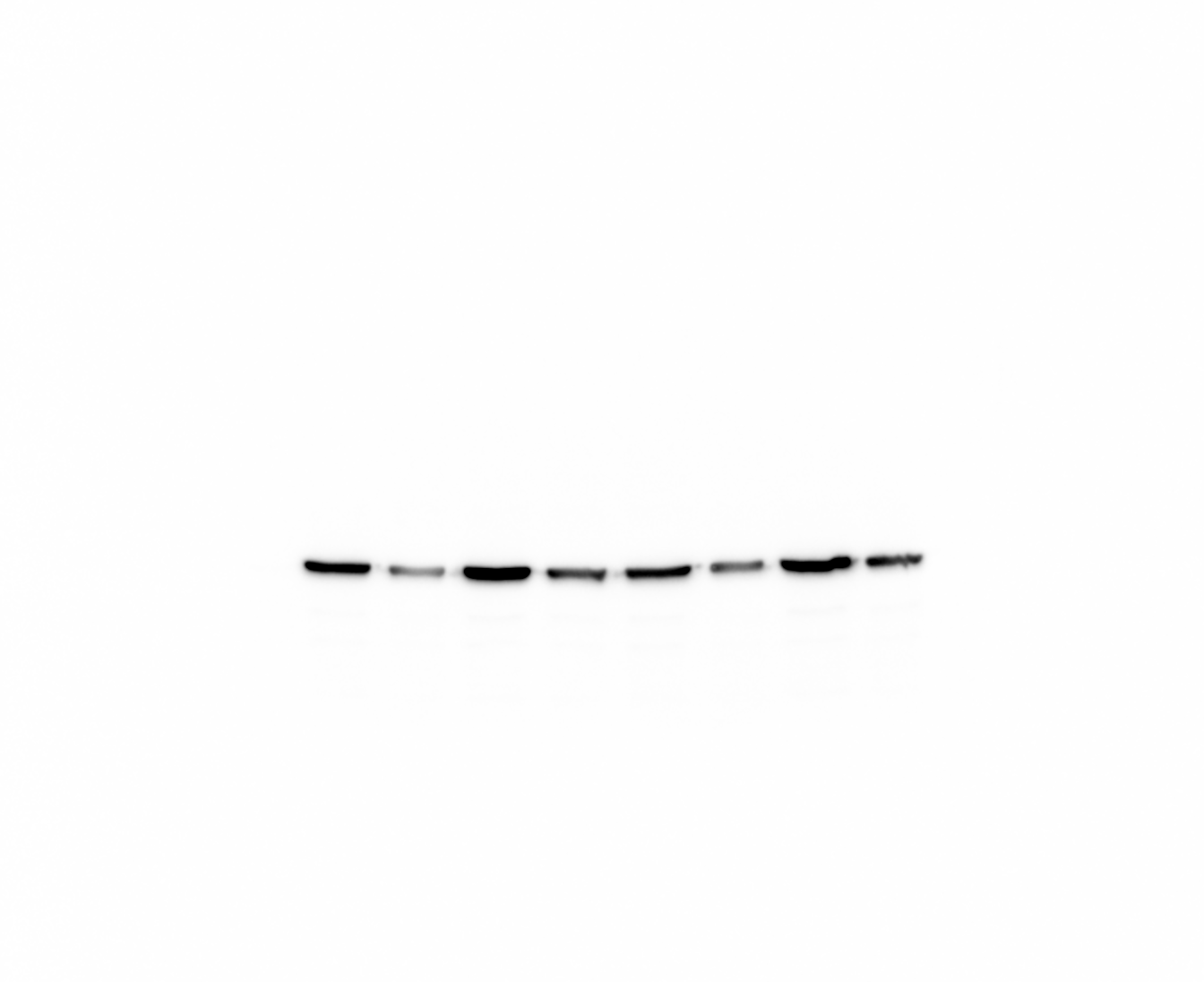


Fig. 4B CDK4


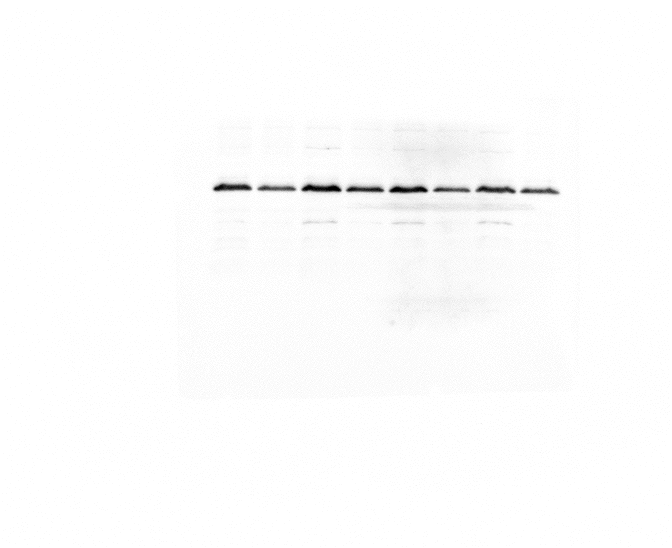


Fig. 4B CDK6


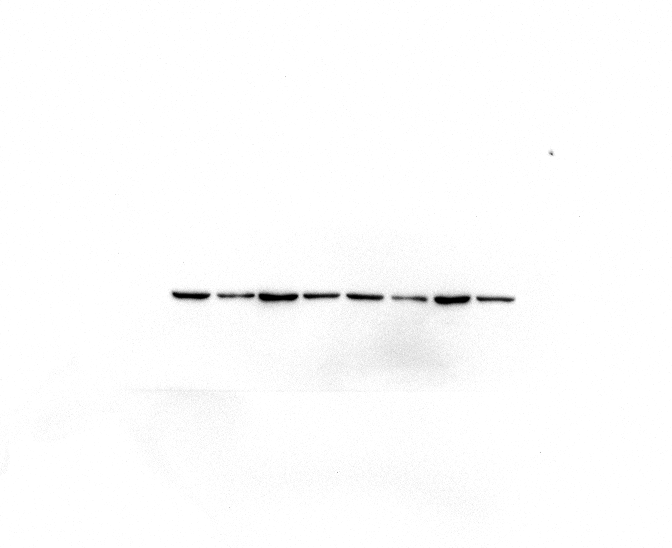


Fig. 4B Cyclin D1


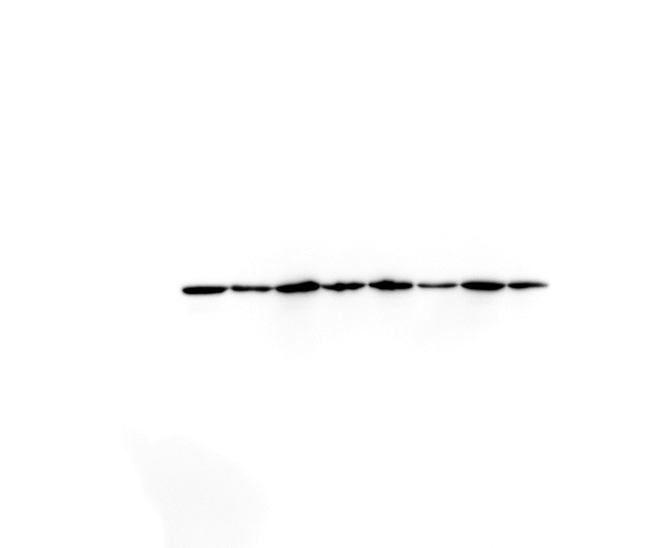


Fig. 4B Cyclin D1


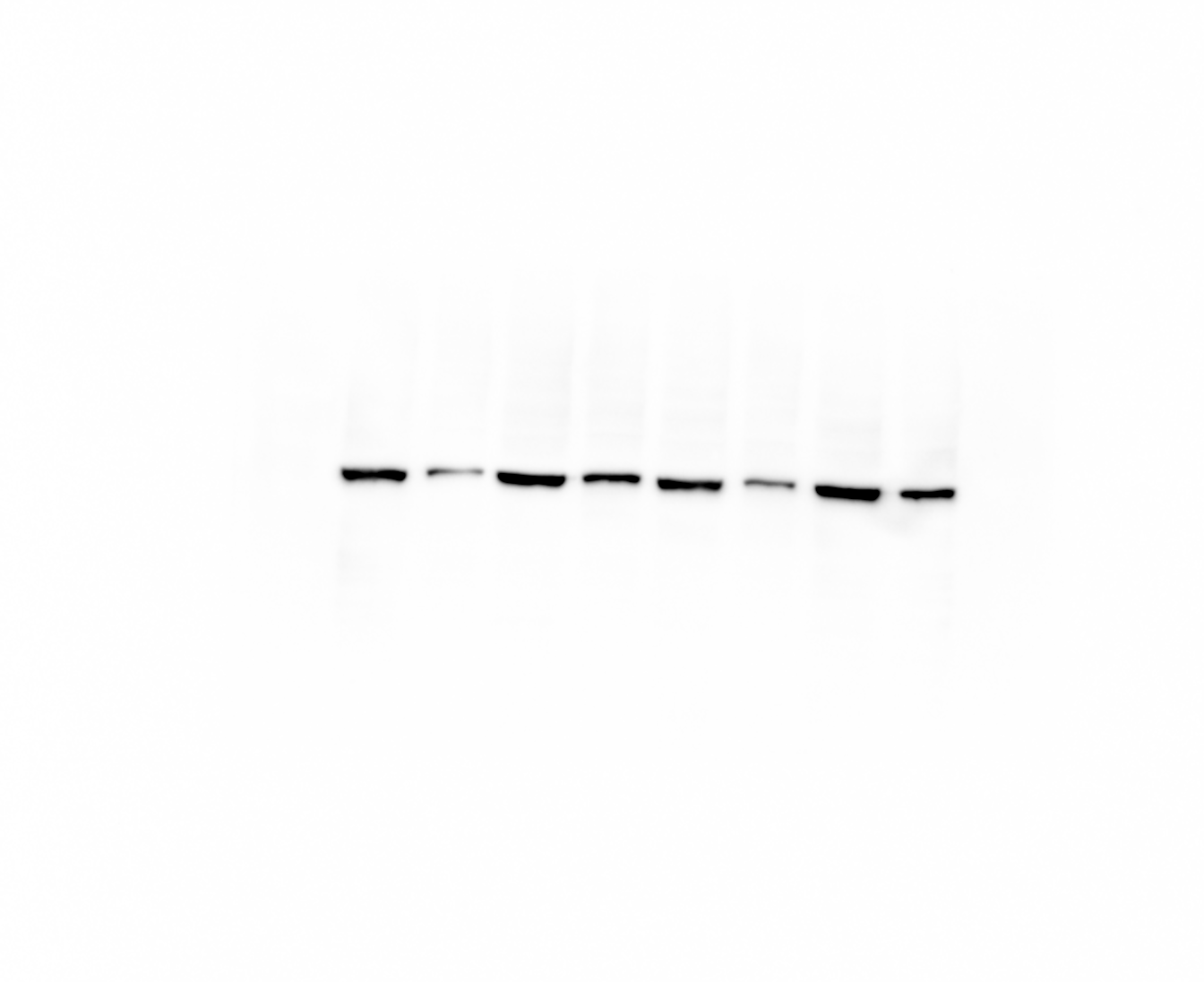


Fig. 4B P-Rb


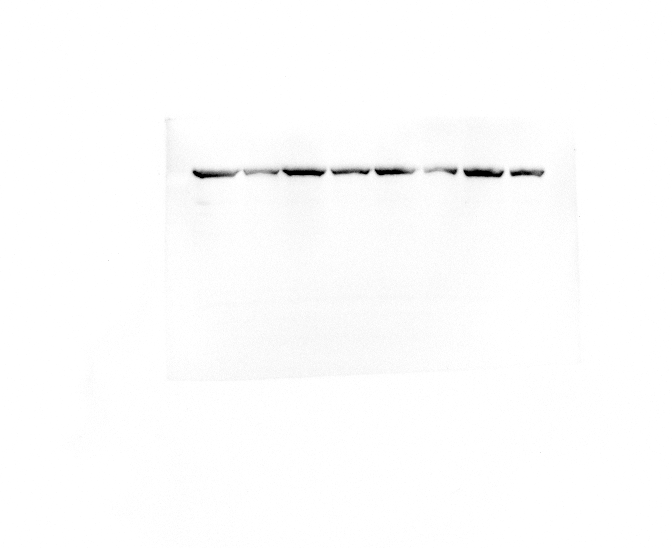


Fig. 4B β-actin


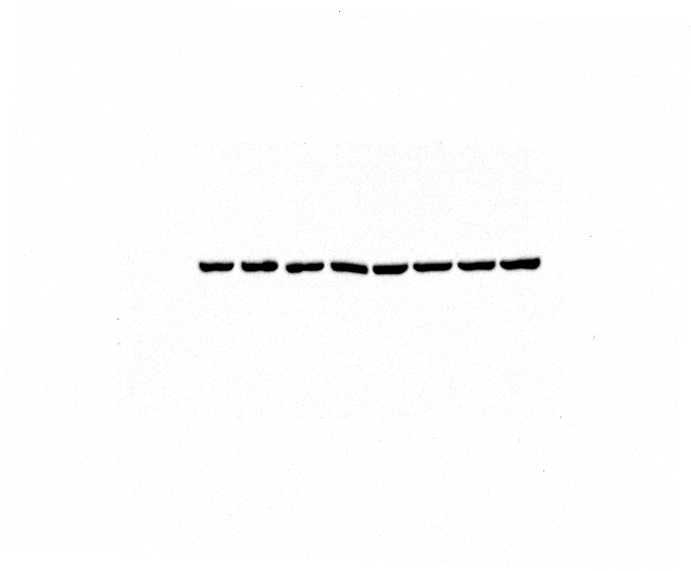


Fig. 4C CDK2


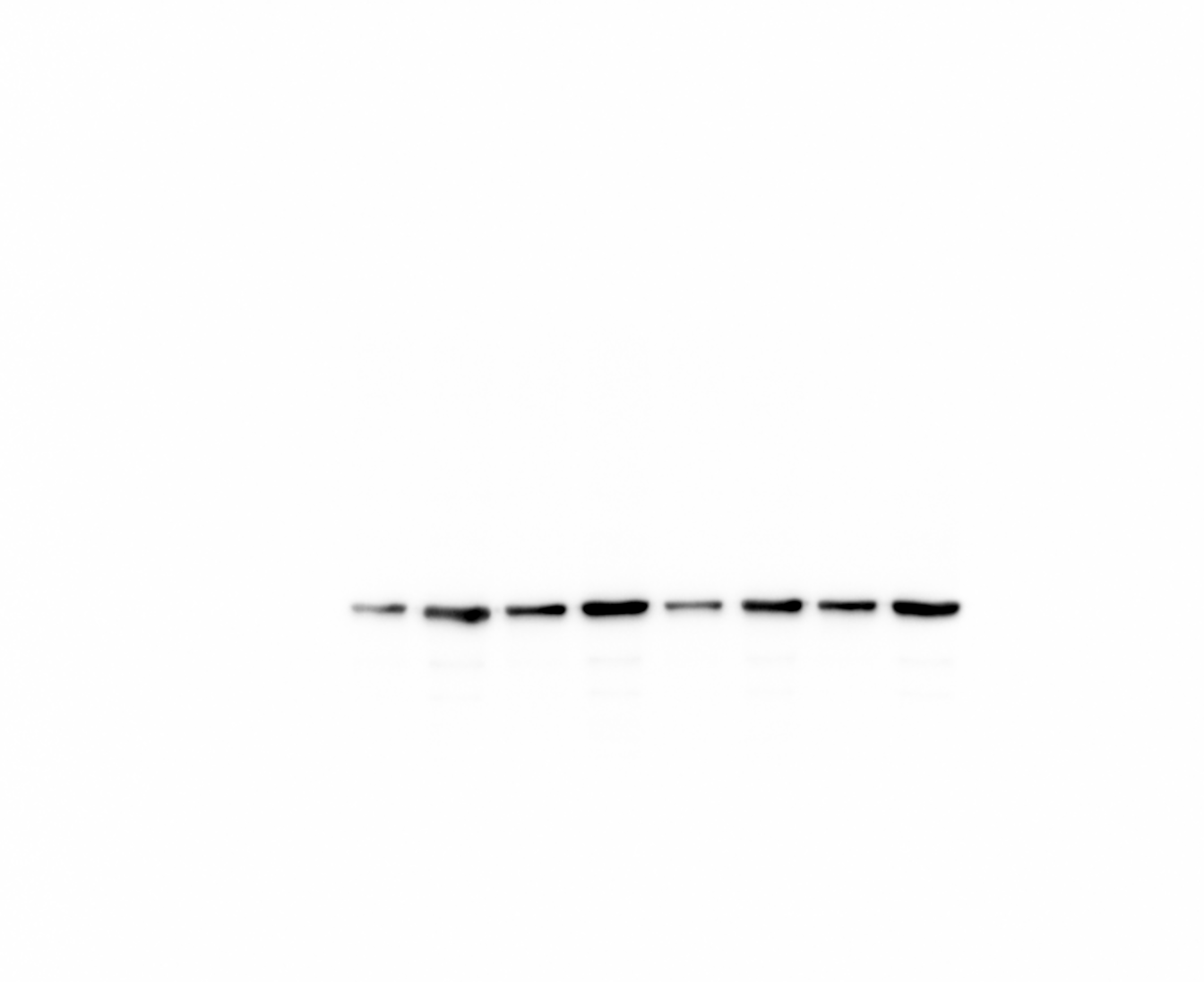


Fig. 4C CDK4


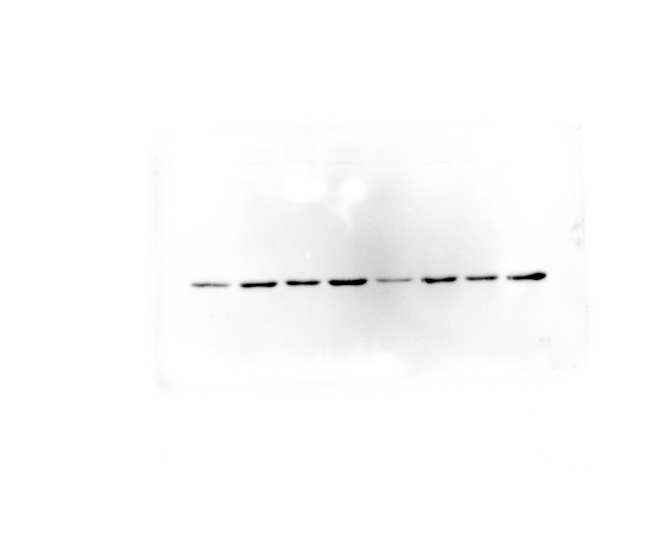


Fig. 4C CDK6


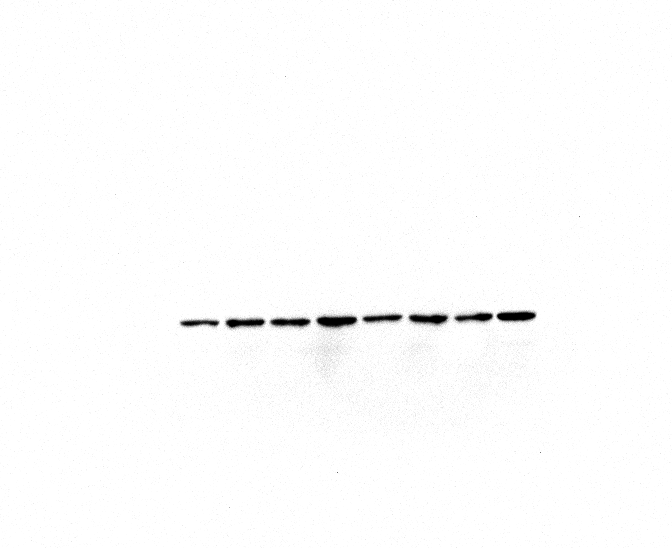


Fig. 4C Cyclin D1


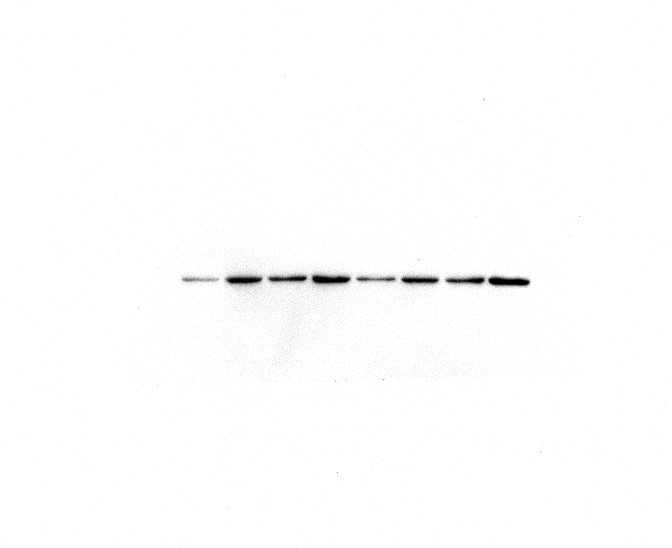


Fig. 4C Cyclin E1


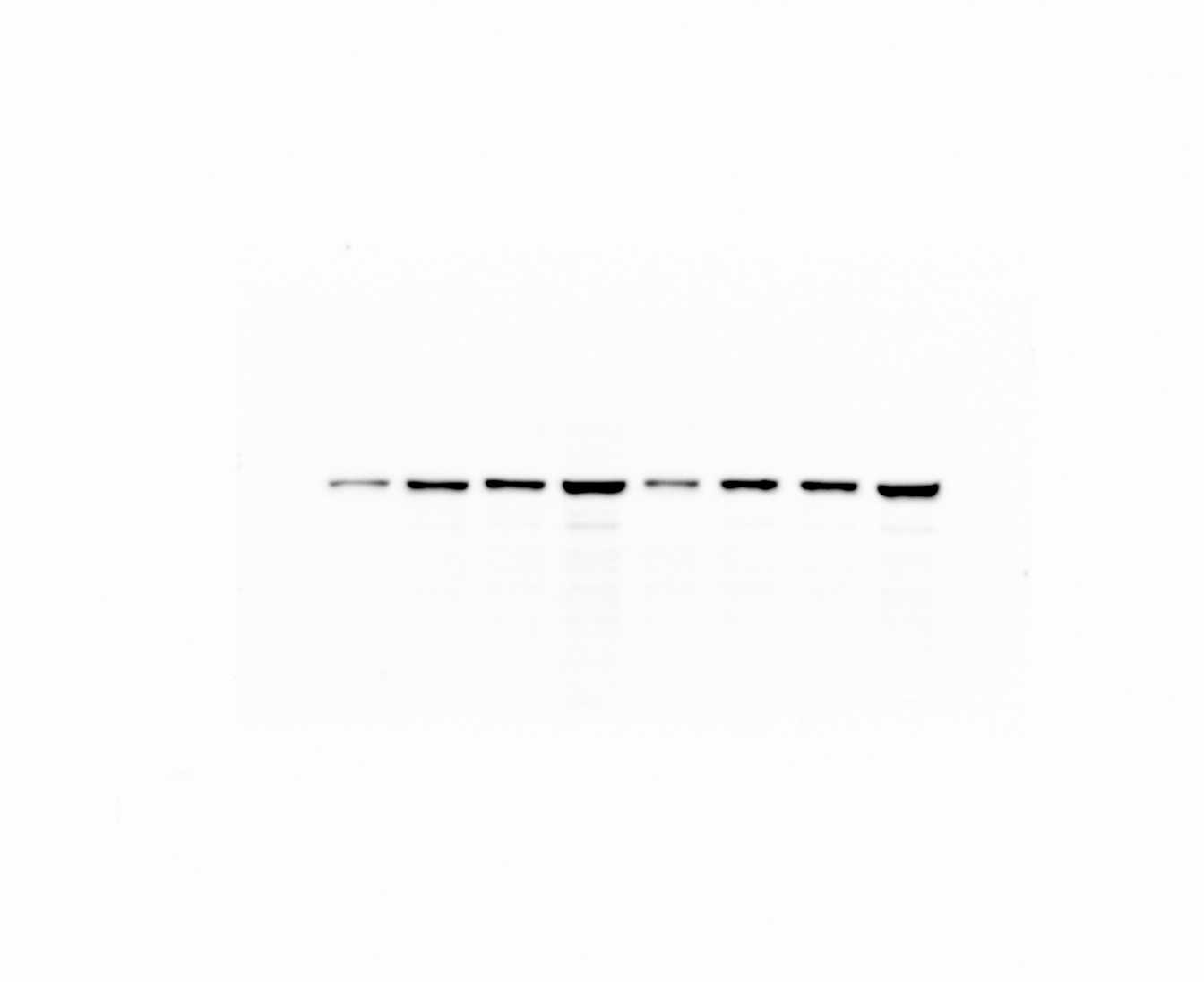


Fig. 4C P-Rb


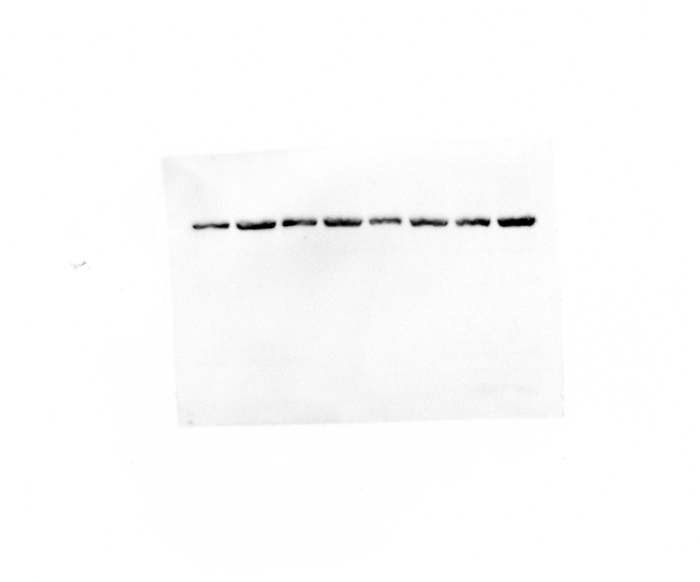


Fig. 4C β-actin


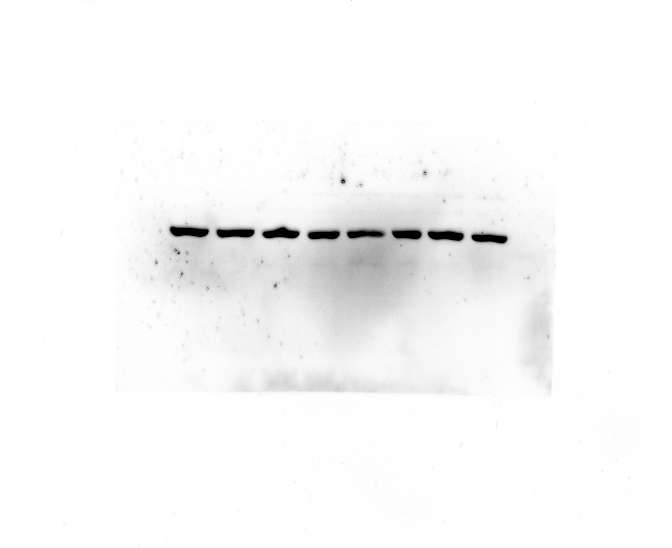


Fig. 5C Akt


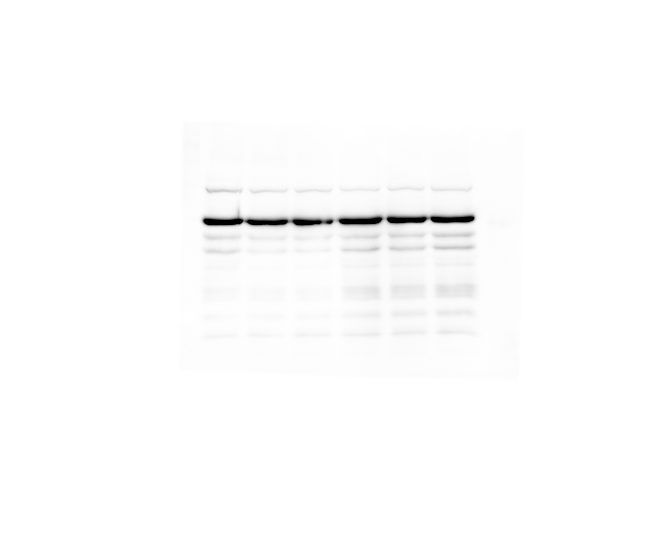


Fig. 5C P-Akt


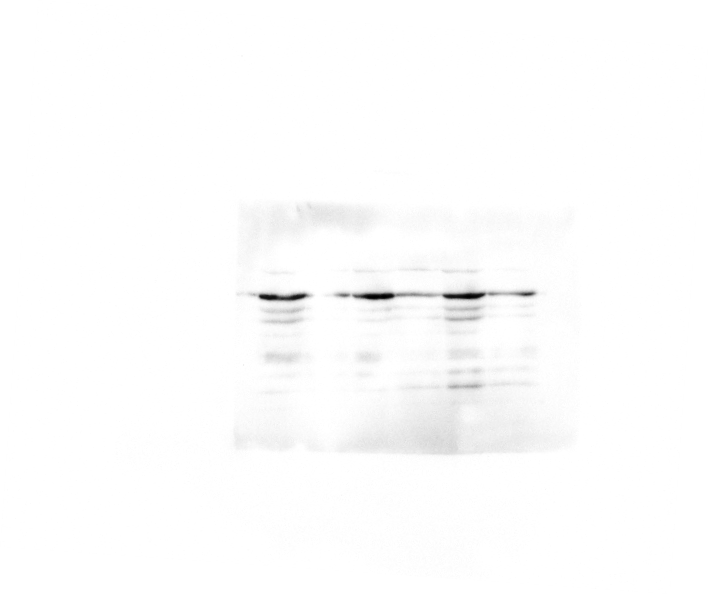


Fig. 5C PI3K


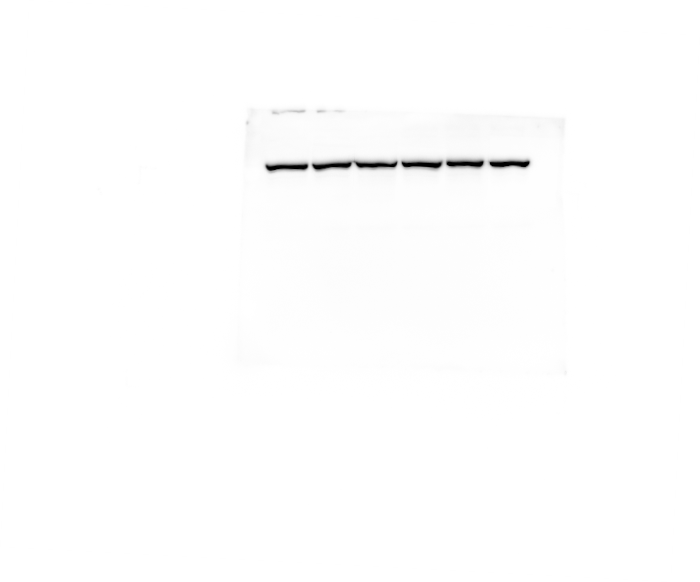


Fig. 5C P-PI3K


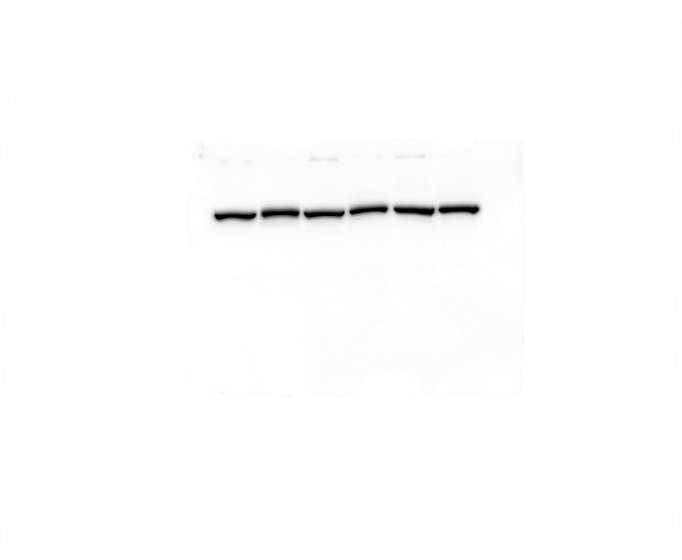


Fig. 5C β-actin


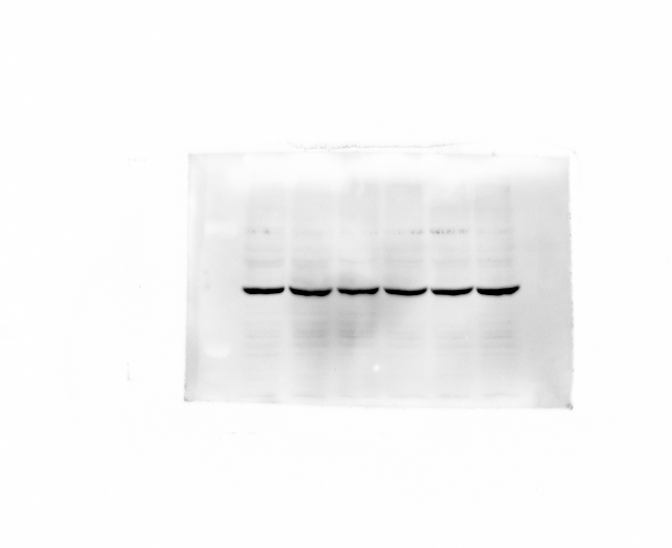


Fig. 5D Bax


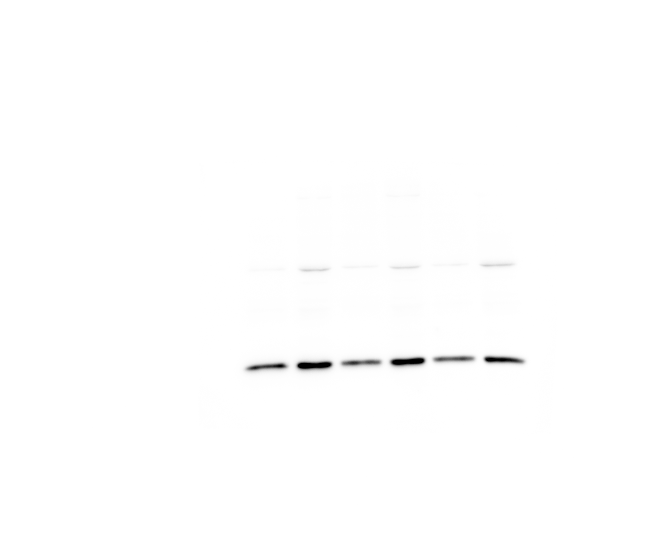


Fig. 5D Bcl-2


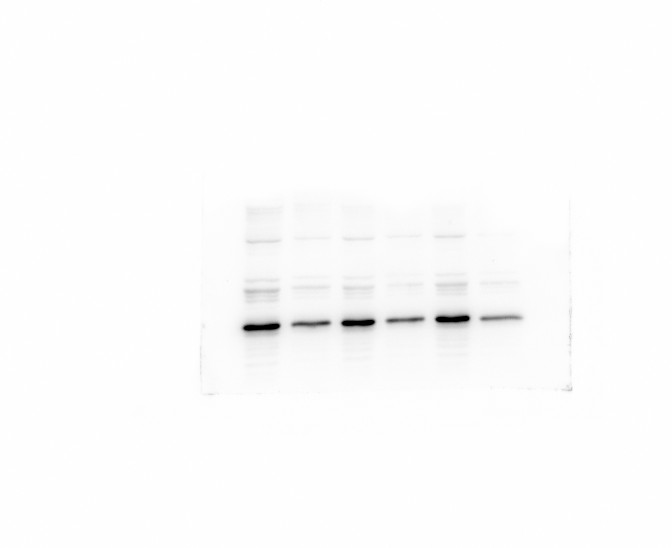


Fig. 5D Cleaved Caspase-3


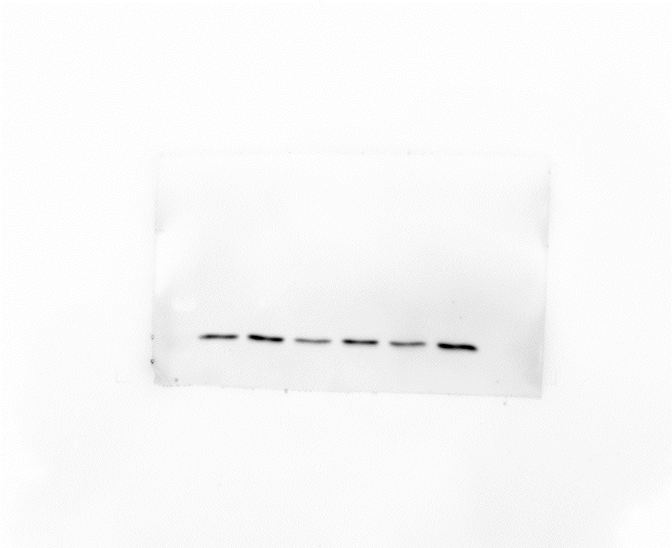


Fig. 5D Cleaved Caspase-9


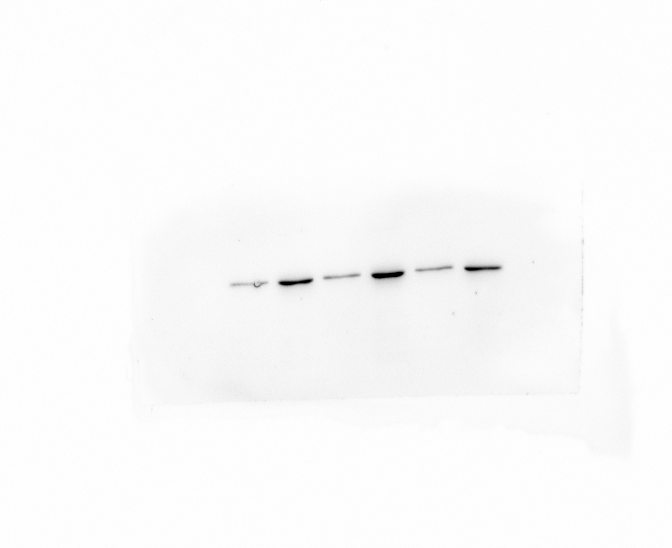


Fig. 5D β-actin


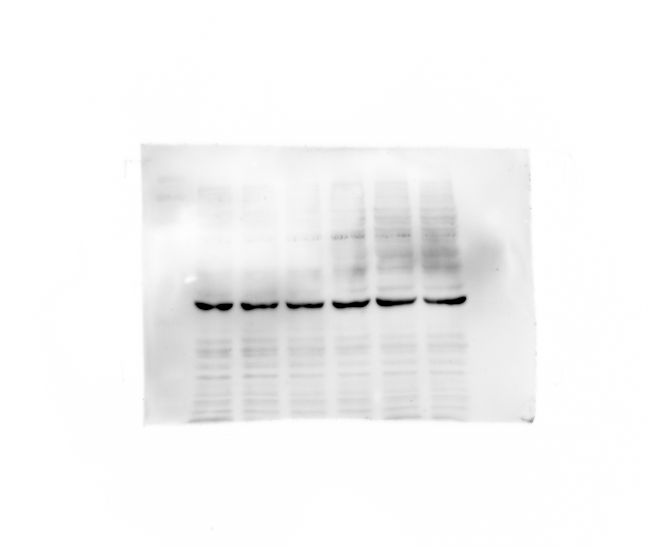


Fig. 5E CDK4


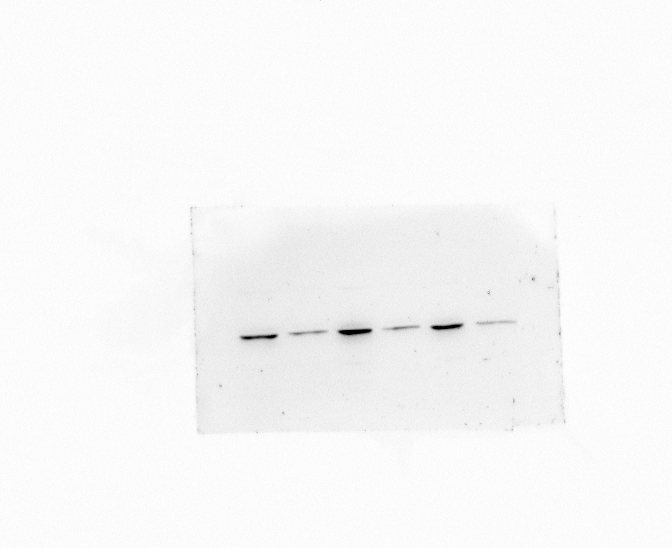


Fig. 5E CDK6


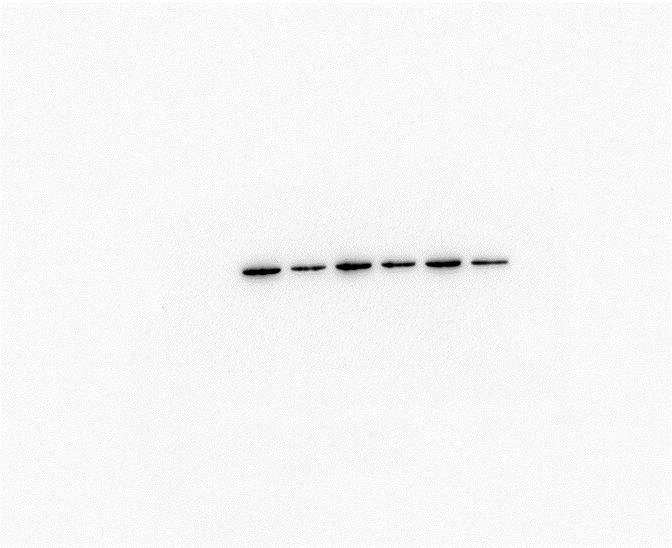


Fig. 5E CyclinD1


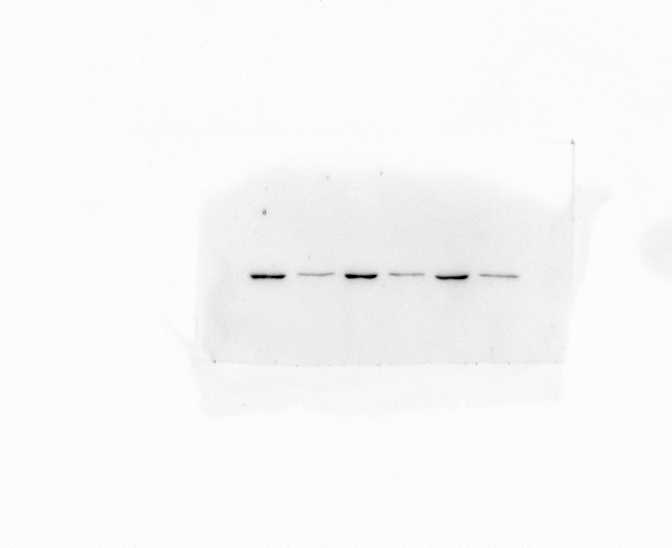


Fig. 5E P-Rb


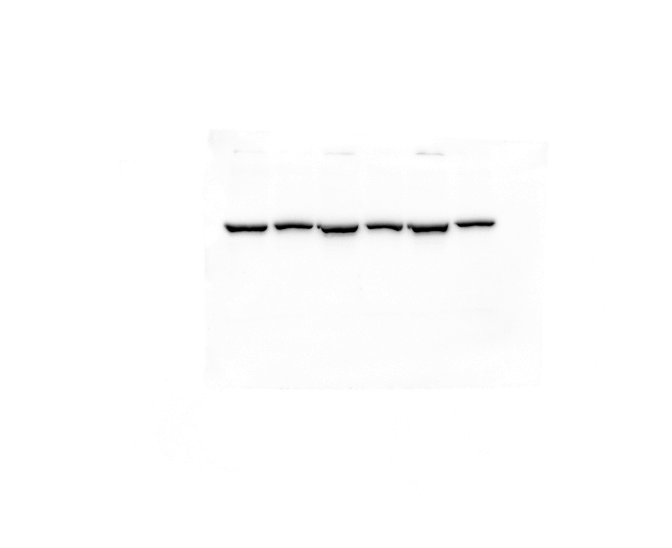


Fig. 5E β-actin


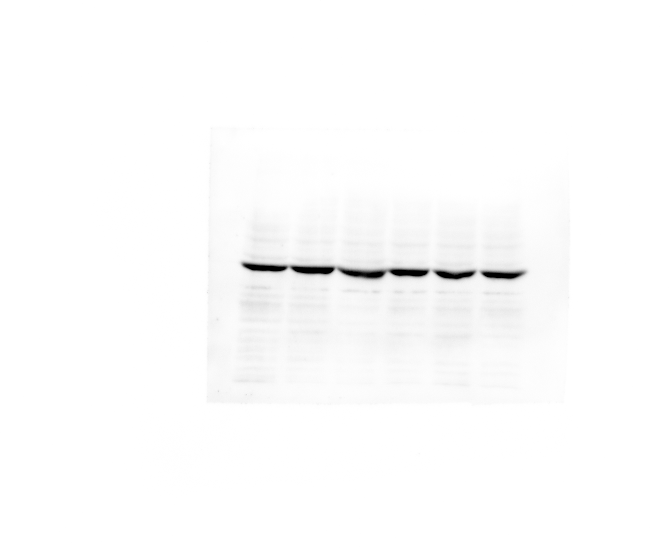


Fig. 7B input-Flag


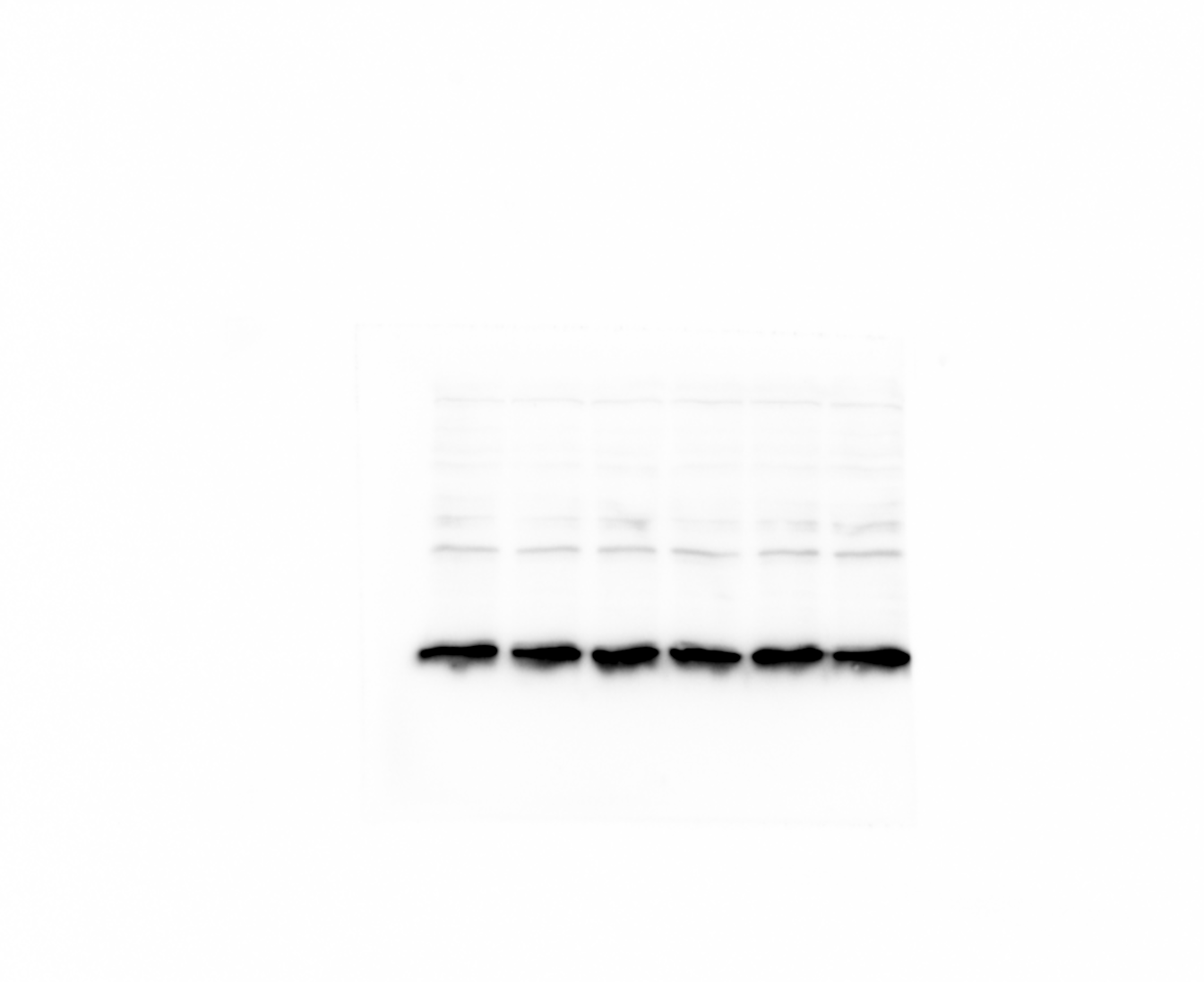


Fig. 7B input-HA


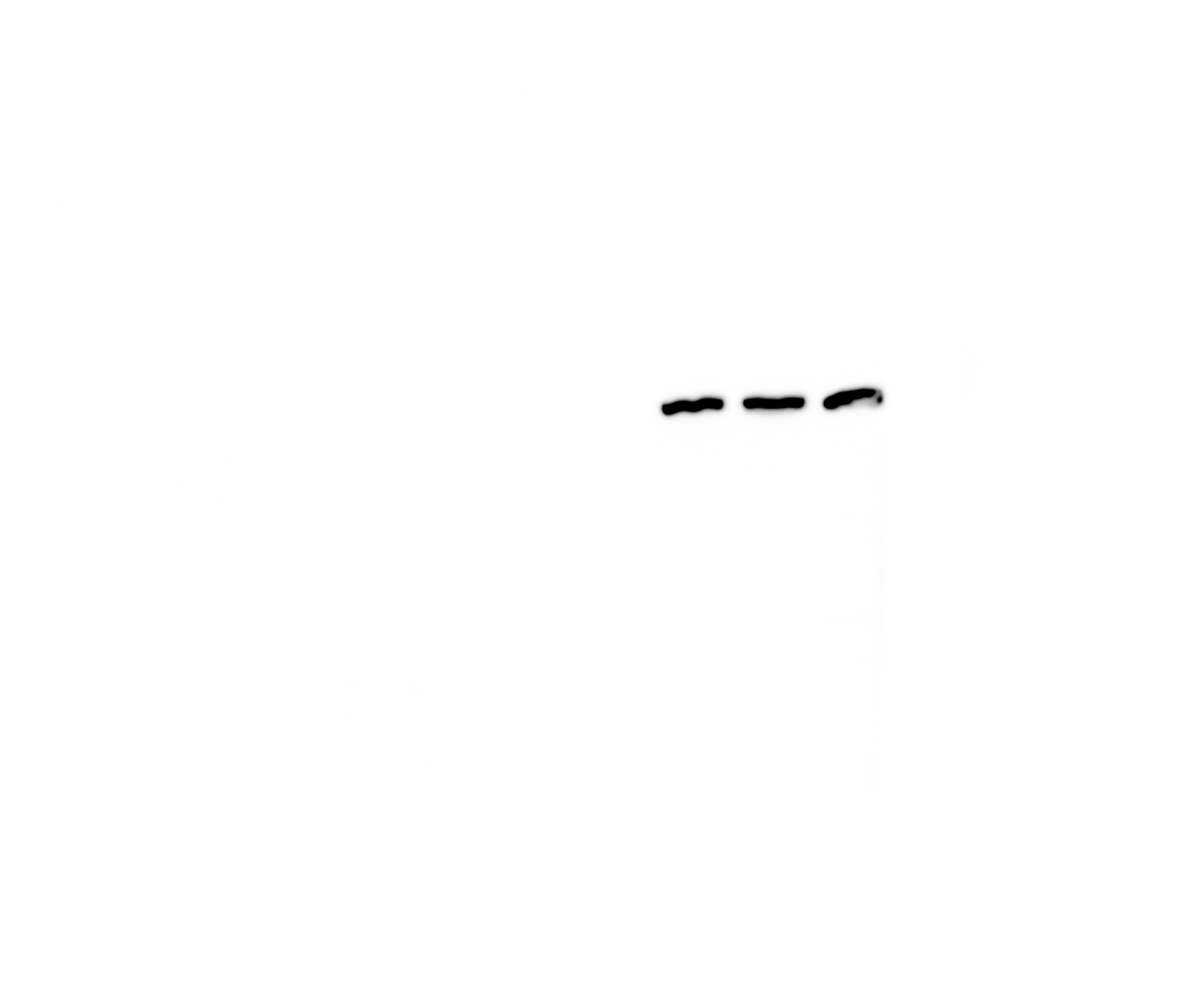


Fig. 7B IP-Flag+IB-Flag


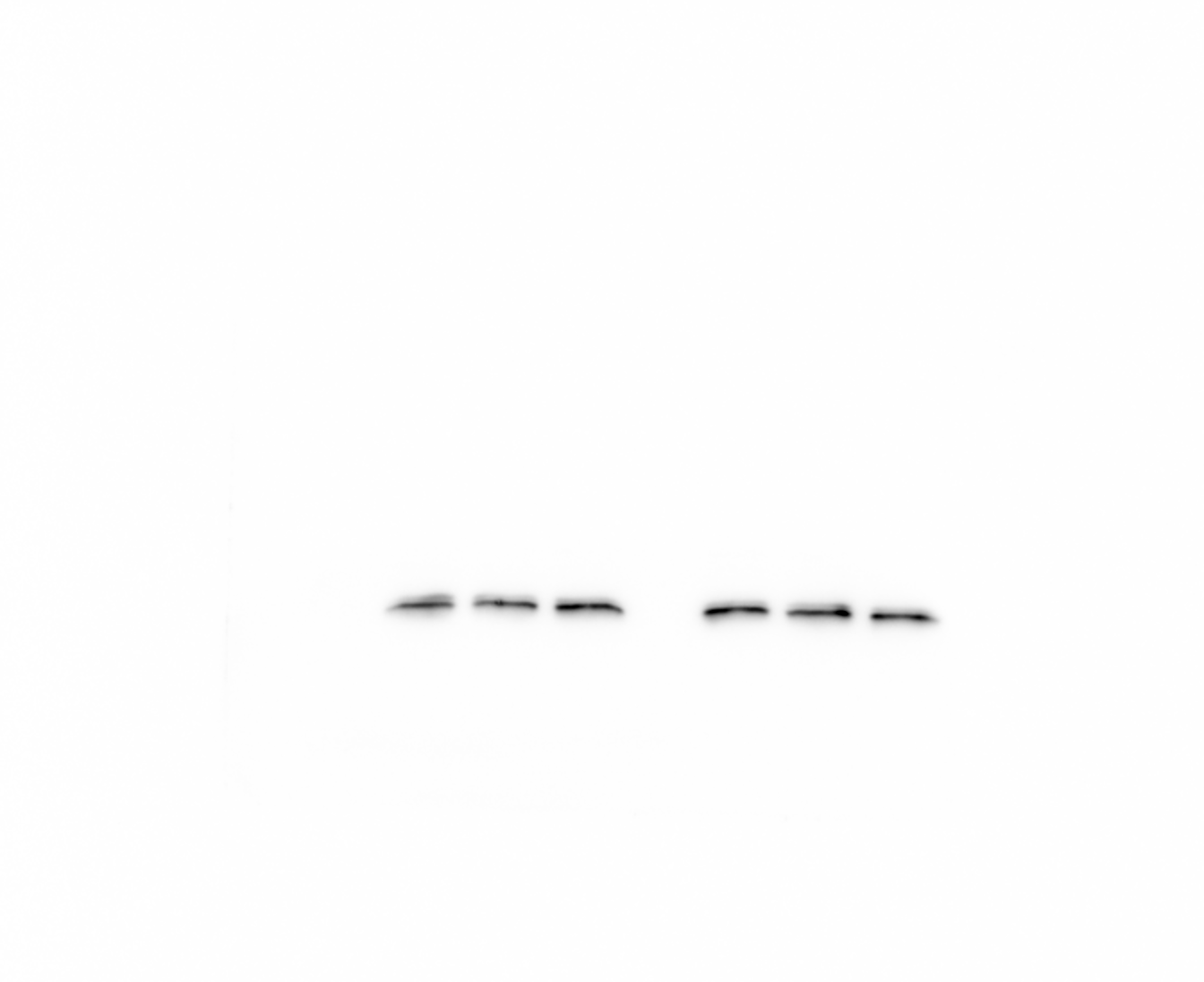


Fig. 7B IP-Flag+IB-HA


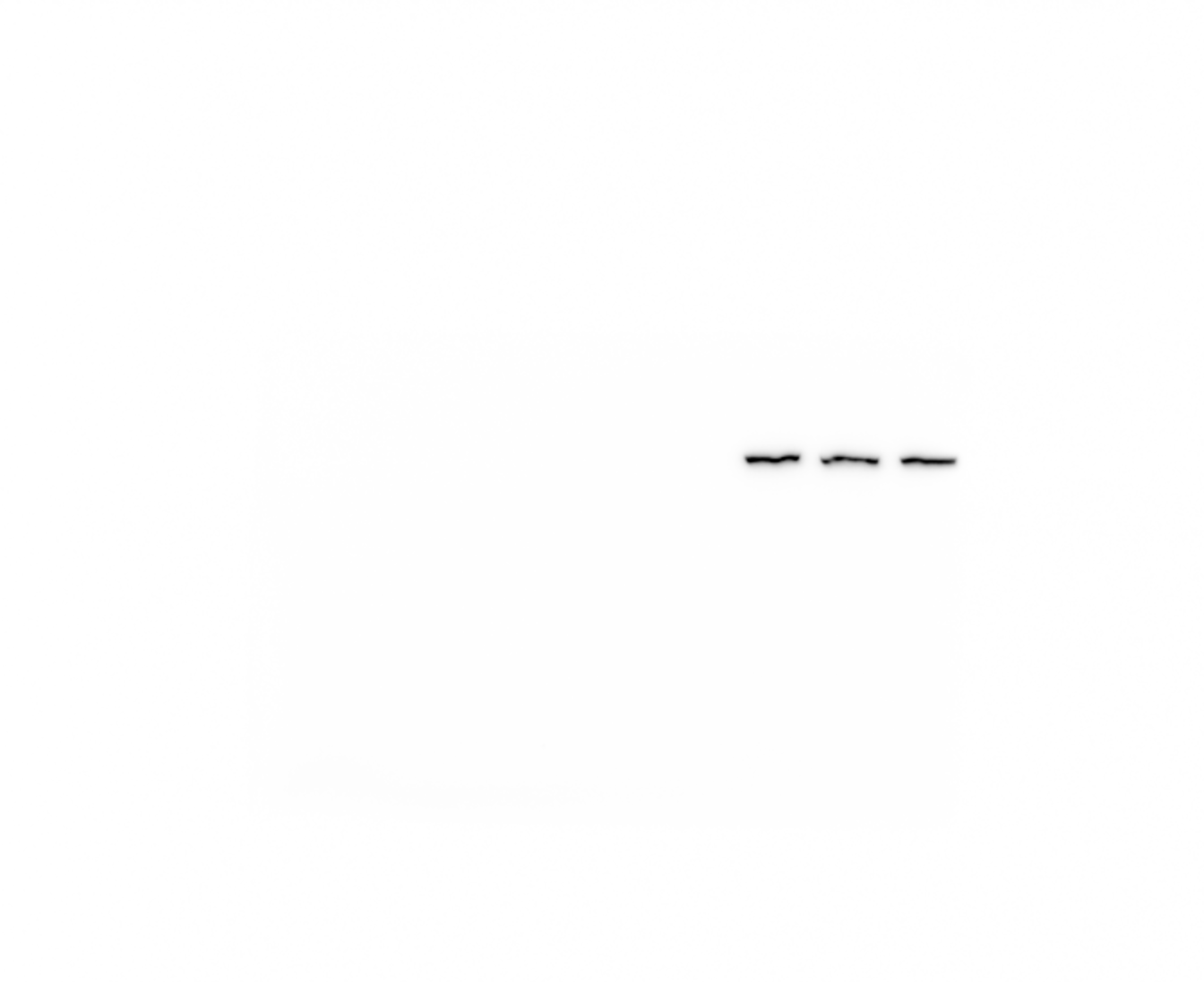


Fig. 7D Cleaved Notch1


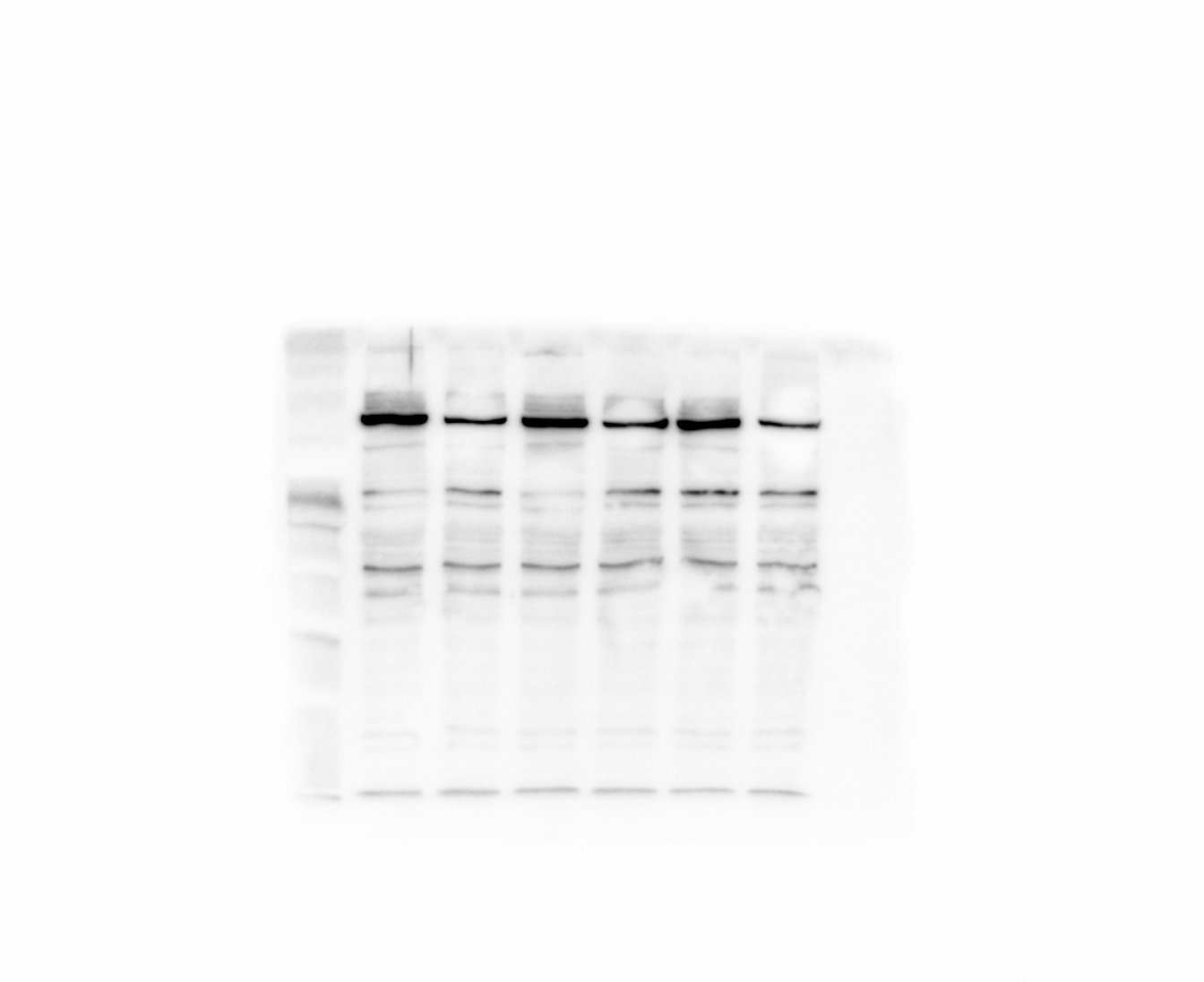


Fig. 7D Hes1


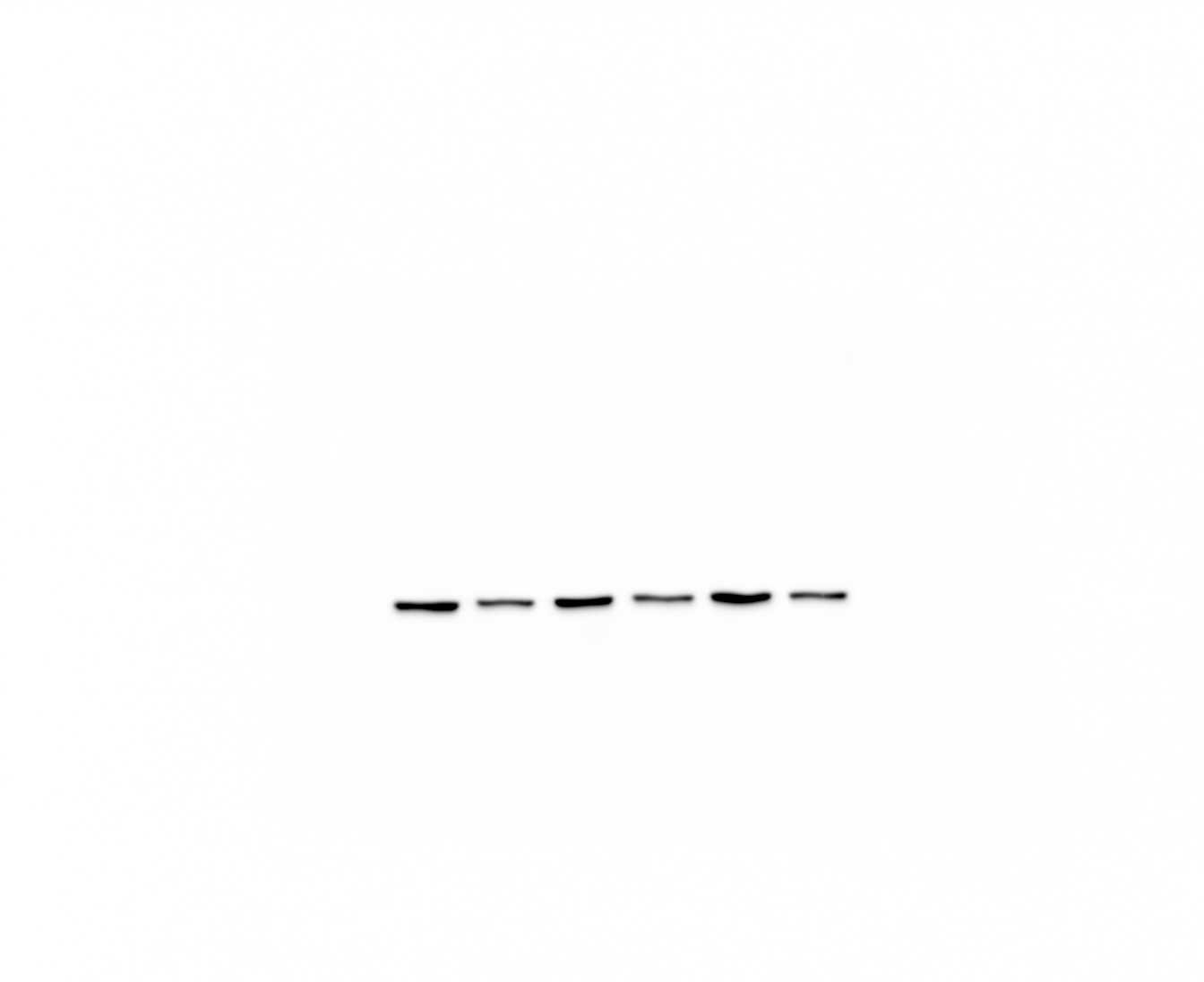


Fig. 7D PTEN


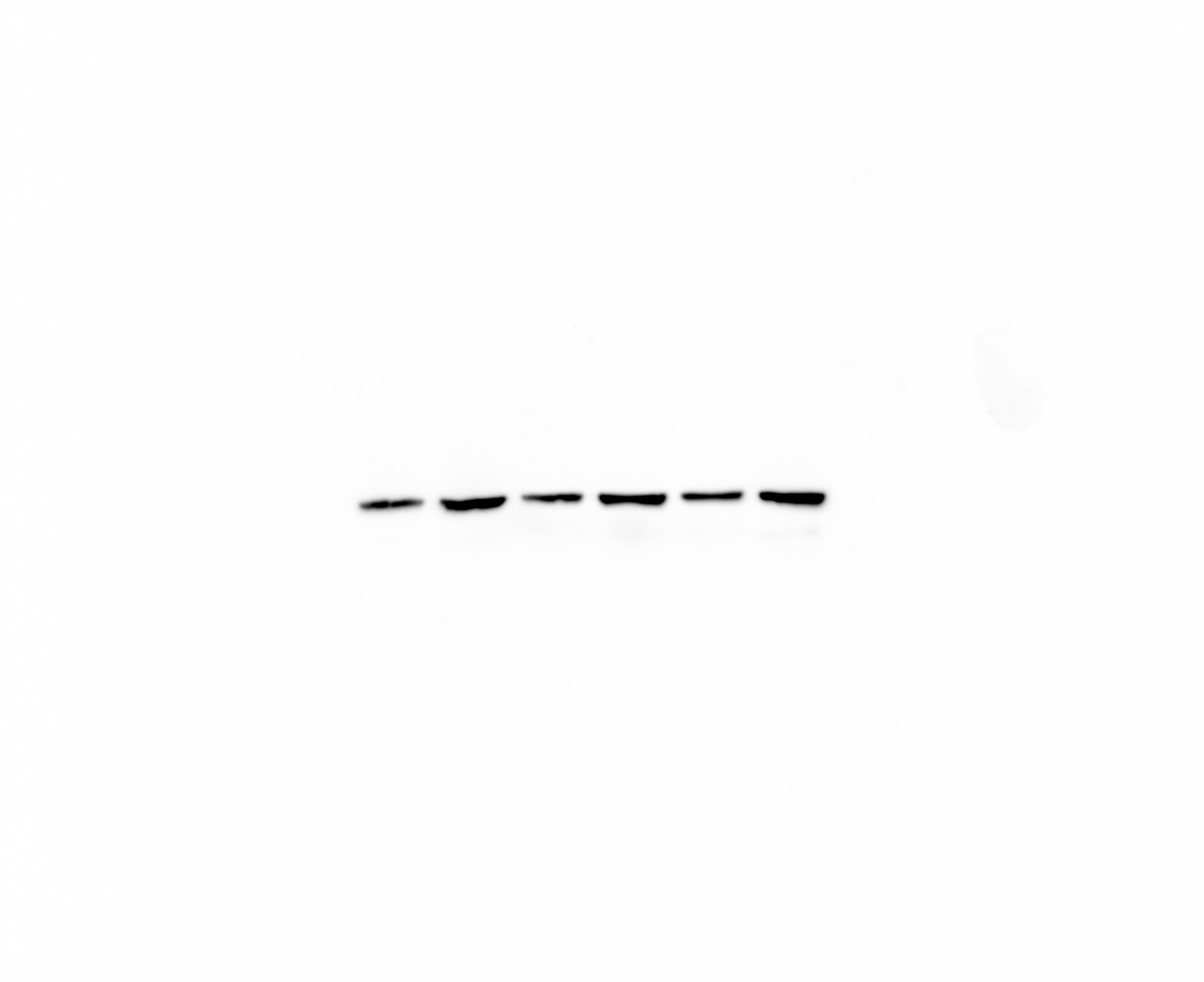


Fig. 7D Akt


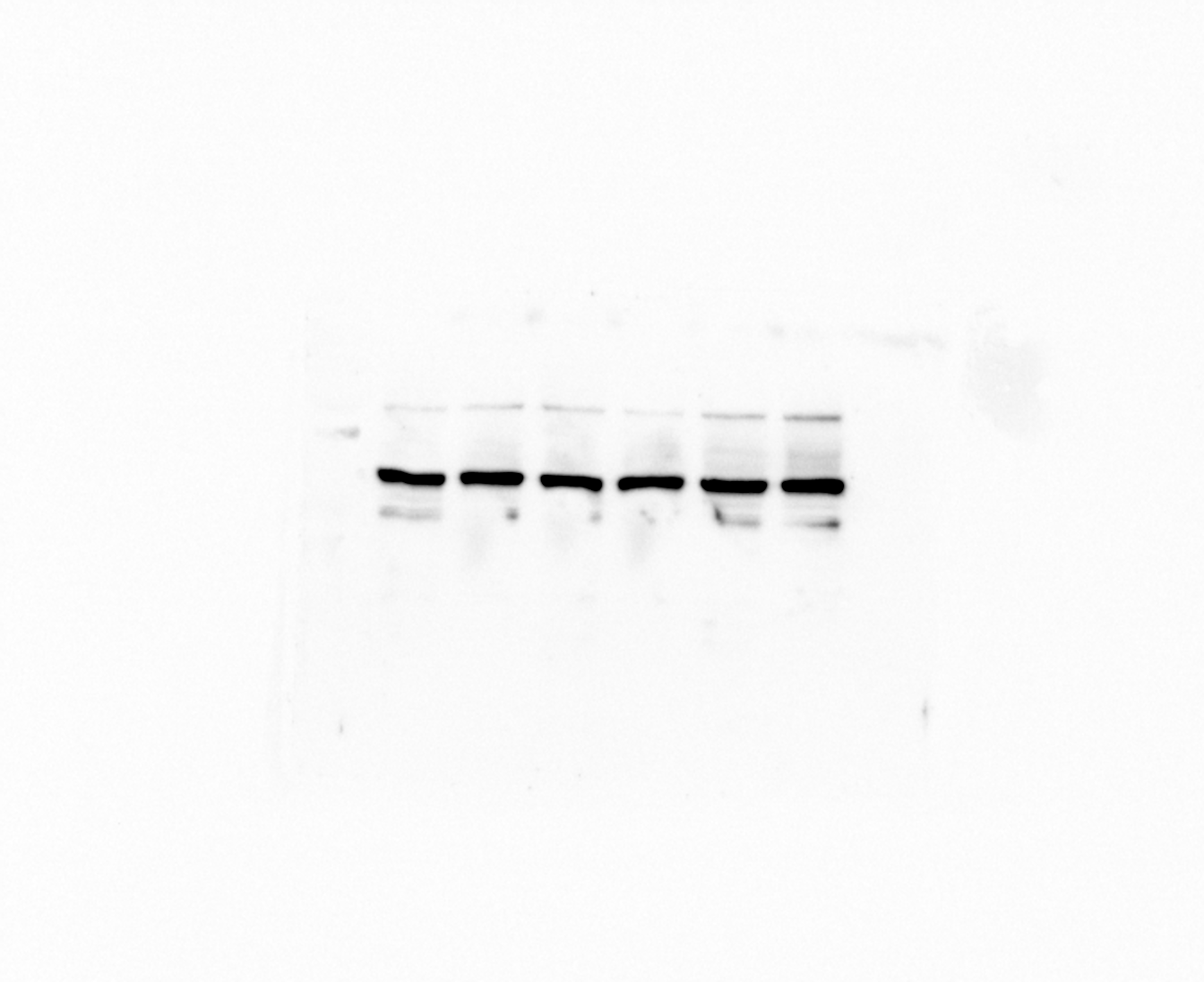


Fig. 7D P-Akt


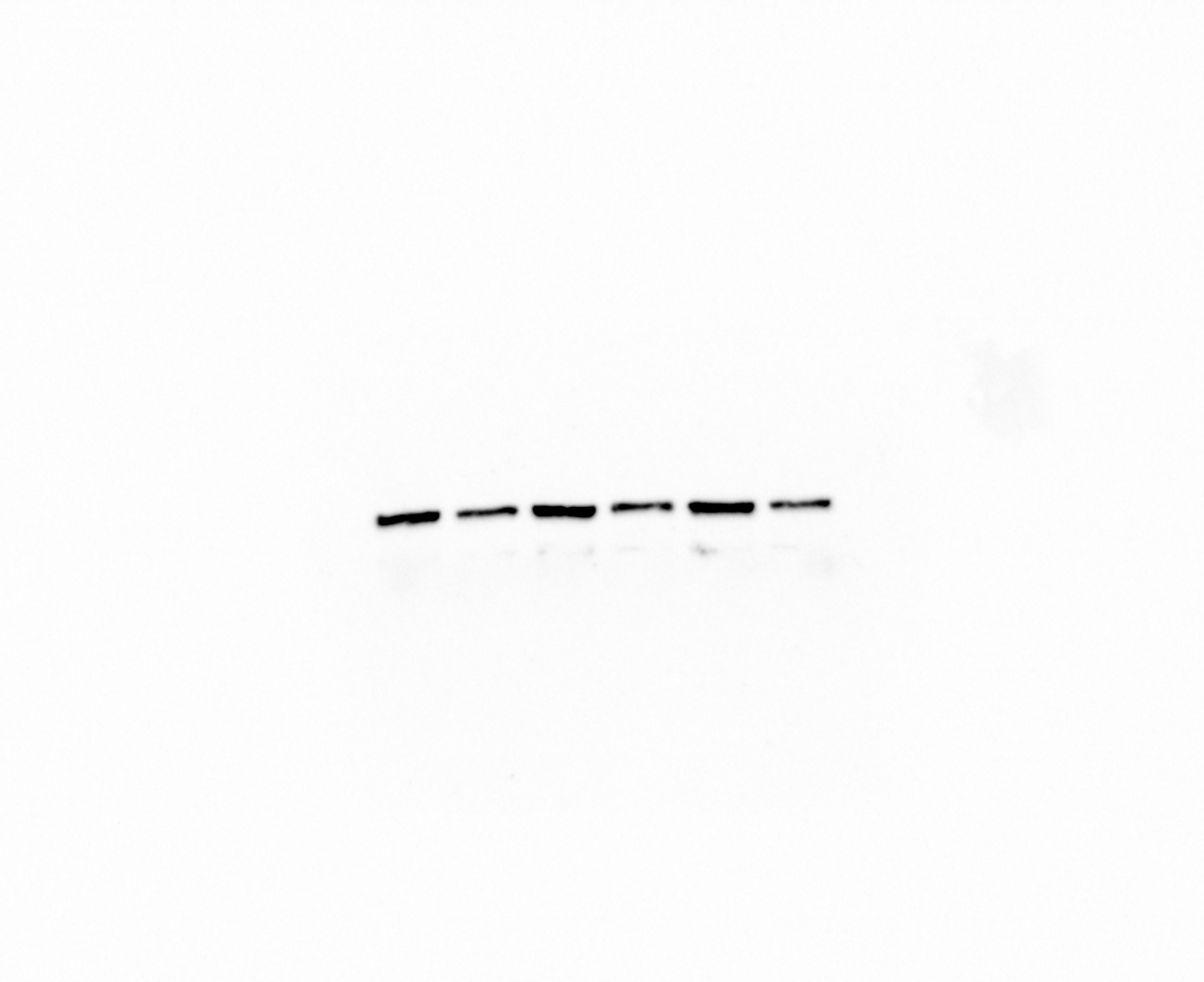


Fig. 7D E-cadherin


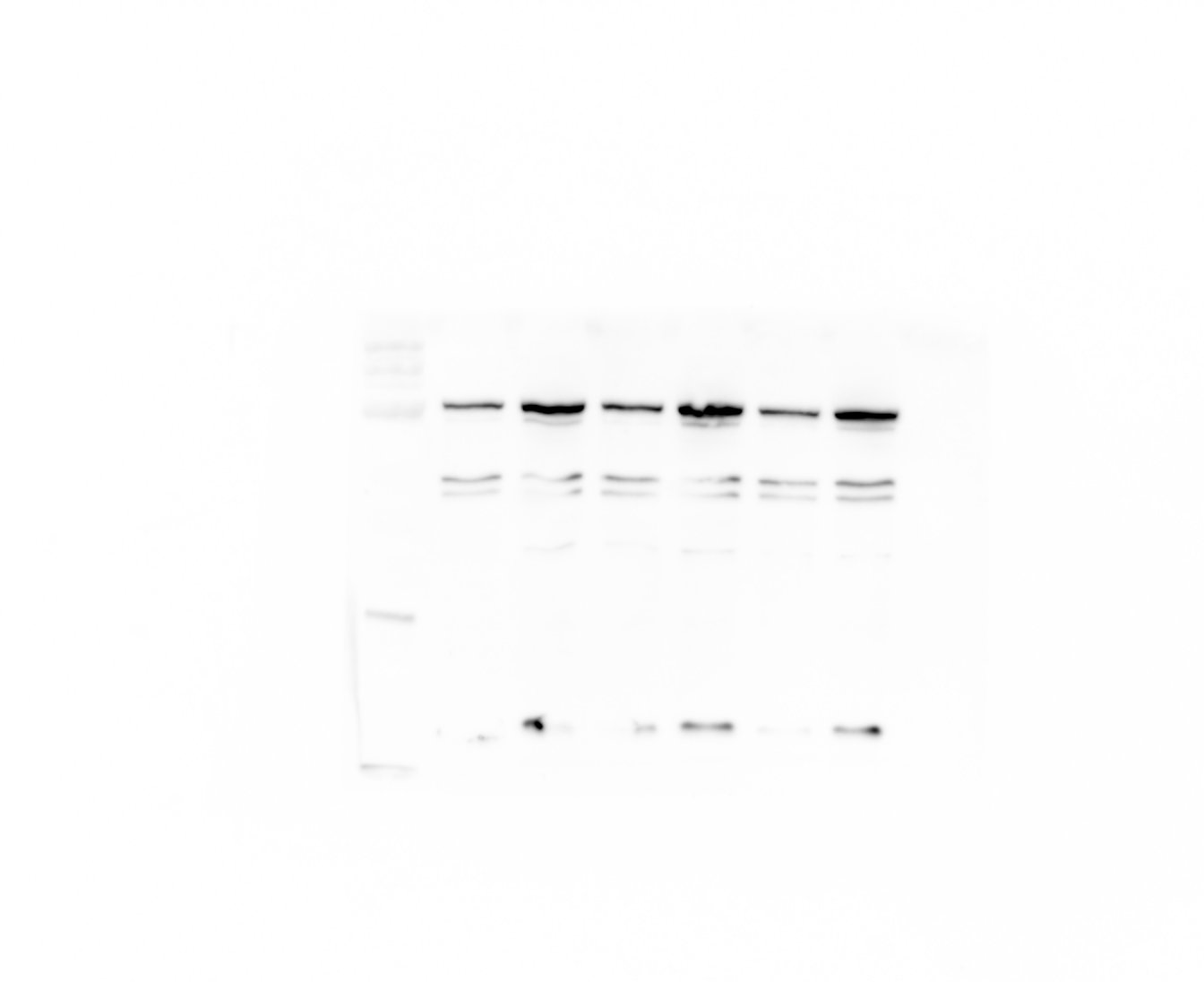


Fig. 7D N-cadherin


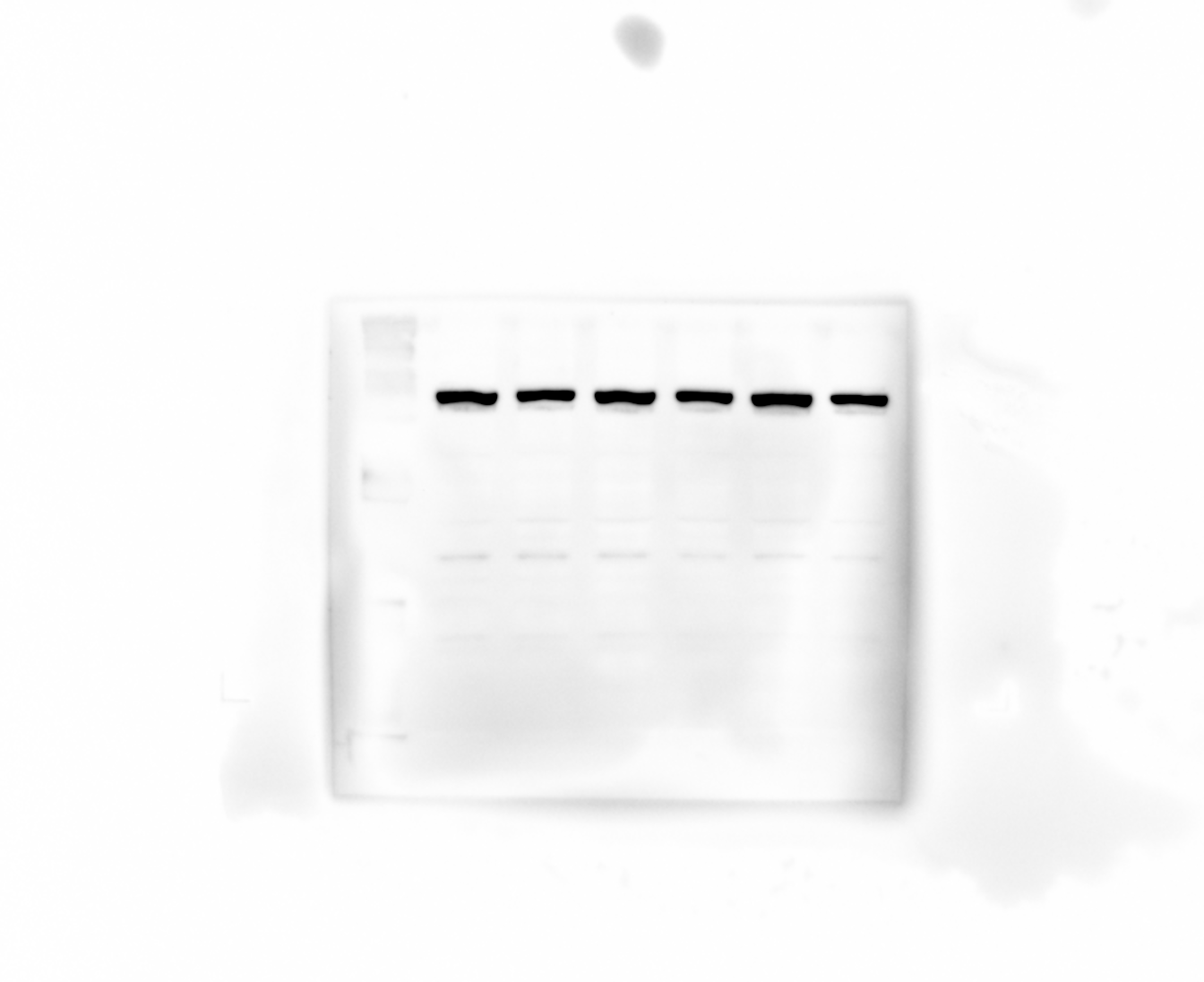


Fig. 7D Vimentin


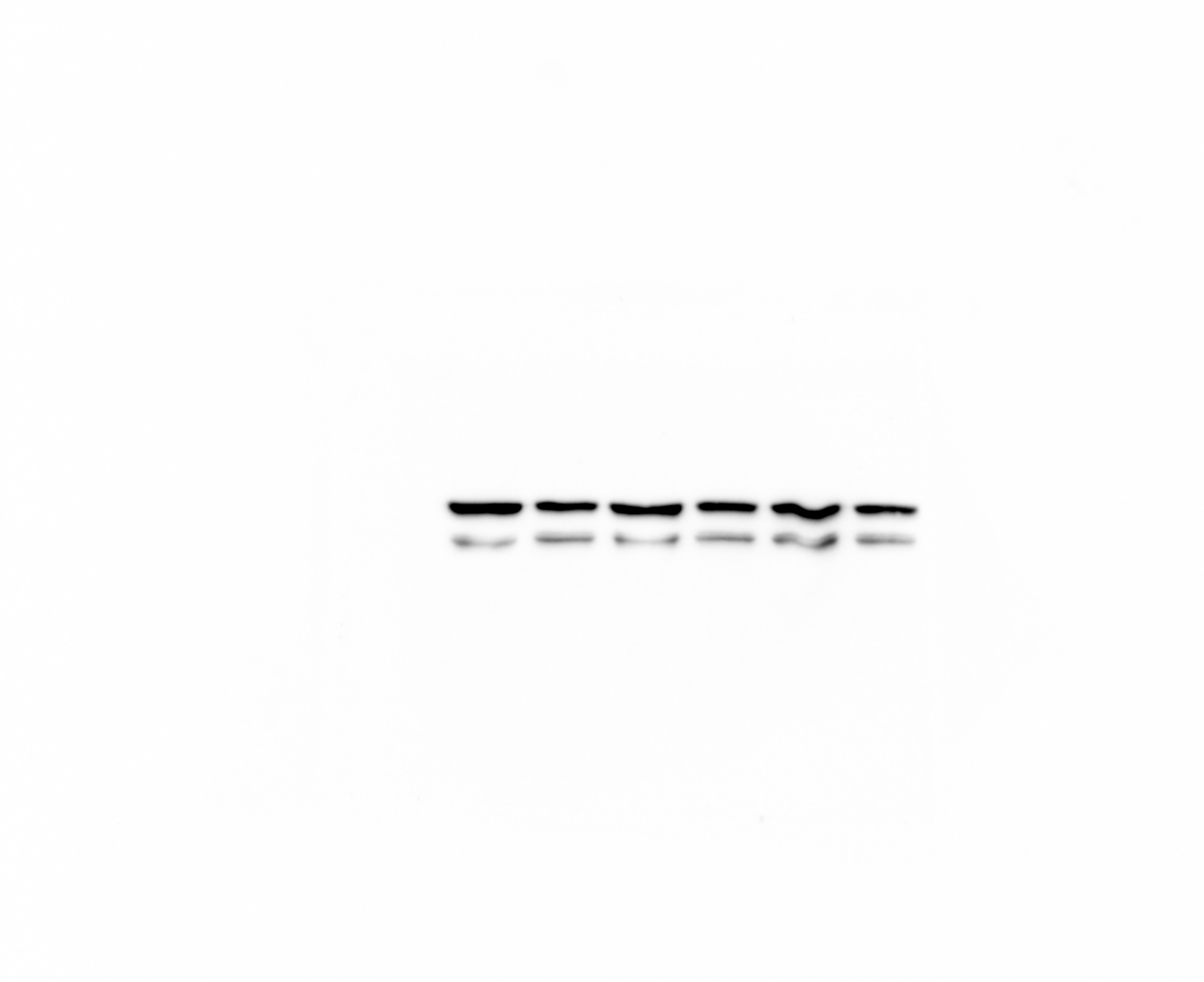


Fig. 7D β-actin


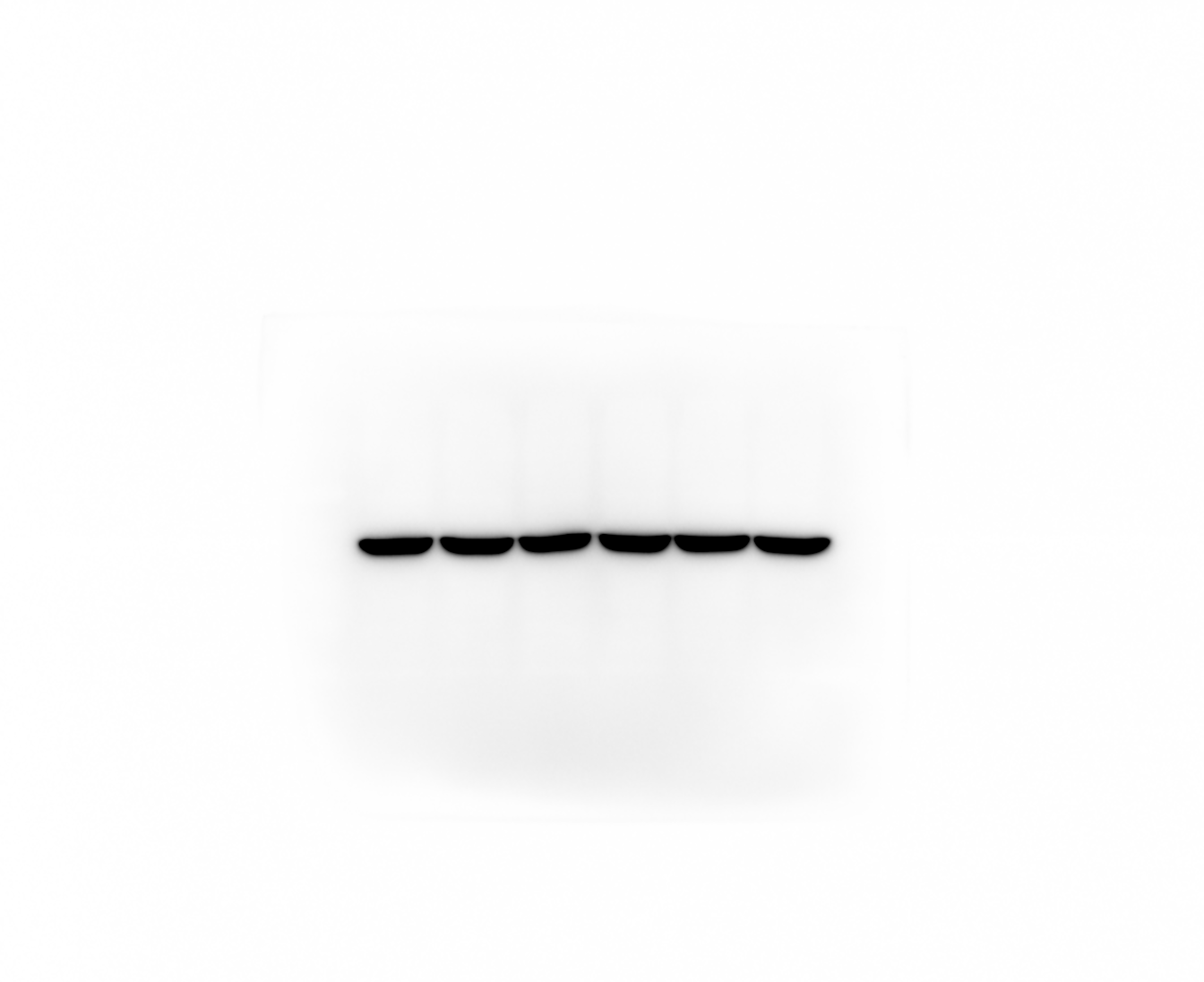

Supplement: Supplementary file 2 — Original WB [file 41419_2024_7214_MOESM2_ESM.docx]
