# Supplementary material for: SPINK13 acts as a tumor suppressor in hepatocellular carcinoma by inhibiting Akt phosphorylation
Source: Cell Death Dis. 2024 Nov 13;15(11):822. doi: 10.1038/s41419-024-07214-3 (PMC11561306; doi:10.1038/s41419-024-07214-3)
Supplement: Supplementary file 3 — Supplementary Tables [file 41419_2024_7214_MOESM3_ESM.docx]

Table S1. qPCR primer sequences.

| Gene | Primer sequences (5'-3') | |
| --- | --- | --- |
| *GAPDH* | F | AGAAGGCTGGGGCTCATTTG |
|  | R | AGGGGCCATCCACAGTCTTC |
| *SPINK13* | F | ACTTCACTAGGTGGCCTAAGC |
|  | R | TCATTCTGGAAAGTGTGGCCA |
| *AGO2* | F | TCCACCTAGACCCGACTTTGG |
|  | R | GTGTTCCACGATTTCCCTGTT |
| *CREBBP* | F | CAACCCCAAAAGAGCCAAACT |
|  | R | CCTCGTAGAAGCTCCGACAGT |
| *JUN* | F | TCCAAGTGCCGAAAAAGGAAG |
|  | R | CGAGTTCTGAGCTTTCAAGGT |
| *E2F1* | F | ACGCTATGAGACCTCACTGAA |
|  | R | TCCTGGGTCAACCCCTCAAG |
| *PCNA* | F | CCTGCTGGGATATTAGCTCCA |
|  | R | CAGCGGTAGGTGTCGAAGC |
| *BRCA1* | F | GAAACCGTGCCAAAAGACTTC |
|  | R | CCAAGGTTAGAGAGTTGGACAC |
| *RPL11* | F | AAAGGTGCGGGAGTATGAGTT |
|  | R | TCCAGGCCGTAGATACCAATG |
| *TFDP1* | F | AATTGAAGCCAACGGAGAACTC |
|  | R | CGGTCTCTGAGGCGTACCA |
| *POLR2A* | F | GGGTGGCATCAAATACCCAGA |
|  | R | AGACACAGCGCAAAACTTTCA |

F: forward primer; R: reverse primer

Table S2. Identification of target genes.

| Gene class | Gene name |
| --- | --- |
| Core | *MCM7, POLE2, GINS4, POLA2, CDC6, GINS1, GINS2, MCM10, ORC6, MCM2, MCM3, MCM5, CDC45, GINS3, CDC7, POLE, MCM6, POLA1, MCM8, MCM4, ORC1, HLA-F, HLA-E, OAS3, HLA-A, IFIT1, IFIT3, MX2, IRF5, OAS2, HLA-B, PSMB8, RPL23, RPL17-C18orf32, PLEC, RPS12, EEF2, RPS29, RPL18, RPL11, RPL12, CDK6, RAD51, E2F3, CCNE2, BRCA1, CREBBP, BRCA2, HIST1H3J, SNRPD1, POLD4, RMI2, TONSL, GEMIN4, BRIP1, ASF1B, BLM, JUN, NUP214, H3F3A, XRCC2, NDC1, HIST1H4L, NUP188, HIST1H4A, NUP205, POLR2A, AGO2, SMARCC1, ARID1A, CDKN1C, TFDP1, E2F1, RBL1, TOP3A, E2F2, PCNA, PRPF8, SRRM2, SRRM1, PRPF19, SF3B4, SF3A1, PRPF40B, PECR, GSTK1, ACOT8, AMACR, HMGCL, PAOX, ATP1B1, ATP1B3, SLC9A1, ATP1A1, ABCA2, ATP1A2, RGS17, RGS7, RGS10, RGS11, RGS14, NCAPD3, SMC2, NCAPH, NCAPG2, NCAPD2, UTP20, FTSJ3, PDCD11, UTP14C, NOL6, ACSS2, ABAT, HIBADH, ALDH6A1, ALDH2, H2AFV, GATAD2A, UBTF, MBD3, RARG, NRIP1, ING5, NCOR2, HIST1H2BM* |
| Hub | *EP300, MAPK3, CREBBP, PCNA, MYC, BRCA1, JUN, POLE, H2AFX, POLE2, RPA2, MCM2, TFDP1, E2F1, PRKACB, GRB2, HIST1H3J, MCM3, POLA1, MCM6, MCM4, MCM7, CDC45, POLR2A, MCM5, HSP90AA1, PLK1, FOS, POLA2, ORC1, CDC6, BLM, SMARCA4, TOP3A, RAD51, SNRPD1, HIST1H2BM, BRCA2, MCM8, AGO2, TIMELESS, HIST1H4L, HIST1H4A, CHD4, H2AFV, MAPK12, IRF5, H3F3A, CDC7, RFC3, MCM10, GINS4, CEBPB, HLA-A, NOTCH1, NUP214, PSMB8, NUP153, HSPA1A, ATF4, EZH2, NUP205, XRCC3, POLD4, GINS3, GINS1, ORC6, TERT, MAPT, DHX9, NCOR2, TNRC6A, NDC1, NUP188, ARID1A, GINS2, TGFB1, RPL11, MEF2C, CDC25A, CEBPA, SMARCC1, IFIT1, MX2, FANCD2* |
| Bottleneck | *MAPK3, HSP90AA1, PRKACB, EP300, MYC, GRB2, CREBBP, JUN, HSPA1A, TERT, MAPT, PLK1, BRCA1, PCNA, TFDP1, TGFB1, AGO2, POLR2A, LDHA, SNRPD1, APOE, NCL, E2F1, AGT, RPL11, DCTN2, PKM, FOS, LRP8, DHX9, BICD2, H2AFX, HMGCS2, DHFR, MAPK12, MUC20, RAB6B, TP73, TKT, ITGA6, EIF4G1, RAB8A* |
| Key | *AGO2, CREBBP, JUN, E2F1, SNRPD1, PCNA, BRCA1, RPL11, TFDP1, POLR2A* |
